# Deciphering the evolution of birdwing butterflies 150 years after Alfred Russel Wallace

**DOI:** 10.1038/srep11860

**Published:** 2015-07-02

**Authors:** Fabien L. Condamine, Emmanuel F. A. Toussaint, Anne-Laure Clamens, Gwenaelle Genson, Felix A. H. Sperling, Gael J. Kergoat

**Affiliations:** 1 University of Alberta, Department of Biological Sciences, Edmonton, T6G 2E9, AB, Canada; 2 SNSB-Bavarian State Collection of Zoology, Münchhausenstr 21, 81247 Munich, Germany; 3 INRA, UMR 1062 Centre de Biologie pour la Gestion des Populations (INRA, IRD, CIRAD, Montpellier SupAgro), 755 avenue du campus Agropolis, 34988, Montferrier-sur-Lez, France

## Abstract

One hundred and fifty years after Alfred Wallace studied the geographical variation and species diversity of butterflies in the Indomalayan-Australasian Archipelago, the processes responsible for their biogeographical pattern remain equivocal. We analysed the macroevolutionary mechanisms accounting for the temporal and geographical diversification of the charismatic birdwing butterflies (Papilionidae), a major focus of Wallace’s pioneering work. Bayesian phylogenetics and dating analyses of the birdwings were conducted using mitochondrial and nuclear genes. The combination of maximum likelihood analyses to estimate biogeographical history and diversification rates reveals that diversity-dependence processes drove the radiation of birdwings, and that speciation was often associated with founder-events colonizing new islands, especially in Wallacea. Palaeo-environment diversification models also suggest that high extinction rates occurred during periods of elevated sea level and global warming. We demonstrated a pattern of spatio-temporal habitat dynamics that continuously created or erased habitats suitable for birdwing biodiversity. Since birdwings were extinction-prone during the Miocene (warmer temperatures and elevated sea levels), the cooling period after the mid-Miocene climatic optimum fostered birdwing diversification due to the release of extinction. This also suggests that current global changes may represent a serious conservation threat to this flagship group.

“*[…] for the purpose of investigating the phenomena of geographical distribution and of local or general variation, […] several groups differ greatly in their value and importance. […] Preeminent among such groups are the diurnal Lepidoptera or Butterflies, whose extreme beauty and endless diversity have led to their having been assiduously collected in all parts of the world […]*” Alfred Russel Wallace^1^.

Situated between the Indian and Pacific Oceans, the region hereafter referred to as the Indomalayan-Australasian Archipelago (IAA) represents the largest and arguably most complex assemblage of islands in the world, housing a substantial proportion of Earth’s biodiversity^2^,^3^. In its heart lie 14 biodiversity hotspots delineated by sharp biogeographical boundaries^2^. One of the most prominent biogeographical barriers is Wallace’s line, demarcating two biogeographical realms: the Asian (Sunda) and the Australian (Sahul) biota. In a seminal paper, Alfred Russel Wallace^4^ was the first to formalize the affinities of two distinct biotic regions separated by the deep Makassar Strait. He found that several communities of birds, mammals and insects were mainly restricted to one side of a narrow geographical area while fewer were found evenly on both sides^4^,^5^. Following Wallace’s pioneering work, other biogeographical boundaries were proposed within the IAA, notably Lydekker’s line, which follows the continental margin of the Australian Shelf^6^,^7^. Currently, the region lying between Wallace’s and Lydekker’s lines, referred to as Wallacea, is acknowledged as a transitional biogeographical unit between the Asian and Australian biotas^3^. Interestingly, Wallace’s line has also been shown to be more permeable to plants and insects, as their greater dispersal ability is thought to have allowed some species to colonize both sides of the line^8^,^9^,^10^,^11^,^12^.

The dynamic geological and climatic history of the IAA generated a matrix of islands in which the distribution of land and sea has been altered extensively through time^13^, alternatively promoting or impeding diversification^14^. The progressive collision of the Sunda and Sahul shelves during the early Miocene engendered high volcanic activity that formed new islands on which diversification may have started^15^. The assembly of Wallacea, especially Sulawesi in the mid-Miocene, may explain the origin and diversification of clades^16^, with Wallacea acting as a biogeographical crossroads between Sahul and Sunda biotas and/or as an evolutionary cradle of diversity (or species-pump) fostering rapid radiation with higher speciation rates. Repeated sea-level fluctuations^17^, promoting habitat alteration or formation, colonization of new niches and ultimately allopatric speciation, have acted as an important driver in the IAA^8^,^11^,^14^. Clade characteristics that allow continual adaptation and diversification within a region may also govern the current species richness such that diversity-dependent processes and ecological or geographical carrying capacity confer some limits to diversification^5^,^15^, especially in the context of island biogeography^4^. To better tease apart the processes underlying the megadiversity observed in the IAA it is essential to account for the historical legacy of the region and the evolutionary history of groups found on each side of Wallace’s line^3^.

One hundred and fifty years ago, Wallace^1^ published one of his most influential papers, in which he used butterflies of the family Papilionidae as a model system to describe and understand species richness across the entire IAA. He focused on the charismatic giant birdwings, a well-studied clade of large and brightly coloured butterflies comprising three extant genera and about 37 species distributed from India to Australia^18^, with numerous island endemics harbouring unique wing patterns (Supplementary Fig. S1). Birdwing species are iconic species for invertebrate conservation; several species are considered endangered and benefit from strict conservation policies. The Convention on International Trade in Endangered Species of Wild Fauna and Flora (CITES) has listed all species on annex II (except *Ornithoptera alexandrae* listed on the annex I). The International Union for the Conservation of Nature (IUCN) red list of threatened species recognizes eight threatened species belonging to *Ornithoptera* (although *O. paradisea* is listed as Lower Risk/Least Concern), and three *Troides* species (*T. andromache* as Lower Risk/Near Threatened, *T. dohertyi* as Vulnerable, and *T. prattorum* as Vulnerable). The IUCN red list lists only three species (*O. alexandrae*, *O. croesus* and *O. meridionalis*) as Endangered.

Ever since Wallace^1^, birdwing butterflies have become a prime biological model to discriminate factors driving island speciation and distribution patterns in the IAA^18^,^19^,^20^,^21^,^22^,^23^. Their distinct biogeographical pattern across Wallacea makes them valuable for studying the geological assembly of the region and the ecological opportunities provided by emerging islands (Fig. 1). The genus *Ornithoptera* (14 species) is endemic to the Melanesian region, where species diversity peaks in New Guinea, although a few species are found on the other side of Lydekker’s line (e.g. the Wallace’s Golden Birdwing *O. croesus* in Halmahera). This genus includes the two largest extant species of butterfly, Queen Alexandra’s Birdwing (*O. alexandrae*) and the Goliath Birdwing (*O. goliath*), which have a wingspan of up to 28 centimetres. The genus *Trogonoptera* (two species) is restricted to the west of Wallace’s line, where *T. brookiana* occurs in the Greater Sunda Islands (Borneo, Sumatra, and Java) and *T. trojana* in Palawan. The genus *Troides* (21 species) has most of its species diversity in the Indomalayan region but presents an extensive distribution going northward to India (*T. minos*), eastward to the Philippines (*T. rhadamantus*) and has some species crossing through Lydekker’s line into the Australian region (*T. oblongomaculatus* from Sulawesi to New Guinea). Birdwings are generally strong fliers^1^,^19^ and highly specialized on Aristolochiaceae^18^,^19^,^20^, which are usually toxic for herbivores^24^.

Despite his difficulties in disentangling species boundaries^25^, Wallace^1^ used estimates of species richness to hypothesize that the Greater Sunda Islands (especially Borneo) were the ancestral area of this clade from which it later dispersed towards Australia (a similar pattern has also been found in other clades^3^). Wallace postulated that ‘settling down’ was a strong factor in island speciation (here referred to as founder-event speciation), especially for these good dispersers, and further argued that island features and environmental factors subsequently fostered morphological variation and eventually contributed to speciation (corresponding to the hypothesis of Wallacea acting as a species pump). Yet the main triggers responsible for the diversification of these emblematic butterflies remain equivocal.

Building on the pioneering work of Wallace, we revisit some of his conclusions. Here we rely on a dated phylogeny of a comprehensive sampling of birdwings to estimate what processes shaped their current biogeographical pattern, using novel approaches^26^,^27^. We also implement distinct sets of diversification analyses in order to: *(i)* estimate the influence of Wallacea on birdwing speciation and extinction rates; *(ii)* test whether their species richness is still expanding today or has reached an equilibrium suggesting that birdwing diversification may have been influenced by diversity-dependent processes; and *(iii)* assess the impact of palaeo-environmental variables (temperature and sea level) on diversification rates.

## Results

### Phylogeny, species monophyly, and dating

Bayesian phylogenetic estimations yielded resolved trees indicating that all three genera and most species are monophyletic with high support (Supplementary Text S1 and Supplementary Fig. S2). The only marked exception was *Troides haliphron* that was recovered as paraphyletic, suggesting that two molecular lineages with different evolutionary trajectories may be recognised (*T. h. haliphron* plus *T. h. purabu* occurring in South Sulawesi, and *T. h. socrates* plus *T. h. naias* in Sumbawa to Alor islands, Supplementary Fig. S2). This pattern was independently recovered using different molecular datasets and as a result we provisionally recognize two species within *T. haliphron*. The first putative *haliphron* species would include the subspecies in the Sulawesi region, i.e. *T. h. haliphron* (Southwest Sulawesi), *T. h. purabu* (Batuata Island), *T. h. pistor* (Bonerate, Jampea, Kalaotoa, and Madu islands), *T. h. pallens* (Selayar Island) and *T. h. eleonorae* (Buton Island). The second putative *haliphron* species would comprise the two subspecies *T. h. naias* and *T. h. socrates*, occurring in the Lesser Sunda Islands (Alor, Flores, Solor, Sumba, Sumbawa, and Wetar islands), and possibly *T. h. bellwoni* (Lucipara Islands, Moluccas).

**Figure 2:**
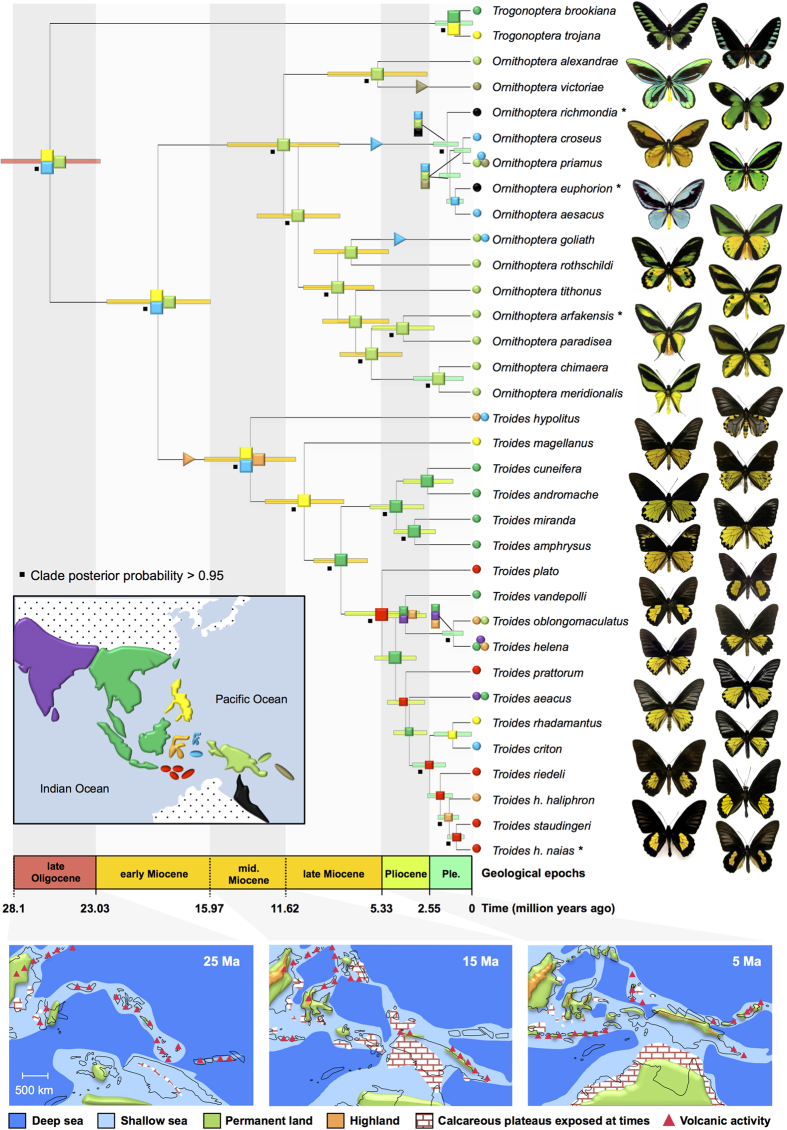
Dated Bayesian phylogeny of the birdwing butterflies radiation and palaeo-tectonic evolution of the Indomalayan-Australian Archipelago. Coloured squares on nodes indicate biogeographical location as in the inset map, with triangles indicating colonizations. A timescale is shown spanning the full evolutionary history of the group. Panels at the bottom include maps of the distribution of land and sea at respectively 25, 15 and 5 Myr ago *sensu*^13^,^15^. The asterisk next to terminal taxon names indicates those not figured. Pictures of birdwing butterflies made by Fabien Condamine. Maps drawn with PowerPoint by Fabien Condamine using various sources (e.g. Ref. 13,15).

We assembled a species-level dataset comprising 4,395 nucleotides (five genes) for 34 birdwing species and 31 outgroups that was used to conduct simultaneous inferences of species-level phylogenetics and divergence time estimates. The species-level phylogenetic tree yielded by the BEAST analysis was fully resolved and presented robust node supports (Fig. 2). We found that *Trogonoptera* was sister to the two other genera, and that the three subgenera within *Ornithoptera* (i.e. *Aetheoptera, Ornithoptera*, and *Schoenbergia*) were monophyletic, supporting previous morphological and molecular studies^21^,^22^,^23^. The phylogeny of *Ornithoptera* is highly congruent with the previous work of Morinaka *et al.*^21^, who only used the mitochondrial gene ND5. Despite some previous studies that lumped *Ornithoptera*, *Troides* and *Trogonoptera* into a single genus^1^,^19^, we follow the views of several recent authors that advocate maintaining the taxonomic rank of the three genera based on morphological, ecological and distributional data^18^,^21^,^22^,^28^. We inferred an origin of birdwings in the Oligocene around 25.8 Myr ago (95% HPD 22.2–29.9 Myr ago). *Ornithoptera* and *Troides* diverged in the early Miocene around 19.3 Myr ago (95% HPD 16.3–22.8 Myr ago). Both genera diversified in the middle Miocene around 11.5 Myr ago (95% HPD 8.4–15.3 Myr ago), and 13.6 Myr ago (95% HPD 10.8–16.4 Myr ago), respectively. These results are independent of the speciation tree prior used (Supplementary Text S1).

### Biogeography and diversification rates

Biogeographical maximum likelihood analyses (unconstrained by a stratified model) statistically supported models in which founder-event speciation was allowed (Supplementary Text S1). In all models, the founder-event parameter significantly improved the likelihood of biogeographical estimations (ΔAICc > 10 for all pairs; see Supplementary Fig. S3 and Supplementary Table S1). The biogeographical estimations with the best model (DEC+J) is presented in Fig. 2, indicating that the birdwing ancestor originated in the vicinity of proto-Halmahera, New Guinea and the Philippines. The results of diversification rate analyses are summarized in Table 1. We found that a growing species diversity that progressively reached diversity equilibrium influenced the overall rates of birdwings diversification. Diversity-dependence models estimated a carrying capacity similar to the current species richness of the clade (this pattern is also recovered for *Ornithoptera* and *Troides*, analysed separately, Supplementary Table S2 and Supplementary Fig. S4). A diversity-dependent extinction model was recovered as the best best-fitting diversity-dependence model (ΔAICc ranged between 2.5 and 17), despite having received little empirical support in other comparable studies^29^. We found that Wallacea did not promote speciation rates but rather triggered dispersal (increased transition rates) towards other areas, as the best-fitting GeoSSE model was one in which only transition rates varied and both speciation and extinction remained equal among the two areas (ΔAICc ranged between 2.2 and 8.4, Supplementary Table S3). Transition rates (or dispersal rates in this context) were estimated to be 10-fold higher from Wallacea towards the remaining areas, compared to the other direction, suggesting an important role of Wallacea as a biogeographical crossroads rather than a species pump (Supplementary Fig. S5).

**Figure 3:**
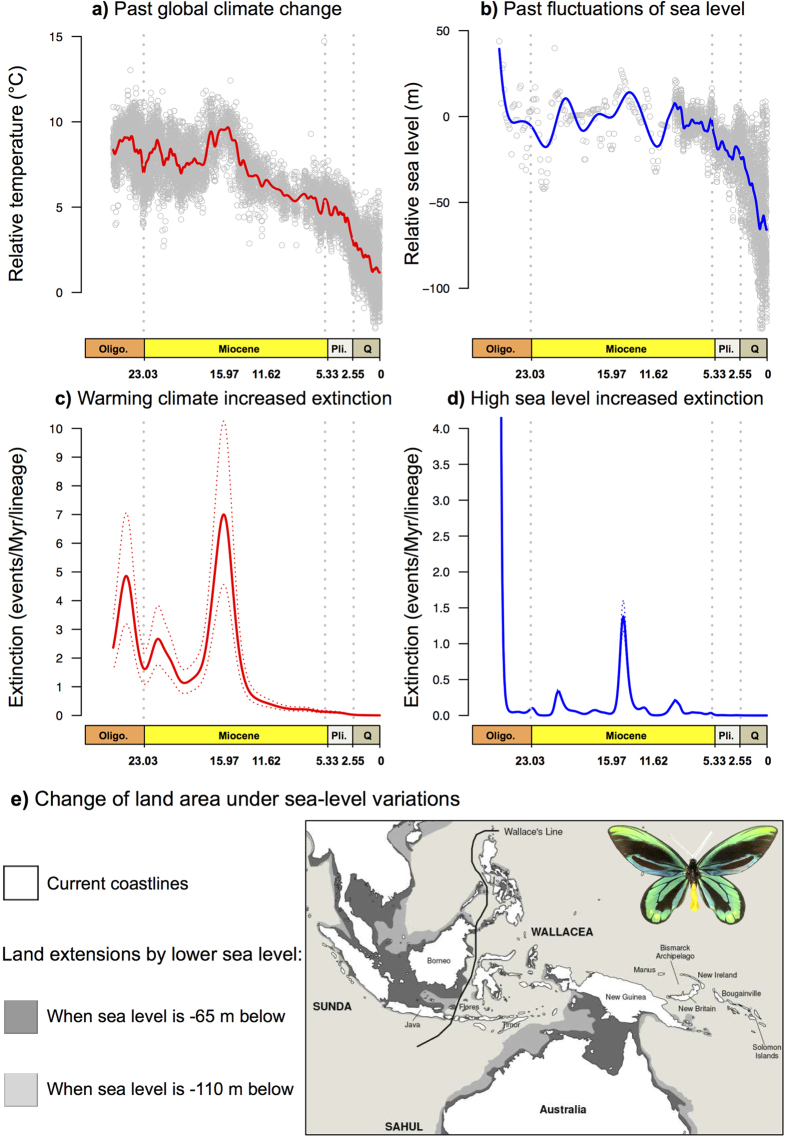
Evaluation of the effect of environmental changes on diversification processes in the evolutionary history of birdwing butterflies. (**a**) Major trends in global climate change^46^, and (b) major trends in global sea level^17^ during the last 26 Myr, estimated from relative proportions of different oxygen isotopes. (**c**) Best model for temperature-dependent diversification with a positive relationship between extinction rate and palaeo-temperatures. (**d**) Best model for a sea level-dependent diversification with a positive relationship between extinction rate and past sea levels. Dotted lines indicate the maximum and minimum limits of the confidence interval obtained from 500 phylogenies, and the continuous line represents the average. (**e**) The Indomalayan-Australasian Archipelago when sea level varied in the past, and under two projections of sea-level decrease. Pictured is *Ornithoptera alexandrae*, a threatened species living in the coastlines of Southeast Papua New Guinea. Oligo., Oligocene; Pli., Pliocene; and Q, Quaternary. Picture of the birdwing butterfly made by Fabien Condamine. Map drawn with PowerPoint by Fabien Condamine.

Analyses of diversification rates as a function of palaeo-environment suggested that past sea level and temperature fluctuations better explained diversification within birdwings (Fig. 3). The analyses indicated that temperature and sea-level changes have had similar impacts on the diversification of birdwing butterflies (ΔAICc ranged between 1.9 and 2.6, Supplementary Table S4). Models testing the influence of past sea-level fluctuations suggested that the best-fitting model had a constant speciation rate (not affected by sea-level fluctuations) while extinction was positively correlated to past sea-level fluctuations such that higher sea level generated higher extinction, and *vice versa* (Fig. 3). The models built to assess the impact of palaeo-temperature fluctuations showed that the best fitting model is the one in which speciation remained constant (and was not affected by temperature fluctuations) whereas extinction was positively linked to past changes in temperature such that elevated temperature increased the extinction rate, and *vice versa* (Fig. 3).

We keep in mind that the data do not distinguish strongly between the models, especially for the palaeo-environment models. The ∆AICc and Akaike weights are in the category ‘barely worth mentioning’ according to commonly-used scales, but models where ΔAICc is in the 2–7 range have some support and should rarely be dismissed. It is interesting that the tests for an effect of the palaeo-environment provide some support to models with varying extinction, although this cannot be considered a very confident conclusion given the available phylogenetic data.

## Discussion

The hypothesis of Wallacea acting as a species pump, in which *in situ*-speciation triggers diversification, has rarely been tested. Yet, Wallacea is recognized as a major evolutionary arena for biodiversity^16^. Wallace^1^ proposed that Sulawesi was a keystone of butterfly evolution because it contains a remarkable diversity of endemics^30^ and stands upon the dividing line between two biogeographical regions. Our dating analyses indicate that birdwing butterfly speciation on Wallacea did not occur before the Miocene, which is consistent with geological evidence^13^. The timeframe of Sulawesi colonization by birdwing butterflies (mid-Miocene) and speciation on the island (mid-Miocene/Pliocene) is also fully compatible with data presented in a meta-analysis of this island^16^. However, our data and analyses do not support the role of Wallacea as a trigger for speciation because no difference in speciation and extinction rates was recovered by our diversification analyses. Both biogeographical and diversification analyses instead suggest that Wallacea was the source of numerous dispersal events towards neighbouring areas (Sahul and Sunda). In this context, the Pliocene onset of periodic sea-level changes may have played an important role by increasing dispersal from Sulawesi during periods of low sea level or by increasing the isolation of species/populations, and therefore facilitating allopatric speciation during periods of elevated sea level. Altogether, these results support Wallace’s view on the role of Wallacea as a biogeographical pathway for butterflies, although these biogeographical events did not impact birdwing diversification rates *per se*.

Although Wallace did not provide a formal hypothesis for the origin of birdwing butterflies, our biogeographical estimations tend to support the hypothesis of a complex ancestral origin lying between the Philippines, the Moluccas and New Guinea. The complexity of the geological history of the IAA region makes it difficult to reliably know the past distribution of land and sea at a given point in time^13^,^15^. Prior to 25 Myr some palaeo-islands, or terranes, were possibly aerial at that time and may have harboured the birdwing ancestor (see the inset maps in Fig. 2). The Moluccas and New Guinea, to some extent, share a common geological origin as part of the Australian Plate whereas some islands of the Philippines and the northernmost part of New Guinea are of oceanic origin, but all from the same geographical area^13^. We suggest that an occurrence of the birdwing butterfly ancestor on a proto-Papuan arc stretching from the westernmost part of the Sula Spur to central New Guinea is a scenario consistent with “island hopping”, in which ancient islands provided discrete temporal and spatial context for colonization and speciation and greatly extend the temporal window theoretically available for insular evolution^31^. Based on the distribution of birdwing species/subspecies, Wallace^1^ was convinced that settling down (dispersal and establishment into a new island) was the main process of diversification in the region. Yet, recent studies showed that dispersal does not appear to have played a major role in avian diversification in the IAA^32^. However, we found strong evidence indicating that birdwing distribution is mostly explained by founder-event speciation with Wallacea as a main source or pathway. This result seems very likely given the dispersal abilities of many species and the nature of the IAA, a complex assemblage of thousands of islands that have almost all been colonized by these butterflies^18^. For instance, this pattern is particularly obvious within *Troides* that repeatedly colonized the Lesser Sunda Islands from Greater Sunda Islands (and *vice versa*) in the late Miocene and Pliocene (Fig. 2). Contrary to other butterflies^11^, vicariance had a lower impact on diversification. Only three vicariant events were inferred, the divergences: *(i)* between *Ornithoptera* and *Troides* in the early Miocene, *(ii)* between *T. hypolitus* and the remaining *Troides* in the mid-Miocene, and *(iii)* between the two *Trogonoptera* species in the Pleistocene. The timeframe of the two first vicariant events corresponds well with the build-up of Sulawesi^13^,^16^, and the third is probably attributable to Pleistocene sea-level changes^14^. Biogeographical analyses further indicated multiple transgressions of Wallace’s line in *Troides* (none in *Ornithoptera*), and of Lyddeker’s line in *Ornithoptera* (one in *Troides*). This result suggests a higher permeability to dispersal across Wallacea. Interestingly, this pattern was also recovered in other groups of butterflies^11^,^33^,^34^, beetles^12^ and plants^10^. However, we did not find evidence (contrary to palms^10^) for variation in speciation and extinction rates that can be attributed to geological changes or the crossing of Wallace’s and Lyddeker’s lines. Founder-event speciation, likely accounted for by the strong dispersal abilities of birdwing butterflies, has probably allowed these insects to continuously colonize remote islands whenever they appeared and to establish insular radiations as Wallace predicted. For instance, *Troides haliphron* has reached the remote and isolated Lucipara Islands (*Troides h. bellwoni*).

It is often thought that a long-term stable environment leads to lower extinction and, therefore, greater biodiversity^5^, a hypothesis also known as the museum model of diversity. Recent work proposed that geologically dynamic regions play a major role in diversification^15^,^35^,^36^. In the ecosystems of the IAA, geological studies suggested that tectonic events such as the building of Sulawesi and the Philippines, as well as the orogeny of New Guinea, have caused large-scale landscape and climatic changes in the last 15 Myr^13^,^33^. Alternatively, geologically dynamic regions may have destroyed previous habitats, leading to extinction (contrary to long-term stable environments). In the context of island biogeography and the IAA region, the rise and fall of sea levels through time is an important factor affecting opportunities to diversify because it creates (low sea level) or erases (high sea level) areas where radiations took place. We found evidence for higher extinction rates during warm climates and elevated sea levels. Ali and Aitchison^31^ examined the impact that geological and climatic events (namely island ontogeny and shifting sea levels) had on biodiversity in the Galapagos Islands. The authors proposed that the dynamics of isolation caused by geological and climatic processes play a fundamental part in shaping diversity. Our study on birdwing butterflies and other studies on distinct groups of butterflies^11^,^33^,^34^,^37^ support this hypothesis. For instance the ancestor of *Ornithoptera victoriae* has likely arisen by a dispersal event from mainland New Guinea to the Solomon Islands via the Bismarck Archipelago, followed by subsequent extinctions in the Bismarck Archipelago that can possibly explain the absence of the subgenus *Aetheoptera* in these islands (Fig. 2). In addition, we underline the important role of extinction in this mechanism because suitable habitats were probably continuously destroyed by successive environmental changes, resulting in the likely extinction of species unable to disperse or adapt. Surviving lineages were successful dispersers able to cope and rapidly adapt to changing conditions and/or newly colonized environments.

To explain differences in birdwings’ species richness at both taxonomic and biogeographical scales, we can postulate the role of ecological limits to diversification. Under this hypothesis clade diversity is regulated by ecological factors (species richness is independent of clade age), such as diversification rates slowing down as diversity increases because opportunities for speciation concurrently decrease. Competitive interactions between congeneric butterfly species are a strong prediction of Wallace’s work^1^. Support for the latter hypothesis is provided by our results, which indicate that diversity-dependent processes are at play in birdwing butterflies. We found that extinction increases when diversity increases. The parallel increase of extinction and diversity is potentially explained by a decrease of population size per species as the number of species increases^38^. Diversity equilibrium was apparently reached faster in Sahul (for the radiation of *Ornithoptera*) rather than in Sunda (for the radiation of *Troides*) as suggested by our estimations of carrying capacities (more elevated in Sunda, Table 1). A smaller carrying capacity for *Ornithoptera* may explain why there are more species in *Troides* (with no differences in ages or time-dependent diversification rates). This finding might be rationalized by an area-size effect since *Ornithoptera* have only radiated in the Sahul Shelf (particularly New Guinea) and have not succeeded in colonizing the Sunda Shelf (contrary to *Troides*). Differences in host plants within the family Aristolochiaceae might also explain the differences in diversity because *Troides* feed on the genus *Aristolochia*, which is widespread and diverse in all of the IAA, whereas *Ornithoptera* feed on the subgenus *Pararistolochia*, with only a few species restricted to Sahul (*Aristolochia* plants in other subgenera can be lethal for *Ornithoptera*^20^).

This study also gives us the opportunity to assess whether phylogenetic inferences of past diversification dynamics may help to understand the current biodiversity crisis^39^. Global environmental changes that are principally due to human activities contribute to eroding biodiversity directly or indirectly, notably through the warming event we are currently experiencing. In the case of birdwings, the combination of high sea levels and high temperatures apparently led to drastic periods of extinction in the late Oligocene and in the early to mid-Miocene. If the diversification pattern that we inferred holds over macroevolutionary timescales (i.e. phylogenetic conservatism), it suggests that their current biodiversity is threatened by ongoing changes. In this case, the mid-Miocene would be an ancient analogue for current changes. However, comparing past and current effects of environmental changes on biodiversity is complicated by differences between human-driven environmental changes and long-term natural processes. Harnik *et al.*^40^ compiled information on the drivers of ancient and modern marine extinctions, and found that some drivers are shared with past and current environmental conditions, while additional pressures such as overexploitation and pollution are new threats. The two most important pressures on current biodiversity are habitat loss and climate change^40^. Macroevolutionary analogies to habitat loss include abrupt climatic events, sea-level increase, or major ecological transitions, which may be similar to human-driven habitat degradation and loss today. Hence, although analogies between past and present environmental changes are sometimes highly speculative, they can be relevant to making predictions on the fate of biodiversity^40^.

Conclusions about the nature of diversification depend upon the quality of the underlying data. Incomplete taxon sampling, poor divergence time estimations and small-sized phylogenies are potentially serious problems that are difficult to address. These can lead to inaccurate parameter estimates or wrong model selections. Such methodological limitations may constrain the certainty of our interpretations and conclusions. As long as the fundamental hypothesis testing nature of these analyses are kept in mind, they still remain our best window into understanding the rich, deep past of Earth’s stupendous biological diversity. We hope that our approach will provide interesting perspectives for future investigations on other model groups with larger phylogenies. Both climate and sea-level variation, coupled with the formation of Wallacea as a biogeographical crossroads, have often been considered to be strong diversification drivers, but their combined contribution has rarely been demonstrated. We have provided insights into the effect of climatic and geological events, consistent with a pattern of spatio-temporal habitat dynamics that continuously created/erased suitable habitats. Given the prevalence of environmental fluctuations that offer temporal habitat opportunities at various timescales in nature, this result has important implications for understanding diversification patterns in many natural systems.

## Methods

### Taxon sampling and molecular dataset

About 90% of birdwing species were included (Supplementary Table S5). This sampling encompasses all described genera and subgenera, including 182 specimens of birdwing butterflies representing all 14 known *Ornithoptera* species, 17 of 21 *Troides* species, and the two species of *Trogonoptera*. Only four species (*Troides darsius*, *T. dohertyi*, *T. minos* and *T. plateni*) were not sampled. Most specimens come from museum collections and permits were obtained by the first author for species included in CITES or IUCN Red lists. Based on the most comprehensive phylogeny of Papilionidae^41^, we also included representatives from all remaining Troidini genera in order to implement secondary calibrations. Thirty-one species of Troidini (from *Atrophaneura*, *Battus, Cressida, Euryades, Pachliopta* and *Pharmacophagus*) were added to the dataset for that purpose. Three mitochondrial genes (cytochrome oxidase I, NADH dehydrogenase 5, and 16S rRNA) and two nuclear protein-coding genes (elongation factor-1a and wingless) were used. DNA sequences were either downloaded from GenBank^21^,^23^,^41^,^42^ or newly obtained using standard molecular protocols^42^. Taxon sampling and GenBank accession numbers are given in Supplementary Table S5.

### Phylogenetics and dating

Phylogenetic relationships were reconstructed under Bayesian inference (Supplementary Text S2). Gene trees were then used as a blueprint to generate a species-level dataset (with one specimen per species). Based on this reduced dataset we estimated divergence times with an uncorrelated lognormal Bayesian relaxed-clock approach. We used secondary calibrations based on a fossil-calibrated phylogeny of Papilionidae^41^. Uniform distributions enforced strict maximum and minimum ages based on the confidence intervals of six nodes (Supplementary Text S2).

### Biogeography

The estimation of ancestral areas was carried out with the model BioGeography with Bayesian (and likelihood) Evolutionary Analysis of RangeS (BioGeoBEARS^27^). Species ranges were coded by presence–absence (excluding marginal distribution). The biogeographical model included nine component areas (see the inserted map on Fig. 2) with respect to regional palaeogeographical evidence^13^. While BioGeoBEARS allows building time-stratified biogeographical models with time slices representing major periods of geological rearrangements, we did not use such models because of the high geological complexity of the region. Many uncertainties, in particular regarding Halmahera, New Guinea, the Philippines and Sulawesi, remain about the appearance or disappearance of islands, and the timing of these geological events still represents a challenge^13^,^15^,^43^. Birdwings are also such good dispersers that they have colonized almost the entire IAA (Supplementary Text S2). Therefore the use of a subjective dispersal rate matrix was avoided for these analyses. We tested the dominant process(es) (range expansion followed by allopatry or founder-event speciation) involved in the biogeographical dynamics by comparing six distinct models including or excluding founder-event speciation and vicariance^27^.

### Diversification rates

In order to take into account both phylogenetic and dating uncertainties, we used 500 randomly chosen trees from the BEAST post-burnin posterior distribution to estimate diversification rates with different methods. See Supplementary Text S2 for more details in each model of diversification.

We estimated the influence of Wallacea on speciation and extinction rates using the likelihood approach called Geographic State Speciation and Extinction (GeoSSE)^44^ implemented in the R package *diversitree*^45^. This likelihood-based approach allows the estimation of region-dependent rates of speciation, extinction, and range evolution from a phylogeny, using a model that combines features of constant-rates birth-death models with a three-state Markov model^44^. We coded each species according to its distribution using three distinct states: Wallacea, outside Wallacea or widespread. Life history data was obtained from the taxonomic literature and museum records. We constructed the likelihood functions representing 12 different evolutionary models for the relationship between geographical distributions and diversification rate. To support the Wallacean species-pump hypothesis, the best model should demonstrate a higher diversification rate for Wallacea.

We tested the hypothesis that diversification was rapid in the early stage of the group as expected under the ecological opportunities provided by the emergence of regional islands. Secondly, this diversity may have reached an equilibrium (or has been bounded), meaning that it is saturated towards the present and diversification rates slowed down. We thus explored the effect of diversity on speciation and extinction rates using the method of Etienne *et al.*^29^. We built five models: *(i)* speciation declines linearly with diversity and no extinction, *(ii)* speciation declines linearly with diversity and extinction, *(iii)* speciation declines exponentially with diversity and extinction, *(iv)* extinction increases linearly with diversity, and *(v)* extinction increases exponentially with diversity. The initial carrying capacity was set to the current species diversity, and the final carrying capacity was estimated according to the models and relative parameters. These analyses were performed for the whole birdwing butterfly clade and the genera *Ornithoptera* and *Troides* separately.

Finally, we evaluated the impact of palaeo-environment on diversification rates using an environmental-dependence model^46^. This model estimates the effect that a given environmental variable may have on diversification, by assessing whether speciation or extinction rates have a particular dependency with the variable of interest. For example, in the case of exponential dependency on temperature, a positive estimated α would indicate that higher temperatures enhance speciation, whereas a negative α would indicate that higher temperatures hamper speciation^46^. The impact of changes in past sea levels and palaeo-temperatures was tested using palaeo-environmental data compiled from Miller *et al.*^17^ for past sea levels and from Zachos *et al.*^47^ for palaeo-temperatures. Six models were designed for each palaeo-environmental variable to assess speciation and extinction that may vary or not as a function of changes in palaeo-temperatures or past sea levels.

## Additional Information

**How to cite this article**: Condamine, F. L. *et al.* Deciphering the evolution of birdwing butterflies 150 years after Alfred Russel Wallace. *Sci. Rep.* **5**, 11860; doi: 10.1038/srep11860 (2015).

## Supplementary Material

Supplementary Information

## Figures and Tables

**Figure 1:**
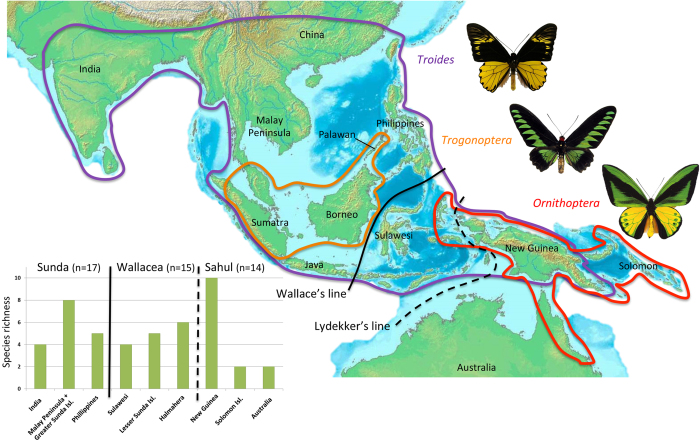
Distributional pattern of birdwing butterflies originally described by Wallace showing species richness west and east of Wallace’s and Lydekker’s lines, and in each important biogeographical unit of the Indomalayan-Australian Archipelago used here. Pictures of birdwing butterflies made by Fabien Condamine. Map drawn with PowerPoint by Fabien Condamine.

**Table 1:** Summary of the analyses of diversification that highlights the main results.

Type of birth-death	Methods used	References	Data used	Settings	Results
Trait-dependence (rates vary as a function of a character state for a trait)	GeoSSE (*make.geosse*)	Goldberg *et al.*^44^	500 posterior trees + geographical occurrence (Wallacea endemic, endemic to the remaining region, or widespread across the archipelago)	12 models to test whether a character state impacted speciation and/or extinction and/or transition rates between Wallacea and the remainder	Transition rate out of Wallacea is 10-fold more elevated than transition rate into Wallacea (no effect of the trait on diversification rate)
Diversity-dependence (rates vary as a function of the number of species)	DDD (*dd_ML*)	Etienne *et al.*^29^	MCC tree of the whole tree, and MCC trees of *Ornithoptera* and *Troides* (to see if they reach their carrying capacity)	5 models to test whether speciation declines with diversity and/or extinction increases with diversity	All clades have reached their carrying capacities, with extinction increasing as diversity increases
Environmental-dependence (rates vary as a time-variable environment)	Condamine *et al.*’s approach	Condamine *et al.*^46^	500 posterior trees + past temperatures^47^ + past sea levels^17^	6 models (3 for each environmental variable) to test whether rates vary or not (exponential variation)	Extinction rate is positively associated with warm climate and elevated sea level

Notes: MCC = maximum clade credibility.
