# Supplementary material for: Deciphering the evolution of birdwing butterflies 150 years after Alfred Russel Wallace
Source: Sci Rep. 2015 Jul 2;5:11860. doi: 10.1038/srep11860 (PMC4488763; doi:10.1038/srep11860)

Supplementary Material

Deciphering the evolution of birdwing butterflies 150 years after Alfred Russel Wallace

Fabien L. Condamine, Emmanuel F.A. Toussaint, Anne-Laure Clamens, Gwenaelle Genson, Felix A.H. Sperling and Gael J. Kergoat

__Contents__

Supplementary Text S1. Expanded Results.

Supplementary Text S2. Expanded Materials and Methods.

Supplementary Table S1. Results of biogeographical analyses.

Supplementary Table S2. Results of diversity-dependence diversification analyses.

Supplementary Table S3. Results of trait-dependence (GeoSSE) diversification analyses.

Supplementary Table S4. Results of palaeo-environmental-dependence diversification analyses.

Supplementary Table S5. Taxon sampling and GenBank accession numbers.

Supplementary Figure S1. Geographic distribution of *Ornithoptera priamus* and its subspecies (a), and *Troides helena* and its subspecies (b).

Supplementary Figure S2. Bayesian phylogenies of specimen-level dataset for single genes (in order: COI, ND5, 16S, and EF-1α) for assessment of species monophyly.

Supplementary Figure S3. Biogeographical reconstructions using DEC, DEC+J, DIVA-like, DIVA-like+J, BayArea-like and BayArea-like+J models.

Supplementary Figure S4. Lineages-through-time plots of birdwing diversity.

Supplementary Figure S5. Bayesian MCMC of the best fitting GeoSSE model.

Supplementary Text S1. Expanded Results

Molecular dataset and partitioning strategies

The specimen-level datasets included DNA sequence from 124 specimens of birdwing butterflies for the COI gene (1,548 nucleotides), 92 specimens for the ND5 gene (907 nucleotides), 61 specimens for the 16S (518 nucleotides), and 67 specimens for the EF-1α gene (600 nucleotides). The best-fit partitioning scheme for COI (BIC_{linked}=17800.19 and $BIC_{unlinked}$=18749.47) was a separate partition for each codon and its own substitution model, as follows: first codon (TrN+G), second codon (HKY+G), and third codon (TIM+G). The best-fit partitioning scheme for ND5 (BIC_{linked}=11399.75 and $BIC_{unlinked}$=11798.65) was two partitions: one for the first and third codon (HKY+I+G) and one for the second codon (TIM+G). The best-fit substitution model for 16S was a GTR+I+G. No partitioning scheme is possible with a ribosomal RNA gene. The best-fit partitioning scheme for EF-1α (BIC_{linked}=3971.64 and $BIC_{unlinked}$=4147.06) was a separate partition for each codon and its own substitution model, as follows: first codon (TrN+I), second codon (JC+I), and third codon (K80+G). The final dataset contained 182 birdwing specimens, of which 107 are *Troides*, 68 are *Ornithoptera* and 7 are *Trogonoptera*

Species monophyly

Many old specimens failed to amplify several genes, resulting in missing data and impeding our creation of a supermatrix including all specimens for all genes. However the gene tree approach still allows an effective solution to testing species boundaries with independent analyses.

When reconstructing the gene trees, we used the partitioning scheme and among-site rate variation suggested by PartitionFinder (see below) but instead of selecting one

substitution model a priori, we used reversible-jump Markov Chain Monte Carlo (rj-MCMC) to allow sampling across the entire substitution rate model space (see Supplementary Text S2). We also performed phylogenetic analyses using the substitution model provided by PartitionFinder, and we found identical results with phylogenies estimated using rj-MCMC (results not shown).

Within *Ornithoptera*, all recognized morphological species had monophyletic gene trees. The fact that all morphological species (including the species in the subgenus *Ornithoptera* (five species)) exhibit reciprocal monophyly allowed us to maintain the species status of these species. While some of the species, like *Ornithoptera goliath*, show strong mtDNA divergence among subspecies that may lead us to consider two putative species (one in the Moluccas and one in New Guinea) for the macroevolutionary analyses, we preferred to use a conservative approach and retain the morphological species.

In *Troides*, all morphological species had monophyletic gene trees, except one species. *Troides haliphron* was recovered as paraphyletic for both mtDNA and nucDNA due to the inclusion of *T. staudingeri*. This pattern holds regardless of the molecular dataset. This result suggests that two molecular lineages with different evolutionary trajectories can be recognised as they have a disjunct distribution pattern in Wallacea, with *T. haliphron* subspecies *haliphron* and *purabu* occurring in South Sulawesi and the Selayar Islands, whereas *T. haliphron* subspecies *socrates* and *naias* occur in in the Lesser Sunda archipelago (Sumbawa to Alor islands)[1]. Consequently, we treat the two clades of *T. haliphron* as two putative species. Divergence between *T. helena* and *T. oblongomaculatus* is recent. Only mtDNA revealed detectable molecular differences between the two species (no genetic variation for the nucDNA). The data suggest either a recent speciation event or that the two species are incipient species (speciation not completed).

The results of the phylogenetic analyses for each gene (except the wingless gene since we obtained only limited taxon sampling) are presented in the Supplementary Fig. S2.

Species relationships, dating and difference between the speciation tree prior

All Bayesian analyses of the species-level dataset inferred the same topology. The genus *Trogonoptera* is found as sister to the two other genera with the highest support. Within the two species-rich genera, the molecular relationships strongly agree with previous morphological works (e.g.[2-4]). Node support values are good to high despite the incompleteness of our molecular matrix (≈60% complete), with ≈70% of nodes with posterior probability $\geq$ 0.95. The four missing species can be assigned based on morphology: *T. darsius* is considered as closely related to the *T. haliphron* group, *T. minos* is likely to be closely related to *T. helena*, whereas *T. dohertyi* and *T. plateni* are thought to constitute the sister clade of *T. rhadamantus*. Adding those species in future should clarify some relationships within these groups and will likely strengthen phylogenetic support within *Troides*.

Dating with the Yule prior. We inferred an origin of birdwings in the late Oligocene around 25.9 Myr ago (95% HPD 22.2-29.9 Myr ago). The genera *Ornithoptera* and *Troides* diverged in the early Miocene around 19.3 Myr ago (95% HPD 16.3-22.8 Myr ago). Both genera diversified in the middle Miocene around 11.5 Myr ago (95% HPD 8.4-15.3 Myr ago), and around 13.6 Myr ago (95% HPD 10.8-16.4 Myr ago), respectively. The genus *Trogonoptera* diversified in the Pleistocene about 1.04 Myr ago (95% HPD 0.25-2.4 Myr ago).

Dating with the birth-death prior. We inferred an origin of birdwings in the late Oligocene around 25.8 Myr ago (95% HPD 22.1-29.9 Myr ago). The genera *Ornithoptera* and *Troides* diverged in the early Miocene around 19.1 Myr ago (95% HPD 16-22.6 Myr

ago). Both genera diversified in the middle Miocene around 11.4 Myr ago (95% HPD 8.6-15.3 Myr ago), and around 13.5 Myr ago (95% HPD 10.8-16.4 Myr ago), respectively. The genus *Trogonoptera* diversified in the Pleistocene about 1.03 Myr ago (95% HPD 0.26-2.3 Myr ago).

The close correspondence between dates obtained with Yule and birth-death prior indicates that the choice of the speciation tree prior had no effect on age estimates. However, their marginal likelihood estimates are significantly different based on a Bayes factor > 6.0 (MLE of the Yule prior = -28967.55 and MLE of the birth-death prior -28977.37; BF = 9.82).

Diversification rates

All diversification analyses were conducted on the posterior trees that resulted from the dating analyses with the Yule prior as the branching process. Diversification analyses using only the MCC tree reconstructed with the birth-death prior indicated no difference from analyses of the MCC reconstructed with the Yule prior (results not shown).

The palaeo-environmental models of Condamine et al.[5] are directly comparable since they rely on the same likelihood framework. To show the relative performance of one model over another, we also computed the Akaike weights (the relative likelihood of the model compared to others) for all these models. The best model is the one with constant speciation and varying extinction (Fig. 3 of the main text). Within this model, the palaeo-environmental models have similar performances (none of them is significantly more supported than the other) and outperform the simple time-dependence model. This result suggests that past environmental changes may have impacted the diversification dynamics of birdwing butterflies.

Text S2. Expanded Materials and Methods

Taxon sampling

All species of birdwing butterflies are currently included in CITES and/or IUCN lists in order to protect them by regulating their commercial trade. Direct sampling in the wild is almost impossible and it is difficult to obtain specimens suitable for DNA extraction via alternative means. In this study, the total sampling includes 182 birdwing specimens for which half of them (85 specimens) came from quarantine interception at the Canadian border. This material was sent to Dr. Donald Lafontaine, of the Canadian National Collection of Insects, Arachnids and Nematodes (CNC) of Agriculture and Agri-Food Canada. These specimens were then made available to Dr. Felix Sperling on an indefinite long-term loan so that they could be preserved as frozen genetic material for future research at the Strickland Museum of Entomology, University of Alberta, Canada. Despite intense efforts, DNA sequencing was arduous and few genes were amplified (Supplementary Table S4). We considered next-generation approaches to sequence some of our specimens ("museomics"[6]), but this represented funding challenges, timelines and technical complexities (e.g. working with dried specimens that have degraded DNA) that made these approaches impossible to implement within the framework of our project. Finally, the rest of the sampling (97 specimens) came from previous studies, and in particular for several rare species (e.g. *Ornithoptera alexandrae, O. meridionalis, O. paradisea*, *O. victoriae*, *Troides andromache*, *T. miranda*). Thus we used DNA sequence data from GenBank that has been already published in earlier studies (e.g.[2-4]).

Ornithoptera allotei (Rothschild, 1914) was not considered as a valid species because it is a natural hybrid between *O. priamus urvilleanus* and *O. victoriae* in the Solomon Islands[7]. For the same reason, *Ornithoptera akakeae* (Kobayashi & Koiwaya, 1978) was also

not treated as a valid species (it is an hybrid between *O. priamus aureus* × *O. tithonus misresiana*)[7].

Molecular dataset

The molecular dataset consisted of DNA sequence for three mitochondrial gene fragments: cytochrome c oxidase subunit I (COI, 1,550 aligned nucleotides), NADH dehydrogenase 5 (ND5, 907 aligned nucleotides), 16S rRNA (16S, 511 aligned nucleotides), and two nuclear gene fragments: elongation factor 1 alpha (EF-1α, 995 aligned nucleotides) and wingless (Wg, 425 aligned nucleotides). For newly sequenced specimens, total genomic DNA was extracted from legs using the Qiagen® DNeasy tissue kit (Qiagen, Venlo, Netherlands). Polymerase chain reactions were performed using the following program: an initial 2 min denaturation step at 94°C, followed by 35 cycles of 1 min at 94°C, 1 min at 47-57°C (depending on primer combinations) and 1 min at 72°C and then a 7 min final extension step at 72°C. Nucleotide sequences of the primers used are standard and have been previously described and summarized[8].

Alignment and partitioning strategies

Sequences were assembled into contiguous arrays using Geneious Pro 5.1.7 (www.geneious.com). The sequences were aligned with those retrieved from a previous study using MAFFT 7[9] with default settings. Reading frames of protein-coding genes were checked under Mesquite 2.75 (www.mesquiteproject. org).

To determine the best-fit partitioning scheme of molecular evolution for our dataset, we used PartitionFinder 1.1.1[10]. In order to assess species monophyly independently with each gene (see above), we first estimated the best partitioning strategy for each individual gene of a given specimen-level dataset (e.g. 124 specimens for the COI gene). Second we

repeated this procedure for the species-level dataset, i.e. including one specimen per species. The partitioning scheme was then used for BEAST dating analyses. For PartitionFinder analyses branch lengths were *unlinked* to allow the program to estimate them independently for each subset. The best model, among the ones available in MrBayes 3.2.2[11], was searched for under the *greedy* search algorithm based on the Bayesian Information Criterion (BIC) model metric. Based on BIC scores for each partition, GTR+I+G or HKY+G model were the best-scoring models, followed closely by the SYM+I+G and HKY+I+G models. We used the partitioning scheme suggested by PartitionFinder but instead of using a specific model for each partition we used the *mixed* model option. The latter allows to sample across the substitution model space in the Bayesian Markov Chains Monte Carlo analysis itself ('rj-MCMC'), removing the need for a priori model testing[12].

Phylogenetic analyses and species monophyly

To reconstruct independent gene trees we used the four genes for which we have a comprehensive taxon sampling (i.e. 124 specimens for the COI, 92 for the ND5, 61 for the 16S, and 67 for the EF-1α). For each gene, Bayesian inferences were performed with MrBayes 3.2.2 and the partitioning scheme inferred using PartitionFinder (see above). Analyses consisted of four rj-MCMC running for 2.10^7 generations with sampling every 2,000 generations and the first 25% discarded as burn-in. As outgroups we used species from the two genera - *Cressida* and *Pharmacophagus* - that are most closely related to birdwing butterflies[13]. Convergence of runs was assessed by checking the potential scale reduction factor (PSRF) values of each parameter in MrBayes and the Effective Sample Size (ESS) values of each parameter in Tracer 1.6[14]. Value of PSRF close to 1.0 and ESS $\geq$ 200 were considered as good indicator of convergence. Nodes recovered with posterior probabilities (PP) $\geq$ 0.95 were considered to be strongly supported.

Calibrations and molecular dating

For dating analyses, we used a subset of the concatenated dataset comprising only one specimen for each species (as defined by the specimen-level analyses). The applicability of a molecular clock was investigated for each node using PATHd8[15]. The hypothesis of a molecular clock was not statistically supported for the majority of the nodes ($P < 0.05$; clock tests rejected for 42 of the 64 nodes). Therefore a dating approach that accounted for rate variation across lineages was used[16].

Species-level phylogenetic and dating analyses were carried out with the program BEAST 1.8[16]. The .xml file for the BEAST analysis was created under the BEAUti interface with the following non-default settings and priors: *Site Models* were set for each partition according to the best-fit partitioning strategy as recovered by PartitionFinder (see above), *Clock Model* was set to an uncorrelated lognormal relaxed clock for each partition, *Tree Model* was set to a Yule or birth-death process of speciation (for comparison purpose), and *MCMC parameters* were set to 5.10^7 generations with sampling every 5,000 generations and the first 25% discarded as burn-in.

Six calibrations points were used to time-calibrate the tree. We relied on the fossil-calibrated tree of Papilionidae[17] to select the calibrations for strongly supported nodes that were recovered in several studies, and whose monophyly was supported by both molecular and morphological data[8,17]. We enforced the monophyly of these nodes so that BEAST did not re-estimate the whole topology. The following calibration points were used in our study:

1. the crown of Papilioninae was set between 40.61 and 57.39 Ma (with a starting value at 47.02 Ma);

2. the crown of Troidini was set between 32.45 and 46.92 Ma;

3. the crown of the genus *Battus* was set between 17.22 and 29.29 Ma;

4. the crown of the subtribe Troidina was set between 26.46 and 38.91 Ma;

5. the divergence between the genera *Euryades* and *Parides* was set between 22.77 and 34.22 Ma;

6. the crown of the genus *Parides* was set between 18.56 and 28.45 Ma.

No internal calibrations were used for birdwing butterflies because we wanted to re-estimate the age and credibility intervals without prior assumptions on their ages, and let BEAST estimate the phylogenetic relationships within the clade. *Graphium agamemnon* was used to root the phylogenetic tree as the tribe Leptocircini is closest to Troidini[17]. All calibrations were set with uniform distribution enforcing hard bounds for minimum and maximum ages as provided by the fossil-calibrated tree of Papilionidae[17] (minimum and maximum values correspond to the 95% height posterior density of ages from the study of Condamine et al.[17]). A random starting tree was reconstructed by fitting the corresponding calibration points.

Convergence and performance of the MCMC were evaluated using Tracer based on the ESS values for each parameter. A maximum-clade credibility tree was computed, with median age and 95% height posterior density (HPD) calculated using TreeAnnotator.

Model selection: Yule model versus birth-death process

In Bayesian analyses, harmonic mean estimates (HME) have traditionally been used to compare models. New approaches have improved model selection for phylogenetics, such as path sampling (PS,[18]) and stepping-stone sampling (SS,[19]), which allow a better estimation of the marginal likelihood estimate (MLE) of a given model[20]. Here we used SS because it outperforms both PS and HME for direct Bayes factor (BF) calculations[21,22]. Stepping-stone sampling is implemented in BEAST and, following the recommendation of Baele et al.[22], we performed MLE using SS with a chain length of 1,000,000 iterations and a number of path

steps of 150. We then determined the power posteriors according to evenly spaced quantiles of a Beta (0.3, 1.0) distribution, thereby estimating more power posteriors close to the prior. The MLE allows direct BF calculation that we used to make pairwise comparisons between the Yule model and the birth-death process for a given calibration strategy. We considered BF values > 6.0 to favour one model over another[23].

Estimation of biogeographical history

Ancestral areas estimation was performed using the R-package "BioGeography with Bayesian (and likelihood) Evolutionary Analysis of RangeS" (BioGeoBEARS)[24]. Analyses were performed using the BEAST time-calibrated phylogenies (with outgroups removed).

Biogeographical models require the specification of operational discrete areas, and the biogeographical ranges of species consist of presence and absence in each area. These operational areas are often delimited by geological or oceanic features (which may have acted as barriers to dispersal). The distribution of birdwing butterflies ranges from western India to Australia (Fig. 1). We divided these two biomes into smaller biogeographic identities to get more resolution in the inference of the ancestral area of origin. To do so, we used tectonic reconstructions (e.g.[25]). The model comprised nine component areas: (A) India and Sri Lanka; (B) Malaysian Archipelago; (C) Philippines; (D) Sulawesi; (E) Lesser Sunda Islands; (F) Halmahera and Northern Moluccas; (G) New Guinea; (H); Solomon Islands and (I) Australia. The adjacency matrix was then designed by taking into account Earth's geological history and the biological plausibility of combined ranges. All ranges larger than three areas in size that were not subsets of observed species ranges were discarded. Distributional data were compiled from monographs and reviews (e.g.[1]) and species ranges were defined by presence–absence data and by excluding marginal distributions.

We did not use smaller geographical areas for several reasons. The complexity of the geological history of the IAA region makes difficult to reliably estimate the past distribution of land and sea at a given point in time. For instance many uncertainties remain about the appearance of important geological areas like Halmahera or Sulawesi. Furthermore the build-up of the Philippines and New Guinea represents one of the most challenging palaeogeographical issues to date. It is also reasonable to limit the number of areas to some large, discrete areas, since these are most likely to be relatively "conserved" (i.e. change rarely) over evolutionary time; and we have to compromise with the computational limitations. Moreover our goal was not to reconstruct a precise history of all biogeographical events but to investigate whether the clade appeared in the Sahul shelf or in the Sunda shelf (as predicted by A.R. Wallace), and also to test evolutionary hypotheses on the biogeographical processes that shaped the current biodiversity pattern. For the latter, we were particularly interested in assessing the relative importance of vicariance and dispersal processes in explaining the range patterns observed on the phylogeny. Regarding dispersal patterns we teased apart dispersal leading to range expansion from jump dispersal leading to founder-event speciation[24].

BioGeoBEARS is particularly adapted to examine competing hypotheses in island-dwelling clades[24]. Here we reconstructed the biogeographical history of birdwing butterflies using six models: DEC, DEC+J, DIVA-like, DIVA-like+J, BayArea-like, and BayArea-like+J. The BioGeoBEARS Dispersal-Extinction-Cladogenesis (DEC) is identical to the widely used model of Ree and Smith[26], but here it is implemented in the R environment. The BioGeoBEARS “DIVA-like” model is not identical to Ronquist[27]’s parsimony DIVA. It is a likelihood interpretation of DIVA, which only allows the processes implement under DIVA (see Fig. 4 in[28]). The BioGeoBEARS “BayArea-like” model is not identical to the complete Bayesian model implemented in the BayArea program of Landis et al.[29]. Instead, it is a

simplified likelihood interpretation of the BayArea model that replicates BayArea's cladogenesis assumption (which postulates that nothing in particular happens at cladogenesis). The purpose of having a BayArea-like model was to test the potential importance of the cladogenesis model on particular datasets.

The three pairs of models were compared to see whether the inclusion of founder-event speciation (parameter J) significantly improved likelihood using AICc. We also used AICc and LRT to select the best-fit model out of the six that were implemented. The corresponding best-fit model was then used to make the final biogeographical reconstruction shown in Fig. 2 and Supplementary Fig. S3.

Diversification analyses

Lineages-through-time.

To visualize the tempo and mode of diversification of the group, we first reconstructed lineages-through-time (LTT) plots for the whole tree and for the genera *Ornithoptera* and *Troides* (Supplementary Fig. S4).

Trait-dependent diversification.

If there are *a priori* reasons to believe that features of a species (e.g. ecological or morphological traits, geographical locations) influence diversification, it is relevant to use trait-dependent diversification models that concurrently model trait evolution and its impact on diversification[30,31]. In those models, species are characterized by an evolving trait, and follow a birth-death process in which speciation and extinction rates depend on the value of the trait at that time. Several models derived from the Binary State Speciation and Extinction model (BiSSE,[31]) have been used in various biogeographic contexts, e.g. the Geographic

State Speciation and Extinction (GeoSSE,[32]). However, those models have rarely been applied in island biogeography.

Here we used 500 trees randomly selected from the Bayesian posterior distribution of dating analyses and we combined them with distributional data categorized in two traits: species occurring extra-Wallacea (trait A), and species occurring within Wallacea (trait B) [species can be widespread, i.e. occur within the two regions, and are coded AB]. We then fitted 12 GeoSSE diversification models to estimate the speciation rate for trait A (sA) and B (sB), the allopatric speciation rate between the two regions (sAB), the extinction rate (xA / xB), and the transition rate between trait A/B and B/A (denoted qAB / qBA).

Models are as follows (see also Supplementary Table S3):

(i) sA = sB = sAB, xA = xB, dAB = dBA;

(ii) sA ≠ sB ≠ sAB, xA = xB, dAB = dBA;

(iii) sA ≠ sB, sAB = 0, xA = xB, dAB = dBA;

(iv) sA = sB ≠ sAB, xA = xB, dAB = dBA;

(v) sA = sB ≠ sAB, xA ≠ xB, dAB = dBA;

(vi) sA = sB ≠ sAB, xA = xB, dAB ≠ dBA;

(vii) sA = sB = sAB, xA ≠ xB, dAB = dBA;

(viii) sA = sB = sAB, xA = xB, dAB ≠ dBA;

(ix) sA ≠ sB ≠ sAB, xA ≠ xB, dAB = dBA;

(x) sA ≠ sB ≠ sAB, xA = xB, dAB ≠ dBA;

(xi) sA = sB = sAB, xA ≠ xB, dAB ≠ dBA;

(xii) sA ≠ sB ≠ sAB, xA ≠ xB, dAB ≠ dBA.

Analyses were performed using the R-package *diversitree* 0.7-6[33] using the functions *make.geosse* to construct the likelihood functions for each model based on the data, and the functions *constrain* and *find.mle* to apply different diversification scenarios as detailed above.

For each GeoSSE model, we computed the AICc. We checked support for the selected model against all models nested within it using the Likelihood Ratio Test (LRT, significant at $P < 0.05$). The scenario supported by LRT and with the lowest AICc was considered the best. If the model with the lowest AICc was not supported by LRT, the model with the least parameters was considered the best. Net diversification rates ($r=\lambda - \mu$) were computed from speciation and extinction rate estimates. Finally we used the maximum clade credibility (MCC) tree and a MCMC approach for the best model to examine the credibility intervals of the parameter estimates. We used an exponential prior 1/(2r) to be as conservative as possible[33] and started the chain with the parameters obtained by maximum likelihood. We ran 20,000 steps of MCMC and applied a burn-in of 2,000 steps.

Palaeo-environment-dependent diversification.

To test the effect that past environment had on the diversification of birdwings, we used a birth-model derived from the one of Morlon et al.[34] that allows speciation and extinction rates to vary according to a past environmental variable itself varying through time[5], such as past variations of temperature[35] or past fluctuations of sea level[36].

Like the Morlon et al.[33]'s approach, we designed three models to test: (i) BVARDCST, speciation rate varies exponentially with temperature and extinction rate is constant; (ii) BCSTDVAR, speciation rate is constant and extinction rate varies exponentially with temperature; and (iii) BVARDVAR, speciation and extinction rates both vary exponentially with temperature. We also repeated these three models with linear dependence with temperature. The tree was analyzed as a whole using this approach and the four missing species were taken into account (stating f=34/38).

These models rely on a past environmental variable; as such any user needs to inform the model with data on how the environment varied through time. For temperature and sea

levels, such data exist and are compiled into tables. Here we relied on the well-known Cenozoic temperatures published by Zachos et al.[35], and on the Phanerozoic sea levels published by Miller et al.[36]. Because there is fewer consensus on sea level curves than temperature curves, several analyses were performed with different sea level curves. We alternatively used the sea level data inferred by Haq[37], Miller et al.[36], and Kominz et al.[38]. Because very similar results (results not shown) were inferred using the different curves, we choose to only present the results based on the most recent sea level curve[38] in the main text.

The R package *pspline* (http://cran.r-project.org/web/packages/pspline/pspline.pdf) was used to reconstruct smooth lines of palaeo-data for each environmental variable. For instance, a smooth line is introduced in the birth-death model to represent the palaeo-environment through time, and at each point in time the model refers to this smooth line to give the temperature value of the temperature. Given the dated phylogeny, the model then estimates speciation and extinction rates as a function of this value.

For each diversification model, we computed the AICc. We also used Likelihood Ratio Tests (LRT, significant at $P < 0.05$) to estimate support for a particular model when compared to the remaining models. The scenario supported by LRT and with the lowest AICc was considered as the best. If the model with the lowest AICc was not supported by LRT, the model with the least parameters was considered as the best.

Supplementary references

1. Haugum, J. & Low, A. M. *A monograph of the birdwing butterflies*. *The systematics of* Ornithoptera*,* Troides *and related genera* (Denmark, Scandinavian Science Press, 1978-1985).
2. Morinaka, S. et al. Molecular phylogeny of birdwing butterflies based on the representatives in most genera of the tribe Troidini (Lep., Papilionidae). *Entomol. Sci.* **2**, 347-358 (1999).
3. Kondo, K., Shinkawa, T. & Matsuka, H. Molecular systematics of birdwing butterflies (Papilionidae) inferred from mitochondrial ND5 gene. *J. Lepido. Soc.* **57**, 17-24 (2003).
4. Braby, M. F., Trueman, J. W. H. & Eastwood, R. When and where did troidine butterflies (Lepidoptera: Papilionidae) evolve? Phylogenetic and biogeographic evidence suggests an origin in remnant Gondwana in the Late Cretaceous. *Invert. Syst.* **19**, 113–143 (2005).
5. Condamine, F. L., Rolland, J. & Morlon H. Macroevolutionary perspectives to environmental change. *Ecol. Lett.* **16**, 72-75 (2013).
6. Guschanski, K. et al. Next-generation museomics disentangles one of the largest primate radiations. *Syst. Biol.* **62**, 539-554 (2013).
7. Hancock, D. L. Notes on the phylogeny and biogeography of *Ornithoptera* Boisduval (Lepidoptera: Papilionidae). *Tyô to Ga* **42**, 17–36 (1991).
8. Simonsen, T. J. et al. Phylogenetics and divergence times of Papilioninae (Lepidoptera) with special reference to the enigmatic genera *Teinopalpus* and *Meandrusa*. *Cladistics* **27**, 113–137 (2011).
9. Katoh, K. & Standley, D. M. MAFFT multiple sequence alignment software version 7: improvements in performance and usability. *Mol. Biol. Evol.* **30**, 772-780 (2013).

10. Lanfear, R., Calcott, B., Ho, S. Y. W. & Guindon, S. PartitionFinder: combined selection of partitioning schemes and substitution models for phylogenetic analyses. *Mol. Biol. Evol.* **29**, 1695–1701 (2012).

11. Ronquist, F. et al. MrBayes 3.2: efficient Bayesian phylogenetic inference and model choice across a large model space. *Syst. Biol.* **61**, 539–542 (2012).

12. Huelsenbeck, J. P., Larget, B. & Alfaro, M. E. Bayesian phylogenetic model selection using reversible jump Markov chain Monte Carlo. *Mol. Biol. Evol.* **21**, 1123–1133 (2004).

13. Condamine, F. L., Sperling, F. A. H. & Kergoat, G. J. Global biogeographical pattern of swallowtail diversification demonstrates alternative colonization routes in the Northern and Southern hemispheres. *J. Biogeogr.* **40**, 9-23 (2013).

14. Rambaut, A., Suchard, M. A., Xie, D. & Drummond, A. J. Tracer v1.6, Available from http://beast.bio.ed.ac.uk/Tracer (2014).

15. Britton, T., Anderson, C. L., Jacquet, D., Lundqvist, S. & Bremer, K. Estimating divergence times in large phylogenetic trees. *Syst. Biol.* **56**, 741–752 (2007).

16. Drummond, A., Suchard, M. A., Xie, D. & Rambaut, A. Bayesian phylogenetics with BEAUti and the BEAST 1.7. *Mol. Biol. Evol.* **29**, 1969-1973 (2012).

17. Condamine, F. L., Sperling, F. A., Wahlberg, N., Rasplus, J. Y. & Kergoat, G. J. What causes latitudinal gradients in species diversity? Evolutionary processes and ecological constraints on swallowtail biodiversity. *Ecol. Lett.* **15**, 267–277 (2012).

18. Lartillot, N. & Philippe, H. Computing Bayes factors using thermodynamic integration. *Syst. Biol.* **55**, 195–207 (2006).

19. Xie, W., Lewis, P. O., Fan, Y., Kuo, L. & Chen, M. H. Improving marginal likelihood estimation for Bayesian phylogenetic model selection. *Syst. Biol.* **60**, 150–160 (2011).

20. Baele, G. et al. Improving the accuracy of demographic and molecular clock model comparison while accommodating phylogenetic uncertainty. *Mol. Biol. Evol.* **29**, 2157–2167 (2012).

21. Baele, G., Li, W. L. S., Drummond, A. J., Suchard, M. A. & Lemey, P. Accurate model selection of relaxed molecular clocks in Bayesian phylogenetics. *Mol. Biol. Evol.* **30**, 239–243 (2013).

22. Baele, G., Lemey, P. & Vansteelandt, S. Make the most of your samples: Bayes factor estimators for high-dimensional models of sequence evolution. *BMC Bioinf.* **14**, 85 (2013).

23. Kass, R. E. & Raftery, A. E. Bayes factors. *J. Am. Stat. Assoc.* **90**, 773–795 (1995).

24. Matzke, N. J. Model selection in historical biogeography reveals that founder-event speciation is a crucial process in island clades. *Syst. Biol*. **63**, 951-970 (2014).

25. Hall, R. Late Jurassic–Cenozoic reconstructions of the Indonesian region and the Indian Ocean. *Tectonophys*. **570**, 1–41 (2012).

26. Ree, R. H & Smith, S. A. Maximum-likelihood inference of geographic range evolution by dispersal, local extinction, and cladogenesis. *Syst. Biol.* **57**, 4–14 (2008).

27. Ronquist, F. Dispersal-vicariance analysis: A new approach to the quantification of historical biogeography. *Syst. Biol*. **46**, 195-203 (1997).

28. Ronquist, F. & Sanmartín, I. Phylogenetic methods in biogeography. *Ann. Rev. Ecol., Evol., Syst.* **42**, 441-464 (2011).

29. Landis, M., Matzke, N. J., Moore, B. R. & Huelsenbeck, J. P. Bayesian analysis of biogeography when the number of areas is large. *Syst. Biol.* **62**, 789-804 (2013).

30. Maddison, W. P. Confounding asymmetries in evolutionary diversification and character change. *Evolution* **60**, 1743–1746 (2006).

31. Maddison, W. P, Midford, P. E., Otto, S. P. Estimating a binary character's effect on speciation and extinction. *Syst. Biol.* **56**, 701–710 (2007).

32. Goldberg, E. E, Lancaster, L. T & Ree, R. H. Phylogenetic inference of reciprocal effects between geographic range evolution and diversification. *Syst. Biol.* **60**, 451–465 (2011).

33. FitzJohn, R. G. Diversitree: comparative phylogenetic analyses of diversification in R. *Methods Ecol. Evol.* **3**, 1084–1092 (2012).

34. Morlon, H, Parsons, T. L. & Plotkin, J. Reconciling molecular phylogenies with the fossil record. *Proc. Natl Acad, Sci. USA* **108**, 16327-16332 (2011).

35. Zachos, J. C., Dickens, G. R. & Zeebe, R. E. An early Cenozoic perspective on greenhouse warming and carbon-cycle dynamics. *Nature* **451**, 279–283 (2008).

36. Miller, et al. The Phanerozoic record of global sea-level change. *Science* **312**, 1293–1298 (2005).

37. Haq, B. U., Hardenbol, J. & Vail, P. R. Chronology of fluctuating sea levels since the Triassic. *Science* **235**, 1156-1167 (1987).

38. Kominz, et al. Late Cretaceous to Miocene sea-level estimates from the New Jersey and Delaware coastal plain coreholes: an error analysis. *Basin Res.* **20**, 211-226 (2008).

Table S1. Results of biogeographical analyses. df, degree of freedom; logL, log-likelihood; d, dispersal rate; e, extinction rate; j, jump dispersal (foun[illegible] event dispersal). Bold and red line indicates the best model of this approach. The ΔAICc compares pair of models (e.g. DEC vs. DEC+J) and the ωAICc compares all the models together to select the best one.

**Models**	**df**	**logL**	**AICc**	**ΔAICc**	**ωAICc**	**d**	**e**	**j**
DEC	2	-99.47045	203.328	14.923	0.0005	0.0109	1.01E-02	0
**DEC+J**	**3**	**-90.8027**	**188.405**	**0**	**0.782**	**0.0055**	**1.00E-12**	**0.0432**
DIVA-like	2	-101.54626	207.408	16.435	0.0005	0.0143	1.36E-02	0
DIVA-like+J	3	-92.08654	190.973	0	0.216	0.0062	1.00E-12	0.0413
BayArea	2	-120.68135	245.75	44.657	0	0.0178	1.32E-01	0
BayArea+J	3	-97.14662	201.093	0	0.001	0.0043	4.13E-03	0.0640

Table S2. Results of diversity-dependence diversification analyses. df, degree of freedom; logL, log-likelihood; λ, speciation rate; μ, extinction rate a present; K, estimated carrying capacity. Bold and red lines indicate the best model of this approach for each clade. The ΔAICc and ωAICc are used to co all the models together to select the best one.

(*a*) All birdwing butterflies

Clade	Species richness	Model	df	logL	AICc	ΔAICc	ωAICc	λ	μ	K
Birdwings (all three genera)	34 species sampled out of 38 described	DDL	2	-88.572	181.53	4.625	0.086	0.196	-	257.54
		DDL+E	3	-92.315	191.43	14.524	0	0.1105	0	Inf
		DDX+E	3	-88.621	184.04	7.135	0.024	0.233	0.0512	Inf
		DD+EL	3	-88.566	183.93	7.026	0.026	0.2026	0.0024	112.46
		**DD+EX**	**3**	**-85.053**	**176.91**	**0**	**0.863**	**0.2337**	**≈0**	**40.07**

(*b*) *Troides*

Clade	Species richness	Model	df	logL	AICc	ΔAICc	ωAICc	λ	μ	K
Troides	18 species sampled out of 22 described	DDL	2	-39.501	83.802	3.024	0.167	0.4134	-	29.51
		DDL+E	3	-39.496	86.707	5.929	0.039	0.4388	0.0229	28.06
		DDX+E	3	-40.153	88.02	7.242	0.02	0.4238	0.0023	Inf
		DD+EL	3	-40.415	88.544	7.766	0.016	0.2452	≈0	Inf
		**DD+EX**	**3**	**-36.532**	**80.778**	**0**	**0.758**	**0.3311**	**≈0**	**23.7**

(*c*) ***Ornithoptera***

Clade	Species richness	Model	df	logL	AICc	ΔAICc	ωAICc	λ	μ	K
Ornithoptera	all 14 species sampled	DDL	2	-39.311	83.712	17.37	0	0.05	-	580639
		DDL+E	3	-30.267	68.935	2.593	0.192	0.9917	0.1617	12.55
		DDX+E	3	-31.074	70.548	4.206	0.085	2.7256	0.1118	17.42
		DD+EL	3	-32.403	73.207	6.865	0.023	0.2704	≈0	20.82
		**DD+EX**	**3**	**-28.971**	**66.342**	**0**	**0.7**	**0.2693**	**≈0**	**14.42**

Table S3. Results of trait-dependence (GeoSSE) diversification analyses. df, degree of freedom; logL, log-likelihood; sA/sB, speciation rate for trait Wallacea)/B (Wallacea); sAB, allopatric speciation rate; xA/B, extinction rate for trait A (non-Wallacea)/B (Wallacea); dAB, transition rate between sta and B (and conversely for dBA). Bold and red line indicates the best model of this approach. Confidence intervals are derived from the distribution of maximum likelihood parameter values on a random sample of dated trees (obtained from the BEAST dating analysis). The ΔAICc and ωAICc are used compare all the models together to select the best one.

Model	df	logL	AICc	ΔAICc	ωAICc	sA	sB	sAB	xA	xB	dAB	dBA
sA = sB = sAB, xA = xB, dAB = dBA	3	-123.920 ±0.231	254.639 ±0.462	3.234	0.074	0.1652 ±0.0020			0.0709 ±0.0044		0.0495 ±0.0011	
sA ≠ sB ≠ sAB, xA = xB, dAB = dBA	5	-123.401 ±0.227	258.945 ±0.455	7.540	0.009	0.1752 ±0.0032	0.1663 ±0.0022	0.2756 ±0.0112	0.0889 ±0.0051		0.0549 ±0.0013	
sA ≠ sB, sAB = 0, xA = xB, dAB = dBA	4	-125.197 ±0.273	259.773 ±0.546	8.368	0.006	0.2560 ±0.0036	0.1975 ±0.0015	0	0.2081 ±0.0052		0.0658 ±0.0014	
sA = sB ≠ sAB, xA = xB, dAB = dBA	4	-123.653 ±0.226	256.685 ±0.453	5.280	0.0266	0.1559 ±0.0020		0.2550 ±0.0107	0.0557 ±0.0041		0.0487 ±0.0010	
sA = sB ≠ sAB, xA ≠ xB, dAB = dBA	5	-122.229 ±0.211	256.601 ±0.423	5.196	0.0277	0.1743 ±0.0017		0.3344 ±0.0164	0.0865 ±0.0023	0.2321 ±0.0042	0.0828 ±0.0017	
sA = sB ≠ sAB, xA = xB, dAB ≠ dBA	5	-120.682 ±0.196	253.508 ±0.391	2.102	0.13	0.1768 ±0.0023		0.3965 ±0.0138	0.1399 ±0.0040		0.0212 ±0.0005	0.2458 ±0.0041
sA = sB = sAB, xA ≠ xB, dAB = dBA	4	-122.519 ±0.216	254.417 ±0.431	3.011	0.083	0.1860 ±0.0017			0.1003 ±0.0026	0.2438 ±0.0047	0.0810 ±0.0014	
**sA = sB = sAB, xA = xB, dAB ≠ dBA**	**4**	**-121.013 ±0.200**	**251.405 ±0.401**	**0**	**0.372**	**0.2004 ±0.0018**			**0.1767 ±0.0033**		**0.0216 ±0.0004**	**0.2625 ±0.0042**
sA ≠ sB ≠ sAB, xA ≠ xB, dAB = dBA	6	-120.895 ±0.211	256.900 ±0.421	5.495	0.024	0.1186 ±0.0022	0.4150 ±0.0079	0.3179 ±0.0196	0.0702 ±0.0018	0.4868 ±0.0113	0.1237 ±0.0036	
sA ≠ sB ≠ sAB, xA = xB, dAB ≠ dBA	6	-119.493 ±0.196	254.096 ±0.391	2.691	0.097	0.1443 ±0.0020	0.2845 ±0.0031	0.3583 ±0.0119	0.2427 ±0.0046		0.0008 ±0.0003	0.3769 ±0.0068
sA = sB = sAB, xA ≠ xB, dAB ≠ dBA	5	-120.720 ±0.197	253.582 ±0.393	2.177	0.125	0.2111 ±0.0022			0.2301 ±0.0058	0.1750 ±0.0031	0.0083 ±0.0008	0.3234 ±0.0074
sA ≠ sB ≠ sAB, xA ≠ xB, dAB ≠ dBA	7	-119.181 ±0.199	256.670 ±0.397	5.265	0.027	0.1184 ±0.0024	0.3711 ±0.0073	0.2796 ±0.0140	0.2037 ±0.0053	0.3603 ±0.0095	0.0172 ±0.0013	0.3227 ±0.0078

Table S4. Results of palaeo-environmental-dependence (a, temperature and b, sea level) diversification analyses. df, degree of freedom; logL, log likelihood; λ, speciation rate; α, rate of variation of the speciation according to the palaeo-environmental variable; μ, extinction rate; β, rate of variation [illegible] extinction according to the palaeo-environmental variable. Bold and red lines indicate the best model of each approach. Confidence intervals are derive[illegible] the distribution of maximum likelihood parameter values on a random sample of dated trees (obtained from the BEAST dating analysis). The ΔAICc an[illegible] ωAICc are used to compare all the models together to select the best one.

(*a*)

**Models**	**Mode of dependence**	**df**	**logL**	**AICc**	**ΔAICc**	**ωAICc**	**λ**	**α**	**μ**	**β**
λTemp. and μ constant	exponential	3	-92.338 ±0.191	191.476 ±0.382	2.574	0.175	0.2295 ±0.0024	-0.0632 ±0.0017	0.0293 ±0.0018	-
**λ constant and μTemp.**	**exponential**	**3**	**-91.051 ±0.192**	**188.902 ±0.383**	**0**	**0.633**	**0.1846 ±0.0013**	**-**	**0.0013 ±0.0003**	**0.9110 ±0.0199**
λTemp. and μTemp.	exponential	4	-90.953 ±0.193	191.286 ±0.387	2.385	0.192	0.1477 ±0.0028	0.1089 ±0.0088	0.0178 ±0.0019	0.5539 ±0.0170

(*b*)

**Models**	**Mode of dependence**	**df**	**logL**	**AICc**	**ΔAICc**	**ωAICc**	**λ**	**α**	**μ**	**β**
λSeaLevel and μ constant	exponential	3	-92.356 ±0.172	191.511 ±0.343	2.422	0.177	0.2610 ±0.0040	0.0041 ±0.0003	0.1501 ±0.0044	-
**λ constant and μSeaLevel**	**exponential**	**3**	**-91.145 ±0.191**	**189.089 ±0.383**	**0**	**0.594**	**0.1853 ±0.0011**	**-**	**0.0691 ±0.0031**	**0.1652 ±0.0061**
λSeaLevel and μSeaLevel	exponential	4	-90.810 ±0.192	190.999 ±0.384	1.909	0.229	0.2984 ±0.0155	0.0085 ±0.0007	0.2108 ±0.0182	0.1201 ±0.0054

Table S5. Taxon sampling and GenBank accession numbers. MG, morphological group *sensu* Haugun and Low[1]

Sample ID	MG	Species	Author	Country	Locality	COI	ND5	16S	Wg
**Not sampled**	haliphron species-group	***Troides darsius***	**(Gray 1853)**	**Sri Lanka**	**-**	**-**	**-**	**-**	**-**
FLC_00257		*Troides vandepolli vandepolli*	(Snellen 1890)	Indonesia	West Java, Mt. Wayang	SEQ	SEQ	SEQ	SEQ
FLC_00258		*Troides vandepolli vandepolli*	(Snellen 1890)	Indonesia	West Java, Mt. Wayang	SEQ	SEQ	SEQ	SEQ
FLC_00259		*Troides criton criton (f. androgyna)*	(Felder & Felder 1860)	Indonesia	Halmahera Island	SEQ	SEQ	SEQ	SEQ
FLC_00260		*Troides criton critonides*	(Fruhstorfer 1903)	Indonesia	North Moluccas, Obi Island	SEQ	SEQ	SEQ	SEQ
FLC_00261		*Troides criton criton*	(Felder & Felder 1860)	Indonesia	Moluccas, Halmahera Island	SEQ	SEQ	SEQ	SEQ
FLC_00262		*Troides criton criton*	(Felder & Felder 1860)	Indonesia	Moluccas, Halmahera Island	SEQ	SEQ	SEQ	SEQ
EC-063		*Troides criton criton*	(Felder & Felder 1860)	Indonesia	-	EF514410	-	-	-
EC-071		*Troides criton critonides*	(Fruhstorfer 1903)	Indonesia	-	EF514403	-	-	-
EC-084		*Troides riedeli*	(Kirsch 1885)	Indonesia	-	EF514423	-	-	-
EC-085		*Troides riedeli*	(Kirsch 1885)	Indonesia	-	EF514419	-	-	-
FLC_00264		*Troides plato*	(Wallace 1865)	Indonesia	Timor	SEQ	SEQ	SEQ	SEQ
FLC_00265		*Troides haliphron haliphron*	(Boisduval 1836)	Indonesia	South Sulawesi, Bantimurung	SEQ	SEQ	SEQ	SEQ
FLC_00266		*Troides haliphron haliphron*	(Boisduval 1836)	Indonesia	South Sulawesi, Bantimurung	SEQ	SEQ	SEQ	SEQ
FLC_00267		*Troides haliphron purabu*	Kobayashi 1987	Indonesia	Batuatas Island	SEQ	SEQ	SEQ	SEQ
FLC_00268		*Troides haliphron purabu*	Kobayashi 1987	Indonesia	Batuatas Island	SEQ	SEQ	SEQ	SEQ
FLC_00269		*Troides haliphron socrates*	(Staudinger 1891)	Indonesia	Wetar Island	SEQ	SEQ	SEQ	SEQ
FLC_00270		*Troides haliphron socrates*	(Staudinger 1891)	Indonesia	Wetar Island	SEQ	SEQ	SEQ	SEQ
FLC_00271		*Troides haliphron pallens*	(Oberthür 1897)	Indonesia	Selayar, Z-Sulawesi	SEQ	SEQ	SEQ	SEQ
FLC_00272		*Troides haliphron pallens*	(Oberthür 1897)	Indonesia	Selayar, Z-Sulawesi	SEQ	SEQ	SEQ	SEQ
FLC_00273		*Troides haliphron celebensis*	(Wallace 1865)	Indonesia	Maros, Z-Sulawesi	SEQ	SEQ	SEQ	SEQ

EC-081		*Troides haliphron haliphron*	(Boisduval 1836)	Indonesia	-	EF514425	-	-	-
EC-082		*Troides haliphron naias*	(Doherty 1891)	Indonesia	-	EF514415	-	-	-
EC-083		*Troides haliphron naias*	(Doherty 1891)	Indonesia	-	EF514404	-	-	-
38		*Troides haliphron haliphron*	(Boisduval 1836)	Indonesia	South Sulawesi, Bantimurung	-	AB044649	-	-
FLC_00274		*Troides staudingeri heptanonius*	(Fruhstorfer 1913)	Indonesia	Moluccas, Damar Island	SEQ	SEQ	SEQ	SEQ
FLC_00275		*Troides staudingeri heptanonius*	(Fruhstorfer 1913)	Indonesia	Moluccas, Damar Island	SEQ	SEQ	SEQ	SEQ
FLC_00276		*Troides staudingeri iris*	(Röber 1888)	Indonesia	Moluccas, Moa Island	SEQ	SEQ	SEQ	SEQ
FLC_00277		*Troides staudingeri iris*	(Röber 1888)	Indonesia	Moluccas, Moa Island	SEQ	SEQ	SEQ	SEQ
FLC_00278		*Troides staudingeri iris*	(Röber 1888)	Indonesia	Moluccas, Moa Island	SEQ	SEQ	SEQ	SEQ
877		*Troides staudingeri iris*	(Röber 1888)	Indonesia	Moluccas, Lakor Island	-	AB044651	-	-
EC-072		*Troides staudingeri ssp.*	(Röber 1888)	Indonesia	-	EF514417	-	-	-
EC-074		*Troides staudingeri ssp.*	(Röber 1888)	Indonesia	-	EF514427	-	-	-
EC-075		*Troides staudingeri ssp.*	(Röber 1888)	Indonesia	-	EF514426	-	-	-
EC-076		*Troides staudingeri ssp.*	(Röber 1888)	Indonesia	-	EF514449	-	-	-
FLC_00280	**helena species-group**	*Troides helena helena*	(Linnaeus 1758)	Indonesia	Butar, East java	SEQ	SEQ	SEQ	SEQ
FLC_00281		*Troides helena helena*	(Linnaeus 1758)	Indonesia	Butar, East java	SEQ	SEQ	SEQ	SEQ
FLC_00282		*Troides helena helena*	(Linnaeus 1758)	Indonesia	West Java	SEQ	SEQ	SEQ	SEQ
FLC_00283		*Troides helena helena*	(Linnaeus 1758)	Indonesia	West Java	SEQ	SEQ	SEQ	SEQ
455		*Troides helena helena*	(Linnaeus 1758)	Indonesia	Bali Island	-	AB027594	-	-
TypeT2		*Troides helena helena*	(Linnaeus 1758)	Indonesia	Bali Island	-	AB084430	-	-
FLC_00284		*Troides helena antileuca*	(Rothschild 1908)	Indonesia	Kangean Island	SEQ	SEQ	SEQ	SEQ
FLC_00285		*Troides helena antileuca*	(Rothschild 1908)	Indonesia	Kangean Island	SEQ	SEQ	SEQ	SEQ
FLC_00286		*Troides helena antileuca*	(Rothschild 1908)	Indonesia	Kangean Island	SEQ	SEQ	SEQ	SEQ

FLC_00287		*Troides helena mannus*	(Fruhstorfer 1908)	Indonesia	Bali Island	SEQ	SEQ	SEQ	SEQ
FLC_00288		*Troides helena mannus*	(Fruhstorfer 1908)	Indonesia	Bali Island	SEQ	SEQ	SEQ	SEQ
FLC_00289		*Troides helena mannus*	(Fruhstorfer 1908)	Indonesia	Bali Island	SEQ	SEQ	SEQ	SEQ
FLC_00290		*Troides helena hephaestus*	(Felder 1865)	Indonesia	South Sulawesi, Bantimurung	SEQ	SEQ	SEQ	SEQ
FLC_00291		*Troides helena hephaestus*	(Felder 1865)	Indonesia	South Sulawesi, Bantimurung	SEQ	SEQ	SEQ	SEQ
FLC_00292		*Troides helena spp.*	(Linnaeus 1758)	Indonesia	Bali Island	SEQ	SEQ	SEQ	SEQ
FLC_00293		*Troides helena spp.*	(Linnaeus 1758)	Indonesia	Bali Island	SEQ	SEQ	SEQ	SEQ
FLC_00294		*Troides helena spp.*	(Linnaeus 1758)	Malaysia	Cameron Highlands	SEQ	SEQ	SEQ	SEQ
FLC_00295		*Troides helena cerberus*	(Felder & Felder 1865)	Indonesia	-	SEQ	SEQ	SEQ	SEQ
FS-b-974		*Troides helena cerberus*	(Felder & Felder 1865)	Malaysia	-	AF170878	-	DQ351084	AY569047
JJW0011		*Troides helena cerberus*	(Felder & Felder 1865)	Malaysia	Selangor, Batu, Gombak, PPLUM	KF226654	-	-	-
JJW0013		*Troides helena cerberus*	(Felder & Felder 1865)	Malaysia	Negeri Sembilan, Jelebu, Hutan Simpan Kenaboi, N9	KF226655	-	-	-
EC-058		*Troides helena cerberus*	(Felder & Felder 1865)	India	-	EF514456	-	-	-
EC-079		*Troides helena cerberus*	(Felder & Felder 1865)	Indonesia	-	EF514446	-	-	-
FLC_00296		*Troides helena sagittatus*	(Fruhstorfer 1896)	Indonesia	Lombok Island	SEQ	SEQ	SEQ	SEQ
FLC_00297		*Troides oblongomaculatus aff. papuensis*	(Wallace 1865)	Indonesia	Talaud Island	SEQ	SEQ	SEQ	SEQ
FLC_00298		*Troides oblongomaculatus aff. papuensis*	(Wallace 1865)	Indonesia	Talaud Island	SEQ	SEQ	SEQ	SEQ
FLC_00299		*Troides oblongomaculatus papuensis*	(Wallace 1865)	New Guinea	Manki Range, Aseki Subdistrict, Morobe Province	SEQ	SEQ	SEQ	SEQ
FLC_00300		*Troides oblongomaculatus papuensis*	(Wallace 1865)	New Guinea	Watut, Bulolo, Aseki Subdistrict, Morobe Province	SEQ	SEQ	SEQ	SEQ
FLC_00301		*Troides oblongomaculatus papuensis*	(Wallace 1865)	New Guinea	Watut, Bulolo, Aseki Subdistrict, Morobe Province	SEQ	SEQ	SEQ	SEQ
FLC_00302		*Troides oblongomaculatus oblongomaculatus*	(Goeze 1779)	Indonesia	Binongko Island (East Sulawesi)	SEQ	SEQ	SEQ	SEQ
FLC_00303		*Troides oblongomaculatus oblongomaculatus*	(Goeze 1779)	Indonesia	Binongko Island (East Sulawesi)	SEQ	SEQ	SEQ	SEQ
FLC_00304		*Troides oblongomaculatus oblongomaculatus*	(Goeze 1779)	Indonesia	Moluccas, Seram Island	SEQ	SEQ	SEQ	SEQ

FLC_00305		*Troides oblongomaculatus oblongomaculatus*	(Goeze 1779)	Indonesia	Moluccas, Seram Island	SEQ	SEQ	SEQ	SEQ
EC-059		*Troides oblongomaculatus bandensis*	(Pagenstecher 1904)	Indonesia	Moluccas, Banda Island	EF514451	-	-	-
EC-060		*Troides oblongomaculatus bandensis*	(Pagenstecher 1904)	Indonesia	Moluccas, Banda Island	EF514450	-	-	-
EC-061		*Troides oblongomaculatus oblongomaculatus*	(Goeze 1779)	Indonesia	-	EF514448	-	-	-
EC-062		*Troides oblongomaculatus bandensis*	(Pagenstecher 1904)	Indonesia	Moluccas, Banda Island	EF514453	-	-	-
EC-077	**aeacus species-group**	*Troides aeacus aeacus*	(Felder & Felder 1860)	India	-	EF514455	-	-	-
FLC_00306		*Troides aeacus szechwanus*	Okano & Okano 1983	China	Ya'an area, Sichuan	SEQ	SEQ	SEQ	SEQ
Pa428		*Troides aeacus formosanus*	(Rothschild 1899)	Taiwan	Zanhua	AB576585	-	-	-
FLC_00307		*Troides magellanus magellanus*	(Felder & Felder 1862)	Philippines	Gasan, Marindugue, Fillippijnen	SEQ	SEQ	SEQ	SEQ
FLC_00308		*Troides magellanus apoensis*	(Felder & Felder 1862)	Philippines	Bohol, Fillippijnen	SEQ	SEQ	SEQ	SEQ
**Not sampled**		***Troides minos***	**(Cramer 1779)**	**India**	**Western Ghats, South India**	**-**	**-**	**-**	**-**
FLC_00310		*Troides rhadamantus*	(Lucas 1835)	Philippines	-	SEQ	SEQ	SEQ	SEQ
FLC_00311		*Troides rhadamantus*	(Lucas 1835)	Philippines	-	SEQ	SEQ	SEQ	SEQ
**Not sampled**		***Troides dohertyi***	**(Rippon 1893)**	**Indonesia**	**Talaud Island**	**-**	**-**	**-**	**-**
**Not sampled**		***Troides plateni***	**(Staudinger 1888)**	**Philippines**	**Palawan, Balabac, Dumaran, Busuanga**	**-**	**-**	**-**	**-**
EC-002		*Troides prattorum*	(Joicey & Talbot 1922)	Indonesia	Moluccas, Buru Island	EF514421	-	-	-
510		*Troides prattorum*	(Joicey & Talbot 1922)	Indonesia	Moluccas, Buru Island	-	AB044650	-	-
FLC_00313	**amphrysus species-group**	*Troides amphrysus joane*	Parrott 1991	Indonesia	Bali Island	SEQ	SEQ	SEQ	SEQ
FLC_00314		*Troides amphrysus amphrysus*	(Cramer 1779)	Indonesia	Java	SEQ	SEQ	SEQ	SEQ
FLC_00315		*Troides amphrysus amphrysus*	(Cramer 1779)	Indonesia	Java	SEQ	SEQ	SEQ	SEQ
317		*Troides amphrysus amphrysus*	(Cramer 1779)	Indonesia	Java	-	AB027593	-	-
TypeT3		*Troides amphrysus euthydemus*	(Fruhstorfer 1913)	Indonesia	Sumatra	-	AB084431	-	-
JJW0012		*Troides amphrysus ruficolis*	(Butler 1879)	Malaysia	Negeri Sembilan, Berembun Forest Reserve	KF226653	-	-	-

EC-046		*Troides amphrysus ssp.*	(Cramer 1779)	Indonesia	-	EF514412	-	-	-
EC-005		*Troides andromache*	(Staudinger 1892)	Malaysia	Borneo	EF514437	-	-	-
EC-006		*Troides andromache*	(Staudinger 1892)	Malaysia	Borneo	EF514440	-	-	-
FLC_00317		*Troides cuneifera cuneifera*	(Oberthür 1879)	Indonesia	Java, Mt. Argopuro	SEQ	SEQ	SEQ	SEQ
FLC_00318		*Troides cuneifera cuneifera*	(Oberthür 1879)	Indonesia	Java, Mt. Argopuro	SEQ	SEQ	SEQ	SEQ
FLC_00319		*Troides cuneifera cuneifera*	(Oberthür 1879)	Indonesia	Java, Mt. Wayana	SEQ	SEQ	SEQ	SEQ
FLC_00320		*Troides cuneifera cuneifera*	(Oberthür 1879)	Indonesia	Java, Mt. Wayana	SEQ	SEQ	SEQ	SEQ
FLC_00321		*Troides cuneifera sumatranus*	(Hagen 1894)	Indonesia	North Sumatra, Berastagi	SEQ	SEQ	SEQ	SEQ
FLC_00322		*Troides cuneifera sumatranus*	(Hagen 1894)	Indonesia	North Sumatra, Berastagi	SEQ	SEQ	SEQ	SEQ
EC-038		*Troides cuneifera ssp.*	(Oberthür 1879)	Indonesia	-	EF514433	-	-	-
EC-044		*Troides miranda neomiranda*	(Butler 1869)	Malaysia	Borneo	EF514407	-	-	-
FLC_00324	**Subgenus Ripponia**	*Troides hypolitus cellularis*	Rothschild 1895	Indonesia	South Sulawesi, Bantimurung	SEQ	SEQ	SEQ	SEQ
FLC_00325		*Troides hypolitus cellularis*	Rothschild 1895	Indonesia	South Sulawesi, Bantimurung	SEQ	SEQ	SEQ	SEQ
FLC_00326		*Troides hypolitus antiopa*	Rothschild 1908	Indonesia	North Moluccas, Morotai Island	SEQ	SEQ	SEQ	SEQ
FLC_00327		*Troides hypolitus antiopa*	Rothschild 1908	Indonesia	North Moluccas, Morotai Island	SEQ	SEQ	SEQ	SEQ
FLC_00328		*Troides hypolitus sangirensis*	Morisode 2005	Indonesia	North Sulawesi, Sangir Island	SEQ	SEQ	SEQ	SEQ
FLC_00329		*Troides hypolitus sangirensis*	Morisode 2005	Indonesia	North Sulawesi, Sangir Island	SEQ	SEQ	SEQ	SEQ
882		*Troides hypolitus ssp.*	(Cramer 1775)	Indonesia	-	-	AB027592	-	-
TypeT1		*Troides hypolitus hypolitus*	(Cramer 1775)	Indonesia	Moluccas, Halmahera Island	-	AB084429	-	-
EC-056		*Troides hypolitus ssp.*	(Cramer 1775)	Indonesia	-	EF514416	-	-	-
EC-057		*Troides hypolitus ssp.*	(Cramer 1775)	Indonesia	-	EF514441	-	-	-
EC-009		*Ornithoptera aesacus*	Ney 1903	Indonesia	Moluccas, Obi Island	EF514431	-	-	-
EC-015		*Ornithoptera aesacus*	Ney 1903	Indonesia	Moluccas, Obi Island	EF514422	-	-	-

114	*Ornithoptera aesacus*	Ney 1903	Indonesia	Moluccas, Obi Island	-	AB044655	-	-
FLC_00341	*Ornithoptera priamus urvillenaus*	(Guérin-Méneville 1830)	Solomon	-	SEQ	SEQ	SEQ	SEQ
FLC_00342	*Ornithoptera priamus urvillenaus*	(Guérin-Méneville 1830)	Solomon	-	SEQ	SEQ	SEQ	SEQ
371	*Ornithoptera priamus urvillenaus*	(Guérin-Méneville 1830)	Solomon	Guadalcanal	.	AB044657	-	-
373	*Ornithoptera priamus urvillenaus*	(Guérin-Méneville 1830)	Solomon	Malaita	-	AB027597	-	-
TypeO10	*Ornithoptera priamus urvillenaus*	(Guérin-Méneville 1830)	Solomon	Bougainville	-	AB084441	-	-
FLC_00343	*Ornithoptera priamus poseidon*	(Doubleday 1847)	New Guinea	Irian Jaya, Mt. Arfak	SEQ	SEQ	SEQ	SEQ
FLC_00344	*Ornithoptera priamus poseidon*	(Doubleday 1847)	New Guinea	Irian Jaya, Mt. Arfak	SEQ	SEQ	SEQ	SEQ
351	*Ornithoptera priamus poseidon*	(Doubleday 1847)	New Guinea	Irian Jaya, Timika	-	AB044656	-	-
EC-010	*Ornithoptera priamus poseidon*	(Doubleday 1847)	New Guinea	-	EF514420	-	-	-
EC-014	*Ornithoptera priamus ssp.*	(Linnaeus 1758)	New Guinea	-	EF514424	-	-	-
FS-b-3776	*Ornithoptera priamus ssp.*	(Linnaeus 1758)	New Guinea	-	-	-	GQ268350	GQ268414
W122	*Ornithoptera priamus poseidon*	(Linnaeus 1758)	New Guinea	Bulolo	-	-	AM283052	-
11ANIC-07882	*Ornithoptera priamus macalpinei*	Moulds 1974	Australia	Queensland	KF397018	-	-	-
11ANIC-07883	*Ornithoptera priamus macalpinei*	Moulds 1974	Australia	Queensland	KF405508	-	-	-
11ANIC-07981	*Ornithoptera euphorion*	(Gray 1853)	Australia	Queensland, Bingup Beach near Tully	KF405060	-	-	-
RE-02-A033	*Ornithoptera euphorion*	(Gray 1853)	Australia	Queensland, Herberton	AY919291	-	-	-
11ANIC-07984	*Ornithoptera richmondia*	(Gray 1853)	Australia	New South Wales, East Ballina	KF399304	-	-	-
11ANIC-07985	*Ornithoptera richmondia*	(Gray 1853)	Australia	New South Wales, East Ballina	KF405859	-	-	-
FLC_00333	*Ornithoptera croesus lydius*	(Felder & Felder 1865)	Indonesia	Mollucas, Halmahera Island	SEQ	SEQ	SEQ	SEQ
FLC_00334	*Ornithoptera croesus lydius*	(Felder & Felder 1865)	Indonesia	Mollucas, Halmahera Island	SEQ	SEQ	SEQ	SEQ
FLC_00335	*Ornithoptera croesus lydius*	(Felder & Felder 1865)	Indonesia	Mollucas, Halmahera Island	SEQ	SEQ	SEQ	SEQ
FLC_00336	*Ornithoptera croesus lydius*	(Felder & Felder 1865)	Indonesia	Mollucas, Halmahera Island	SEQ	SEQ	SEQ	SEQ

TypeO7		*Ornithoptera croesus ssp.*	(Wallace 1859)	Indonesia	Mollucas, Halmahera Island	-	AB084438	-	-
112		*Ornithoptera croesus ssp.*	(Wallace 1859)	Indonesia	Mollucas, Halmahera Island	-	AB027595	-	-
614		*Ornithoptera croesus ssp.*	(Wallace 1859)	Indonesia	North Mollucas, Bacan Island	-	AB044654	-	-
EC-029		*Ornithoptera croesus ssp.*	(Wallace 1859)	Indonesia	North Mollucas	EF514434	-	-	-
EC-030		*Ornithoptera croesus ssp.*	(Wallace 1859)	Indonesia	North Mollucas	EF514408	-	-	-
EC-032		*Ornithoptera croesus ssp.*	(Wallace 1859)	Indonesia	North Mollucas	EF514428	-	-	-
EC-033		*Ornithoptera croesus ssp.*	(Wallace 1859)	Indonesia	North Mollucas	EF514432	-	-	-
EC-035		*Ornithoptera croesus ssp.*	(Wallace 1859)	Indonesia	North Mollucas	EF514409	-	-	-
FLC_00337		*Ornithoptera goliath samson*	(Niepelt 1913)	Indonesia	Irian Jaya, Mt. Arfak	-	SEQ	SEQ	SEQ
FLC_00338		*Ornithoptera goliath samson*	(Niepelt 1913)	Indonesia	Irian Jaya, Mt. Arfak	-	SEQ	SEQ	SEQ
FLC_00358		*Ornithoptera goliath supremus*	Röber 1896	New Guinea	Aseki, Morobe Province	SEQ	SEQ	SEQ	SEQ
FLC_00359		*Ornithoptera goliath supremus*	Röber 1896	New Guinea	Aseki, Morobe Province	SEQ	SEQ	SEQ	SEQ
EC-047		*Ornithoptera goliath ssp.*	Oberthür 1888	New Guinea	-	EF514414	-	-	-
EC-049		*Ornithoptera goliath ssp.*	Oberthür 1888	New Guinea	-	EF514418	-	-	-
EC-087		*Ornithoptera goliath ssp.*	Oberthür 1888	New Guinea	-	EF514447	-	-	-
TypeO2	a	*Ornithoptera goliath ssp.*	Oberthür 1888	New Guinea	Irian Jaya	-	AB084433	-	-
110		*Ornithoptera goliath ssp.*	Oberthür 1888	Indonesia	-	-	AB027600	-	-
398		*Ornithoptera goliath procus*	(Rothschild 1914)	Indonesia	Moluccas, Seram Island	-	AB027599	-	-
TypeO6		*Ornithoptera meridionalis meridionalis*	(Rothschild 1897)	New Guinea	-	-	AB084437	-	-
193		*Ornithoptera meridionalis tarunggarensis*	(Joicey & Talbot 1927)	New Guinea	Irian Jaya, Timika	-	AB044653	-	-
EC-031		*Ornithoptera paradisea*	(Staudinger 1893)	New Guinea	-	EF514452	-	-	-
EC-037		*Ornithoptera paradisea*	(Staudinger 1893)	New Guinea	-	EF514461	-	-	-
196		*Ornithoptera paradisea*	(Staudinger 1893)	New Guinea	-	-	AB027603	-	-

TypeO4	*Ornithoptera paradisea*	(Staudinger 1893)	New Guinea	Irian Jaya	-	AB084435	-	-
-	*Ornithoptera arfakensis*	(Joicey & Noakes 1916)	New Guinea	Irian Jaya, Mt. Arfak	-	AB044659	-	-
EC-053	*Ornithoptera chimaera ssp.*	(Rothschild 1904)	New Guinea	-	EF514411	-	-	-
EC-055	*Ornithoptera chimaera ssp.*	(Rothschild 1904)	New Guinea	-	EF514445	-	-	-
198	*Ornithoptera chimaera charybdis*	(van Eecke 1915)	New Guinea	Irian Jaya, Mt. Wayland	-	AB044660	-	-
FLC_00348	*Ornithoptera rothschildi*	(Kenrick 1911)	New Guinea	Irian Jaya, Mt. Arfak	SEQ	SEQ	SEQ	SEQ
FLC_00349	*Ornithoptera rothschildi*	(Kenrick 1911)	New Guinea	Irian Jaya, Mt. Arfak	SEQ	SEQ	SEQ	SEQ
FLC_00350	*Ornithoptera rothschildi*	(Kenrick 1911)	New Guinea	Irian Jaya, Mt. Arfak	SEQ	SEQ	SEQ	SEQ
FLC_00351	*Ornithoptera rothschildi*	(Kenrick 1911)	New Guinea	Irian Jaya, Mt. Arfak	SEQ	SEQ	SEQ	SEQ
TypeO3	*Ornithoptera rothschildi*	(Kenrick 1911)	New Guinea	Irian Jaya, Mt. Arfak	-	AB084434	-	-
-	*Ornithoptera rothschildi*	(Kenrick 1911)	New Guinea	-	-	AB027601	-	-
FLC_00353	*Ornithoptera tithonus misresiana*	(Joicey & Noakes 1916)	New Guinea	Irian Jaya, Mt. Arfak	-	SEQ	SEQ	-
EC-050	*Ornithoptera tithonus ssp.*	(de Haan 1840)	New Guinea	-	EF514462	-	-	-
EC-051	*Ornithoptera tithonus ssp.*	(de Haan 1840)	New Guinea	-	EF514460	-	-	-
EC-052	*Ornithoptera tithonus ssp.*	(de Haan 1840)	New Guinea	-	EF514459	-	-	-
TypeO1	*Ornithoptera tithonus ssp.*	(de Haan 1840)	New Guinea	Irian Jaya	-	AB084432	-	-
-	*Ornithoptera tithonus ssp.*	(de Haan 1840)	New Guinea	-	-	AB027602	-	-
TypeO12	*Ornithoptera alexandrae*	Rothschild 1907	New Guinea	Popondetta	-	AB084443	-	-
366	*Ornithoptera victoriae*	(Gray 1856)	Solomon	Gela	-	AB044658	-	-
TypeO8	*Ornithoptera victoriae*	(Gray 1856)	Solomon	Bougainville	-	AB084439	-	-
FLC_00356	*Trogonoptera brookiana*	(Wallace 1855)	Indonesia	Borneo, North Sabath, Crooker range	-	-	-	-
EC-091	*Trogonoptera brookiana*	(Wallace 1855)	Indonesia	-	EF514439	-	-	-
531	*Trogonoptera brookiana*	(Wallace 1855)	Indonesia	Sumatra, Padang	-	AB044647	-	-

312		*Trogonoptera brookiana*	(Wallace 1855)	Malaysia	Cameron Highlands	-	AB044648	-	-
-		*Trogonoptera brookiana*	(Wallace 1855)	Malaysia	Cameron Highlands	-	AB084428	-	-
EC-089		*Trogonoptera trojana*	(Staudinger 1889)	Philippines	Lanjuan, Palawan	EF514444	-	-	-
EC-090		*Trogonoptera trojana*	(Staudinger 1889)	Philippines	Lanjuan, Palawan	EF514436	-	-	-

Figure S1. (a) Geographic distribution of *Ornithoptera priamus* and its subspecies. Adapted from Haugum and Low[1]. Map drawn with PowerPoint.

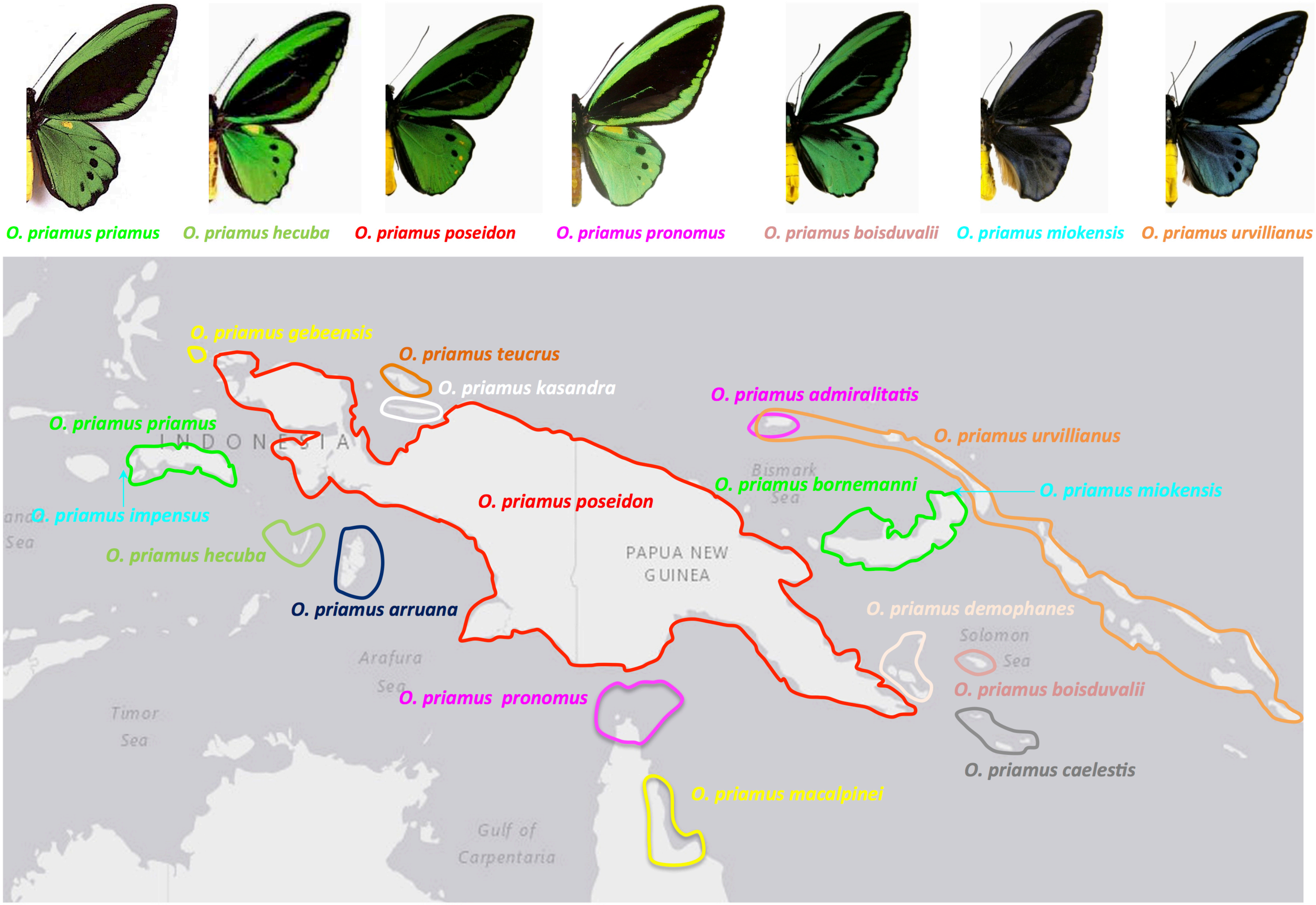

Figure S1. (b) Geographic distribution of *Troides helena* and its subspecies. Adapted from Haugum and Low[1]. Map drawn with PowerPoint.

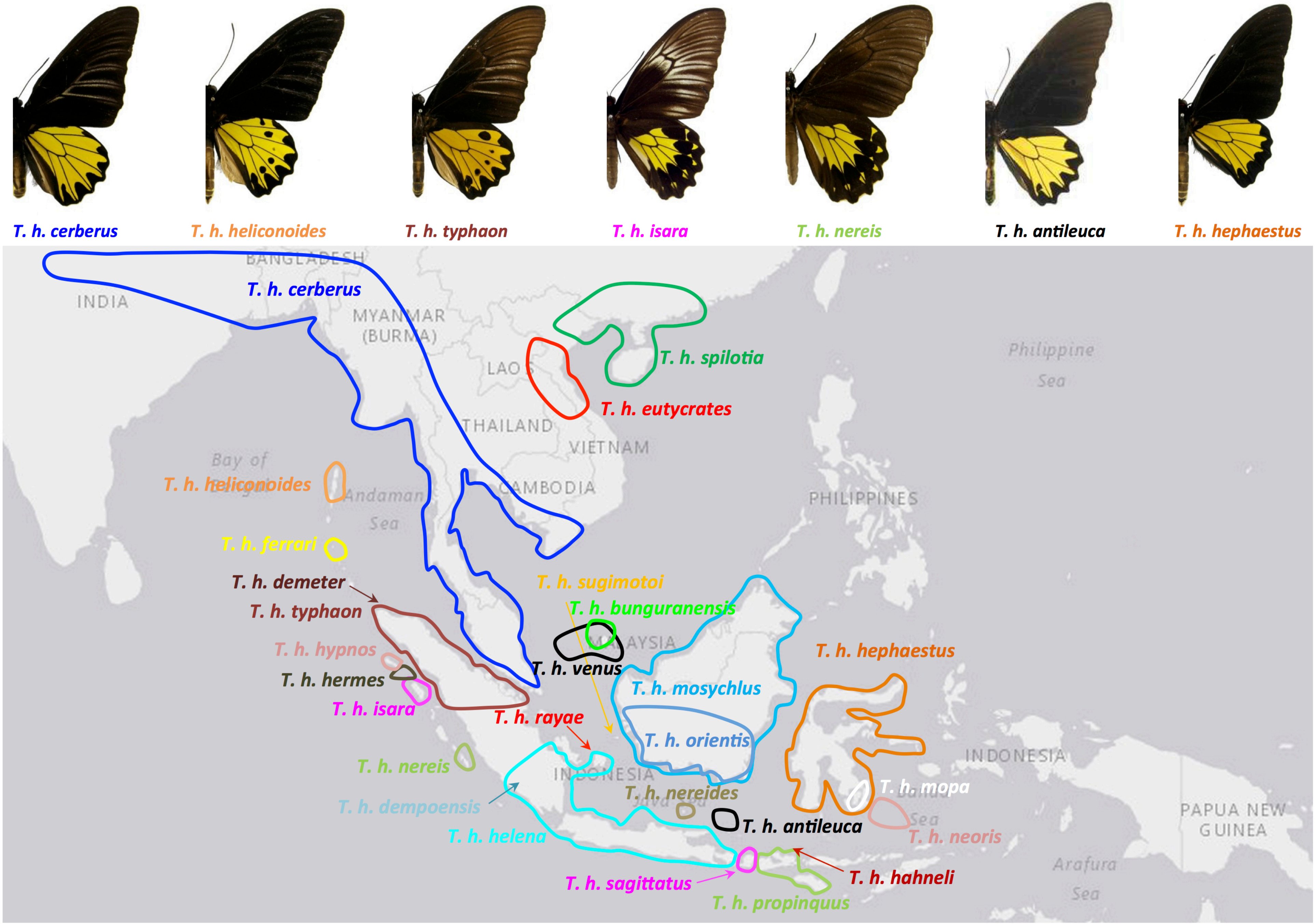

Figure S2. (a) Bayesian phylogeny of the COI gene.

Figure S2. (b) Bayesian phylogeny of the ND5 gene.

Figure S2. (c) Bayesian phylogeny of the 16S gene.

Figure S2. (d) Bayesian phylogeny of the EF-1α gene.

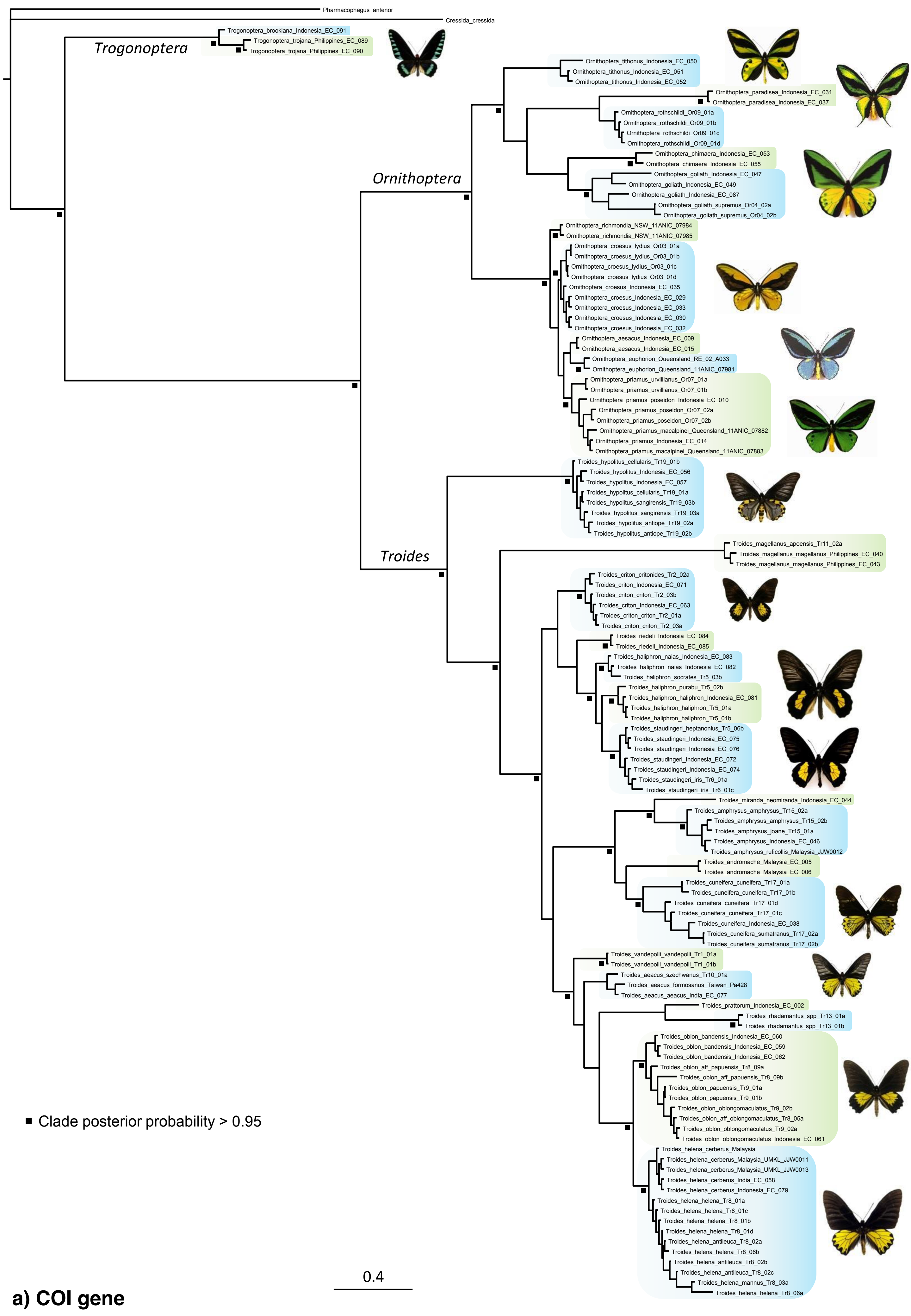
Pharmacophagus_antenor
Cressida_cressida
Trogonoptera
Trogonoptera_brookiana_Indonesia_EC_091
Trogonoptera_trojana_Philippines_EC_089
Trogonoptera_trojana_Philippines_EC_090
Ornithoptera
Ornithoptera_tithonus_Indonesia_EC_050
Ornithoptera_tithonus_Indonesia_EC_051
Ornithoptera_tithonus_Indonesia_EC_052
Ornithoptera_paradisea_Indonesia_EC_031
Ornithoptera_paradisea_Indonesia_EC_037
Ornithoptera_rothschildi_Or09_01a
Ornithoptera_rothschildi_Or09_01b
Ornithoptera_rothschildi_Or09_01c
Ornithoptera_rothschildi_Or09_01d
Ornithoptera_chimaera_Indonesia_EC_053
Ornithoptera_chimaera_Indonesia_EC_055
Ornithoptera_goliath_Indonesia_EC_047
Ornithoptera_goliath_Indonesia_EC_049
Ornithoptera_goliath_Indonesia_EC_087
Ornithoptera_goliath_supremus_Or04_02a
Ornithoptera_goliath_supremus_Or04_02b
Ornithoptera_richmondia_NSW_11ANIC_07984
Ornithoptera_richmondia_NSW_11ANIC_07985
Ornithoptera_croesus_lydius_Or03_01a
Ornithoptera_croesus_lydius_Or03_01b
Ornithoptera_croesus_lydius_Or03_01c
Ornithoptera_croesus_lydius_Or03_01d
Ornithoptera_croesus_Indonesia_EC_035
Ornithoptera_croesus_Indonesia_EC_029
Ornithoptera_croesus_Indonesia_EC_033
Ornithoptera_croesus_Indonesia_EC_030
Ornithoptera_croesus_Indonesia_EC_032
Ornithoptera_aesacus_Indonesia_EC_009
Ornithoptera_aesacus_Indonesia_EC_015
Ornithoptera_euphorion_Queensland_RE_02_A033
Ornithoptera_euphorion_Queensland_11ANIC_07981
Ornithoptera_priamus_urvillianus_Or07_01a
Ornithoptera_priamus_urvillianus_Or07_01b
Ornithoptera_priamus_poseidon_Indonesia_EC_010
Ornithoptera_priamus_poseidon_Or07_02a
Ornithoptera_priamus_poseidon_Or07_02b
Ornithoptera_priamus_macalpinei_Queensland_11ANIC_07882
Ornithoptera_priamus_Indonesia_EC_014
Ornithoptera_priamus_macalpinei_Queensland_11ANIC_07883
Troides
Troides_hypolitus_cellularis_Tr19_01b
Troides_hypolitus_Indonesia_EC_056
Troides_hypolitus_Indonesia_EC_057
Troides_hypolitus_cellularis_Tr19_01a
Troides_hypolitus_sangirensis_Tr19_03b
Troides_hypolitus_sangirensis_Tr19_03a
Troides_hypolitus_antiope_Tr19_02a
Troides_hypolitus_antiope_Tr19_02b
Troides_magellanus_apoensis_Tr11_02a
Troides_magellanus_magellanus_Philippines_EC_040
Troides_magellanus_magellanus_Philippines_EC_043
Troides_criton_critonides_Tr2_02a
Troides_criton_Indonesia_EC_071
Troides_criton_criton_Tr2_03b
Troides_criton_Indonesia_EC_063
Troides_criton_criton_Tr2_01a
Troides_criton_criton_Tr2_03a
Troides_riedeli_Indonesia_EC_084
Troides_riedeli_Indonesia_EC_085
Troides_haliphron_naias_Indonesia_EC_083
Troides_haliphron_naias_Indonesia_EC_082
Troides_haliphron_socrates_Tr5_03b
Troides_haliphron_purabu_Tr5_02b
Troides_haliphron_haliphron_Indonesia_EC_081
Troides_haliphron_haliphron_Tr5_01a
Troides_haliphron_haliphron_Tr5_01b
Troides_staudingeri_heptanonius_Tr5_06b
Troides_staudingeri_Indonesia_EC_075
Troides_staudingeri_Indonesia_EC_076
Troides_staudingeri_Indonesia_EC_072
Troides_staudingeri_Indonesia_EC_074
Troides_staudingeri_iris_Tr6_01a
Troides_staudingeri_iris_Tr6_01c
Troides_miranda_neomiranda_Indonesia_EC_044
Troides_amphrysus_amphrysus_Tr15_02a
Troides_amphrysus_amphrysus_Tr15_02b
Troides_amphrysus_joane_Tr15_01a
Troides_amphrysus_Indonesia_EC_046
Troides_amphrysus_ruficollis_Malaysia_JJW0012
Troides_andromache_Malaysia_EC_005
Troides_andromache_Malaysia_EC_006
Troides_cuneifera_cuneifera_Tr17_01a
Troides_cuneifera_cuneifera_Tr17_01b
Troides_cuneifera_cuneifera_Tr17_01d
Troides_cuneifera_cuneifera_Tr17_01c
Troides_cuneifera_Indonesia_EC_038
Troides_cuneifera_sumatranus_Tr17_02a
Troides_cuneifera_sumatranus_Tr17_02b
Troides_vandepolli_vandepolli_Tr1_01a
Troides_vandepolli_vandepolli_Tr1_01b
Troides_aeacus_szechwanus_Tr10_01a
Troides_aeacus_formosanus_Taiwan_Pa428
Troides_aeacus_aeacus_India_EC_077
Troides_prattorum_Indonesia_EC_002
Troides_rhadamantus_spp_Tr13_01a
Troides_rhadamantus_spp_Tr13_01b
Troides_oblon_bandensis_Indonesia_EC_060
Troides_oblon_bandensis_Indonesia_EC_059
Troides_oblon_bandensis_Indonesia_EC_062
Troides_oblon_aff_papuensis_Tr8_09a
Troides_oblon_aff_papuensis_Tr8_09b
Troides_oblon_papuensis_Tr9_01a
Troides_oblon_papuensis_Tr9_01b
Troides_oblon_oblongomaculatus_Tr9_02b
Troides_oblon_aff_oblongomaculatus_Tr8_05a
Troides_oblon_oblongomaculatus_Tr9_02a
Troides_oblon_oblongomaculatus_Indonesia_EC_061
Troides_helena_cerberus_Malaysia
Troides_helena_cerberus_Malaysia_UMKL_JJW0011
Troides_helena_cerberus_Malaysia_UMKL_JJW0013
Troides_helena_cerberus_India_EC_058
Troides_helena_cerberus_Indonesia_EC_079
Troides_helena_helena_Tr8_01a
Troides_helena_helena_Tr8_01c
Troides_helena_helena_Tr8_01b
Troides_helena_helena_Tr8_01d
Troides_helena_antileuca_Tr8_02a
Troides_helena_helena_Tr8_06b
Troides_helena_antileuca_Tr8_02b
Troides_helena_antileuca_Tr8_02c
Troides_helena_mannus_Tr8_03a
Troides_helena_helena_Tr8_06a
■ Clade posterior probability > 0.95
0.4
a) COI gene

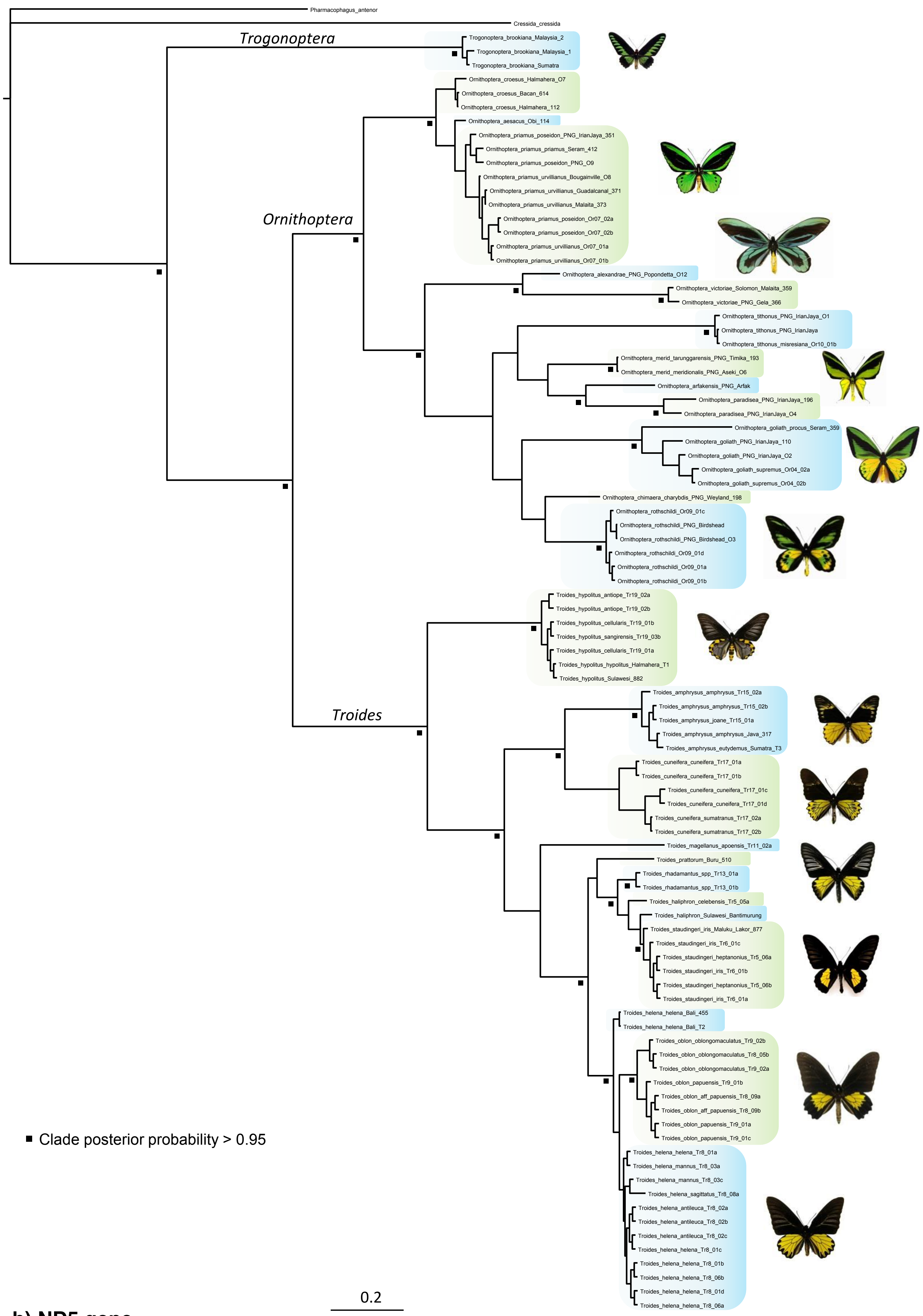

Pharmacophagus_antenor
Cressida_cressida
Trogonoptera
Trogonoptera_brookiana_Malaysia_2
Trogonoptera_brookiana_Malaysia_1
Trogonoptera_brookiana_Sumatra
Ornithoptera_croesus_Halmahera_O7
Ornithoptera_croesus_Bacan_614
Ornithoptera_croesus_Halmahera_112
Ornithoptera_aesacus_Obi_114
Ornithoptera_priamus_poseidon_PNG_IrianJaya_351
Ornithoptera_priamus_priamus_Seram_412
Ornithoptera_priamus_poseidon_PNG_O9
Ornithoptera_priamus_urvillianus_Bougainville_O8
Ornithoptera_priamus_urvillianus_Guadalcanal_371
Ornithoptera_priamus_urvillianus_Malaita_373
Ornithoptera
Ornithoptera_priamus_poseidon_Or07_02a
Ornithoptera_priamus_poseidon_Or07_02b
Ornithoptera_priamus_urvillianus_Or07_01a
Ornithoptera_priamus_urvillianus_Or07_01b
Ornithoptera_alexandrae_PNG_Popondetta_O12
Ornithoptera_victoriae_Solomon_Malaita_359
Ornithoptera_victoriae_PNG_Gela_366
Ornithoptera_tithonus_PNG_IrianJaya_O1
Ornithoptera_tithonus_PNG_IrianJaya
Ornithoptera_tithonus_misresiana_Or10_01b
Ornithoptera_merid_tarunggarensis_PNG_Timika_193
Ornithoptera_merid_meridionalis_PNG_Aseki_O6
Ornithoptera_arfakensis_PNG_Arfak
Ornithoptera_paradisea_PNG_IrianJaya_196
Ornithoptera_paradisea_PNG_IrianJaya_O4
Ornithoptera_goliath_procus_Seram_359
Ornithoptera_goliath_PNG_IrianJaya_110
Ornithoptera_goliath_PNG_IrianJaya_O2
Ornithoptera_goliath_supremus_Or04_02a
Ornithoptera_goliath_supremus_Or04_02b
Ornithoptera_chimaera_charybdis_PNG_Weyland_198
Ornithoptera_rothschildi_Or09_01c
Ornithoptera_rothschildi_PNG_Birdshead
Ornithoptera_rothschildi_PNG_Birdshead_O3
Ornithoptera_rothschildi_Or09_01d
Ornithoptera_rothschildi_Or09_01a
Ornithoptera_rothschildi_Or09_01b
Troides_hypolitus_antiope_Tr19_02a
Troides_hypolitus_antiope_Tr19_02b
Troides_hypolitus_cellularis_Tr19_01b
Troides_hypolitus_sangirensis_Tr19_03b
Troides_hypolitus_cellularis_Tr19_01a
Troides_hypolitus_hypolitus_Halmahera_T1
Troides_hypolitus_Sulawesi_882
Troides_amphrysus_amphrysus_Tr15_02a
Troides_amphrysus_amphrysus_Tr15_02b
Troides_amphrysus_joane_Tr15_01a
Troides_amphrysus_amphrysus_Java_317
Troides_amphrysus_eutydemus_Sumatra_T3
Troides
Troides_cuneifera_cuneifera_Tr17_01a
Troides_cuneifera_cuneifera_Tr17_01b
Troides_cuneifera_cuneifera_Tr17_01c
Troides_cuneifera_cuneifera_Tr17_01d
Troides_cuneifera_sumatranus_Tr17_02a
Troides_cuneifera_sumatranus_Tr17_02b
Troides_magellanus_apoensis_Tr11_02a
Troides_prattorum_Buru_510
Troides_rhadamantus_spp_Tr13_01a
Troides_rhadamantus_spp_Tr13_01b
Troides_haliphron_celebensis_Tr5_05a
Troides_haliphron_Sulawesi_Bantimurung
Troides_staudingeri_iris_Maluku_Lakor_877
Troides_staudingeri_iris_Tr6_01c
Troides_staudingeri_heptanonius_Tr5_06a
Troides_staudingeri_iris_Tr6_01b
Troides_staudingeri_heptanonius_Tr5_06b
Troides_staudingeri_iris_Tr6_01a
Troides_helena_helena_Bali_455
Troides_helena_helena_Bali_T2
Troides_oblon_oblongomaculatus_Tr9_02b
Troides_oblon_oblongomaculatus_Tr8_05b
Troides_oblon_oblongomaculatus_Tr9_02a
Troides_oblon_papuensis_Tr9_01b
Troides_oblon_aff_papuensis_Tr8_09a
Troides_oblon_aff_papuensis_Tr8_09b
Troides_oblon_papuensis_Tr9_01a
Troides_oblon_papuensis_Tr9_01c
Clade posterior probability > 0.95
Troides_helena_helena_Tr8_01a
Troides_helena_mannus_Tr8_03a
Troides_helena_mannus_Tr8_03c
Troides_helena_sagittatus_Tr8_08a
Troides_helena_antileuca_Tr8_02a
Troides_helena_antileuca_Tr8_02b
Troides_helena_antileuca_Tr8_02c
Troides_helena_helena_Tr8_01c
Troides_helena_helena_Tr8_01b
Troides_helena_helena_Tr8_06b
Troides_helena_helena_Tr8_01d
Troides_helena_helena_Tr8_06a
0.2

b) ND5 gene

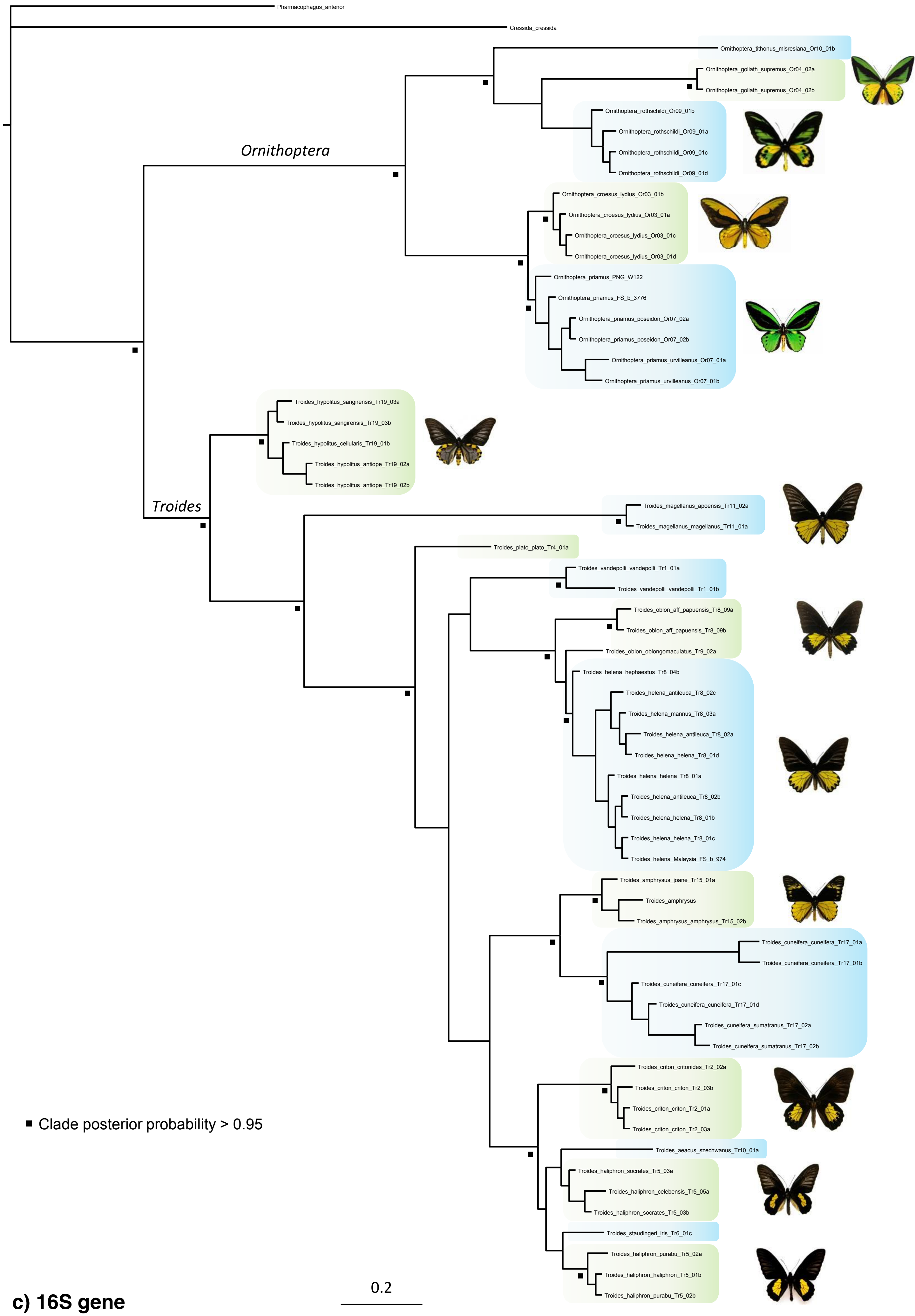
Pharmacophagus_antenor
Cressida_cressida
Ornithoptera
Ornithoptera_tithonus_misresiana_Or10_01b
Ornithoptera_goliath_supremus_Or04_02a
Ornithoptera_goliath_supremus_Or04_02b
Ornithoptera_rothschildi_Or09_01b
Ornithoptera_rothschildi_Or09_01a
Ornithoptera_rothschildi_Or09_01c
Ornithoptera_rothschildi_Or09_01d
Ornithoptera_croesus_lydius_Or03_01b
Ornithoptera_croesus_lydius_Or03_01a
Ornithoptera_croesus_lydius_Or03_01c
Ornithoptera_croesus_lydius_Or03_01d
Ornithoptera_priamus_PNG_W122
Ornithoptera_priamus_FS_b_3776
Ornithoptera_priamus_poseidon_Or07_02a
Ornithoptera_priamus_poseidon_Or07_02b
Ornithoptera_priamus_urvilleanus_Or07_01a
Ornithoptera_priamus_urvilleanus_Or07_01b
Troides
Troides_hypolitus_sangirensis_Tr19_03a
Troides_hypolitus_sangirensis_Tr19_03b
Troides_hypolitus_cellularis_Tr19_01b
Troides_hypolitus_antiope_Tr19_02a
Troides_hypolitus_antiope_Tr19_02b
Troides_magellanus_apoensis_Tr11_02a
Troides_magellanus_magellanus_Tr11_01a
Troides_plato_plato_Tr4_01a
Troides_vandepolli_vandepolli_Tr1_01a
Troides_vandepolli_vandepolli_Tr1_01b
Troides_oblon_aff_papuensis_Tr8_09a
Troides_oblon_aff_papuensis_Tr8_09b
Troides_oblon_oblongomaculatus_Tr9_02a
Troides_helena_hephaestus_Tr8_04b
Troides_helena_antileuca_Tr8_02c
Troides_helena_mannus_Tr8_03a
Troides_helena_antileuca_Tr8_02a
Troides_helena_helena_Tr8_01d
Troides_helena_helena_Tr8_01a
Troides_helena_antileuca_Tr8_02b
Troides_helena_helena_Tr8_01b
Troides_helena_helena_Tr8_01c
Troides_helena_Malaysia_FS_b_974
Troides_amphrysus_joane_Tr15_01a
Troides_amphrysus
Troides_amphrysus_amphrysus_Tr15_02b
Troides_cuneifera_cuneifera_Tr17_01a
Troides_cuneifera_cuneifera_Tr17_01b
Troides_cuneifera_cuneifera_Tr17_01c
Troides_cuneifera_cuneifera_Tr17_01d
Troides_cuneifera_sumatranus_Tr17_02a
Troides_cuneifera_sumatranus_Tr17_02b
Troides_criton_critonides_Tr2_02a
Troides_criton_criton_Tr2_03b
Troides_criton_criton_Tr2_01a
Troides_criton_criton_Tr2_03a
Troides_aeacus_szechwanus_Tr10_01a
Troides_haliphron_socrates_Tr5_03a
Troides_haliphron_celebensis_Tr5_05a
Troides_haliphron_socrates_Tr5_03b
Troides_staudingeri_iris_Tr6_01c
Troides_haliphron_purabu_Tr5_02a
Troides_haliphron_haliphron_Tr5_01b
Troides_haliphron_purabu_Tr5_02b
■ Clade posterior probability > 0.95
0.2
c) 16S gene

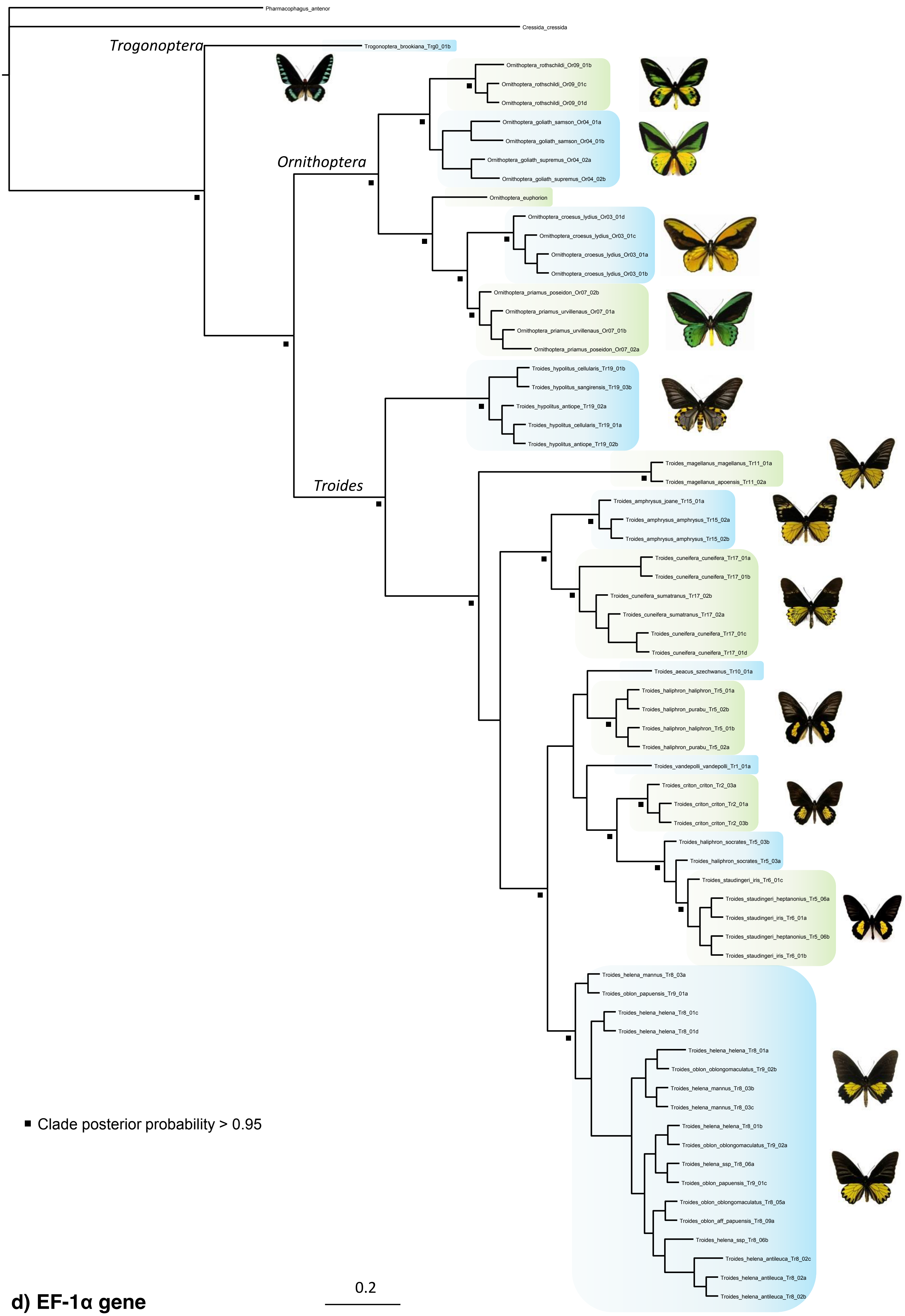

Pharmacophagus_antenor
Cressida_cressida
Trogonoptera
Trogonoptera_brookiana_Trg0_01b
Ornithoptera_rothschildi_Or09_01b
Ornithoptera_rothschildi_Or09_01c
Ornithoptera_rothschildi_Or09_01d
Ornithoptera_goliath_samson_Or04_01a
Ornithoptera_goliath_samson_Or04_01b
Ornithoptera
Ornithoptera_goliath_supremus_Or04_02a
Ornithoptera_goliath_supremus_Or04_02b
Ornithoptera_euphorion
Ornithoptera_croesus_lydius_Or03_01d
Ornithoptera_croesus_lydius_Or03_01c
Ornithoptera_croesus_lydius_Or03_01a
Ornithoptera_croesus_lydius_Or03_01b
Ornithoptera_priamus_poseidon_Or07_02b
Ornithoptera_priamus_urvillenaus_Or07_01a
Ornithoptera_priamus_urvillenaus_Or07_01b
Ornithoptera_priamus_poseidon_Or07_02a
Troides_hypolitus_cellularis_Tr19_01b
Troides_hypolitus_sangirensis_Tr19_03b
Troides_hypolitus_antiope_Tr19_02a
Troides_hypolitus_cellularis_Tr19_01a
Troides_hypolitus_antiope_Tr19_02b
Troides_magellanus_magellanus_Tr11_01a
Troides
Troides_magellanus_apoensis_Tr11_02a
Troides_amphrysus_joane_Tr15_01a
Troides_amphrysus_amphrysus_Tr15_02a
Troides_amphrysus_amphrysus_Tr15_02b
Troides_cuneifera_cuneifera_Tr17_01a
Troides_cuneifera_cuneifera_Tr17_01b
Troides_cuneifera_sumatranus_Tr17_02b
Troides_cuneifera_sumatranus_Tr17_02a
Troides_cuneifera_cuneifera_Tr17_01c
Troides_cuneifera_cuneifera_Tr17_01d
Troides_aeacus_szechwanus_Tr10_01a
Troides_haliphron_haliphron_Tr5_01a
Troides_haliphron_purabu_Tr5_02b
Troides_haliphron_haliphron_Tr5_01b
Troides_haliphron_purabu_Tr5_02a
Troides_vandepolli_vandepolli_Tr1_01a
Troides_criton_criton_Tr2_03a
Troides_criton_criton_Tr2_01a
Troides_criton_criton_Tr2_03b
Troides_haliphron_socrates_Tr5_03b
Troides_haliphron_socrates_Tr5_03a
Troides_staudingeri_iris_Tr6_01c
Troides_staudingeri_heptanonius_Tr5_06a
Troides_staudingeri_iris_Tr6_01a
Troides_staudingeri_heptanonius_Tr5_06b
Troides_staudingeri_iris_Tr6_01b
Troides_helena_mannus_Tr8_03a
Troides_oblon_papuensis_Tr9_01a
Troides_helena_helena_Tr8_01c
Troides_helena_helena_Tr8_01d
Troides_helena_helena_Tr8_01a
Troides_oblon_oblongomaculatus_Tr9_02b
Troides_helena_mannus_Tr8_03b
Troides_helena_mannus_Tr8_03c
Clade posterior probability > 0.95
Troides_helena_helena_Tr8_01b
Troides_oblon_oblongomaculatus_Tr9_02a
Troides_helena_ssp_Tr8_06a
Troides_oblon_papuensis_Tr9_01c
Troides_oblon_oblongomaculatus_Tr8_05a
Troides_oblon_aff_papuensis_Tr8_09a
Troides_helena_ssp_Tr8_06b
Troides_helena_antileuca_Tr8_02c
Troides_helena_antileuca_Tr8_02a
Troides_helena_antileuca_Tr8_02b
0.2

d) EF-1α gene

Figure S3. Biogeographical reconstructions as estimated with DEC, DEC+J, DIVA-like, DIVA-like+J, BayArea-like and BayArea-like+J models. The best model is DEC+J (see Table S1).

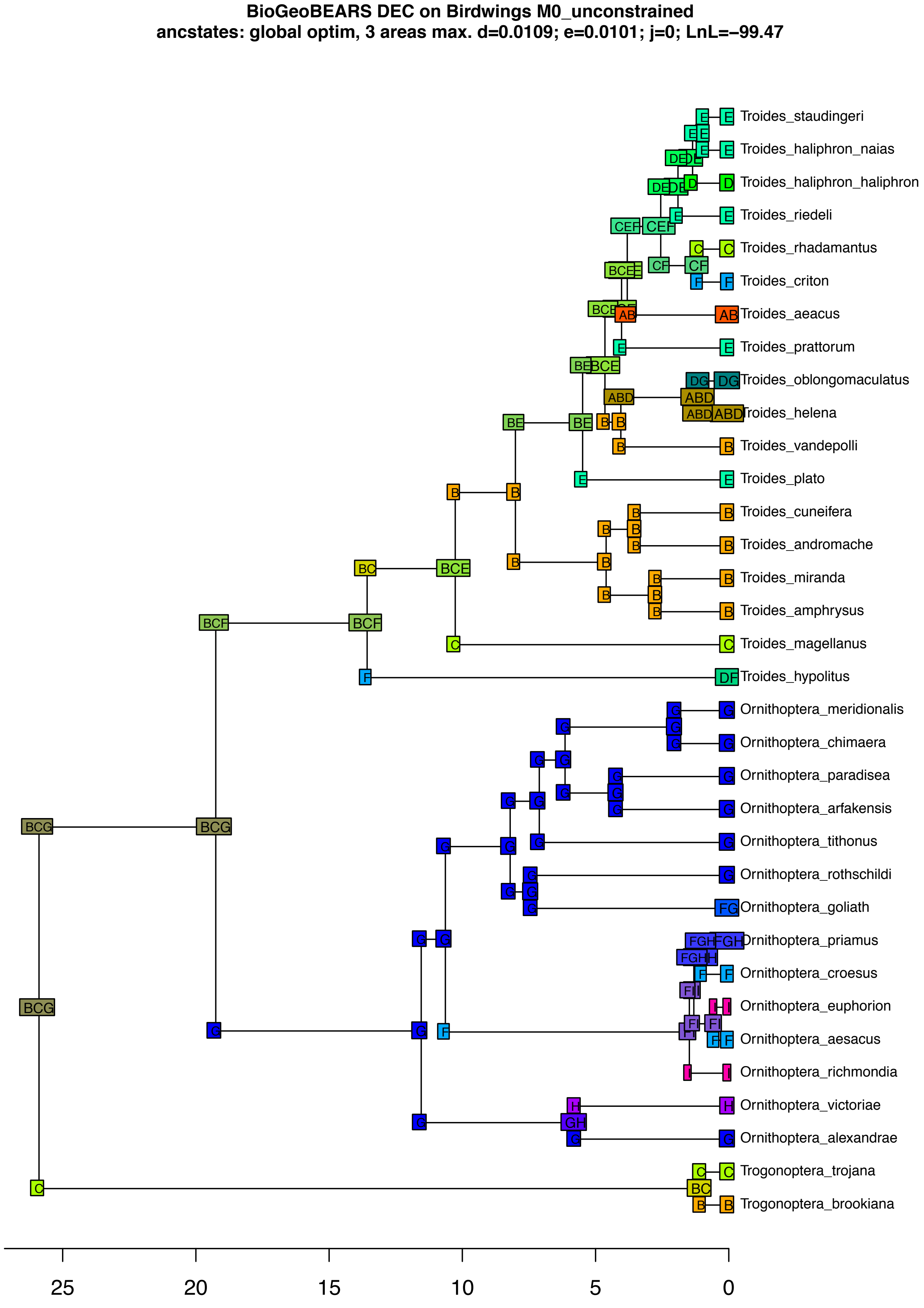

BioGeoBEARS DEC on Birdwings M0_unconstrained
ancstates: global optim, 3 areas max. d=0.0109; e=0.0101; j=0; LnL=-99.47

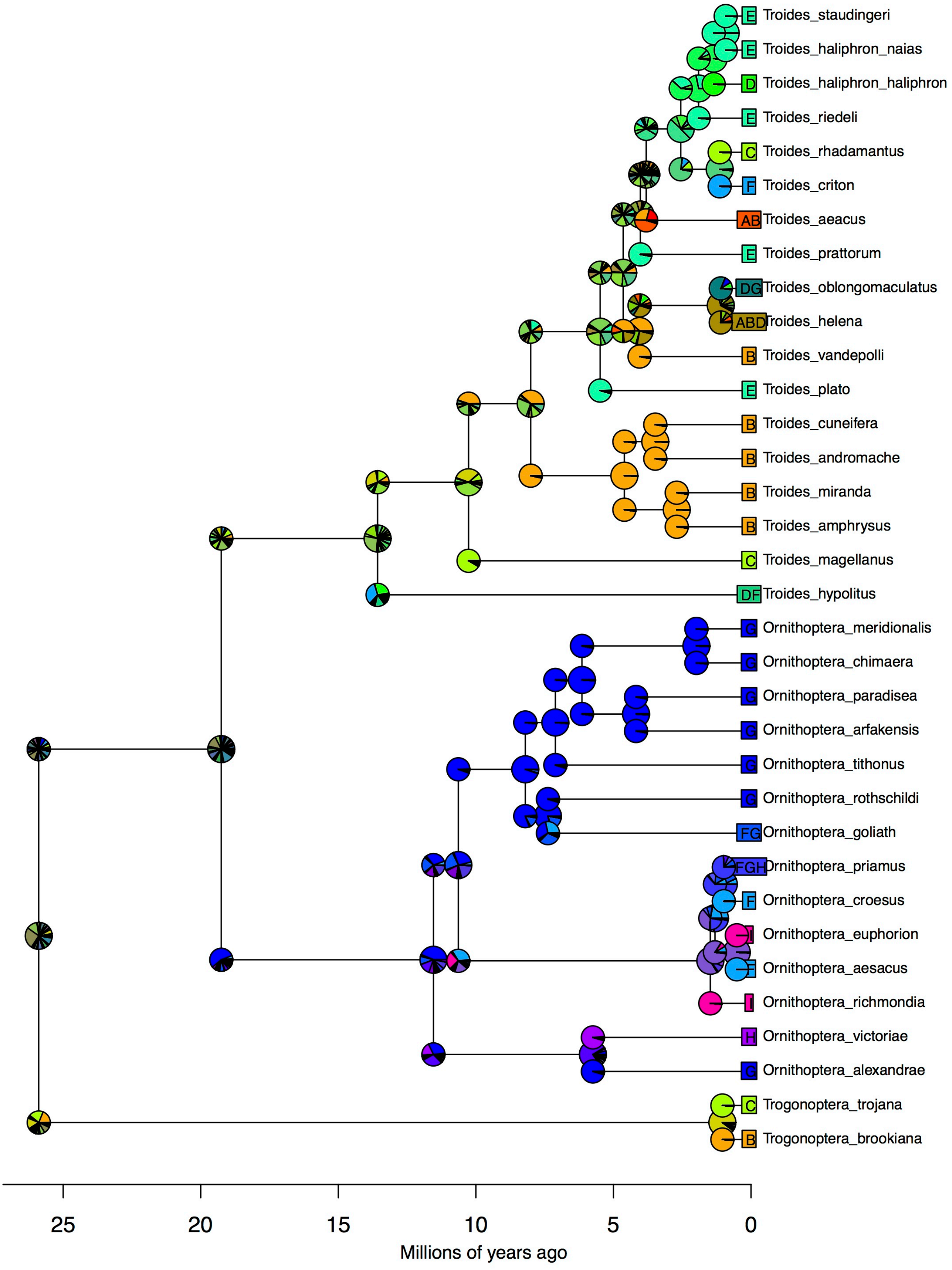

BioGeoBEARS DEC+J on Birdwings M0_unconstrained
ancstates: global optim, 3 areas max. d=0.0055; e=0; j=0.0432; LnL=-90.80

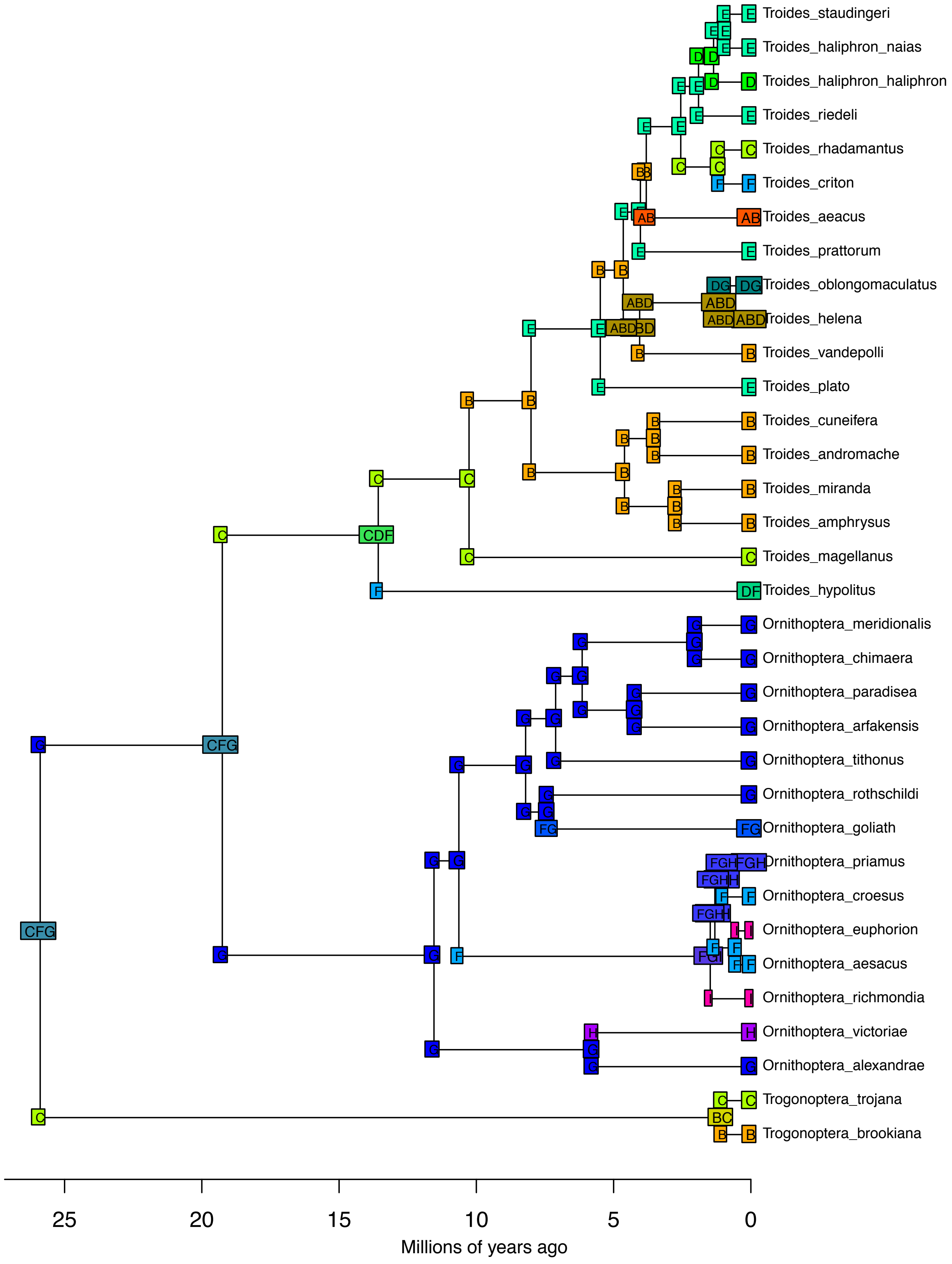

BioGeoBEARS DEC+J on Birdwings M0_unconstrained
ancstates: global optim, 3 areas max. d=0.0055; e=0; j=0.0432; LnL=-90.80

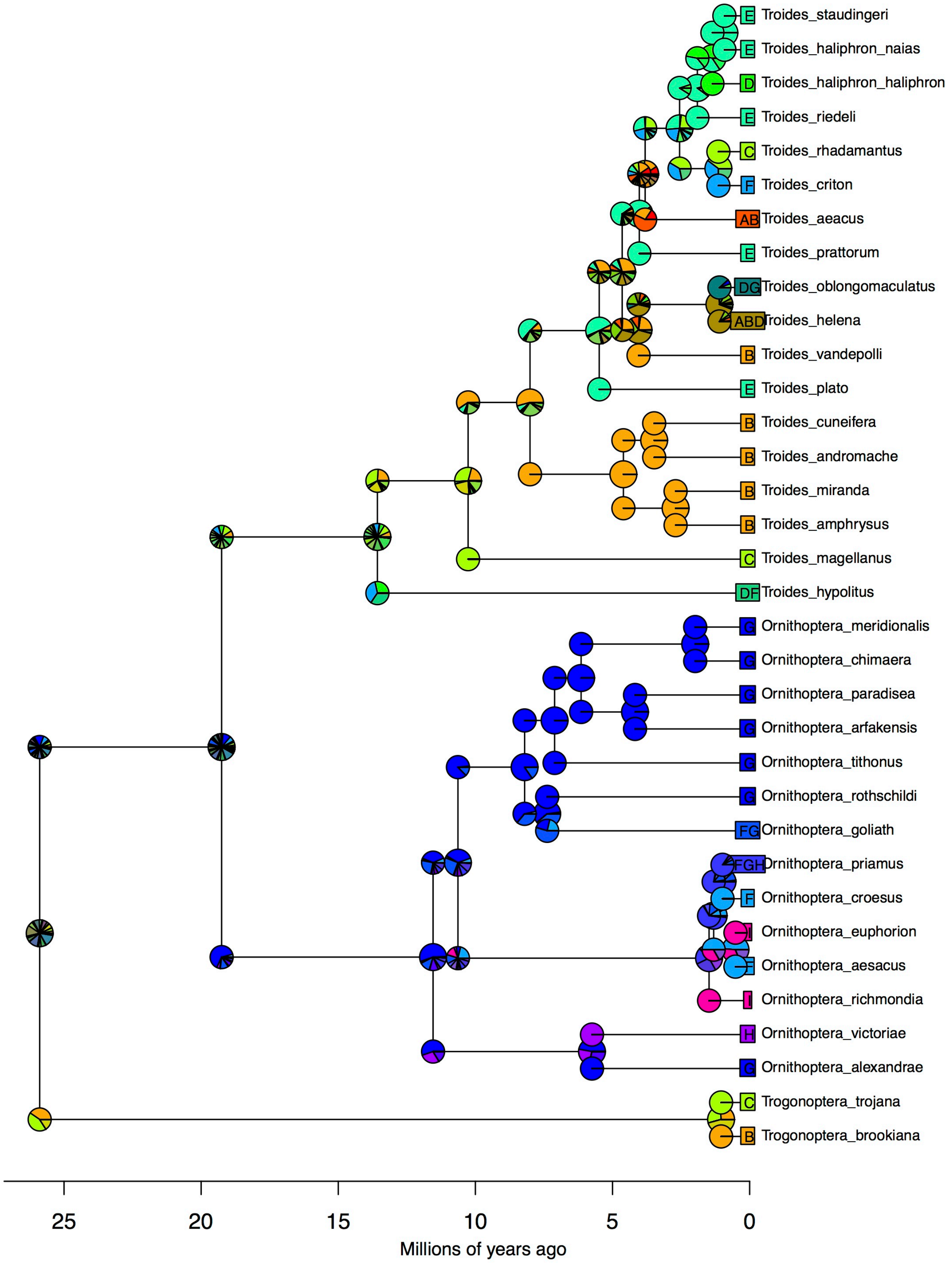

BioGeoBEARS DIVALIKE on Birdwings M0_unconstrained
ancstates: global optim, 3 areas max. d=0.0143; e=0.0136; j=0; LnL=−101.55

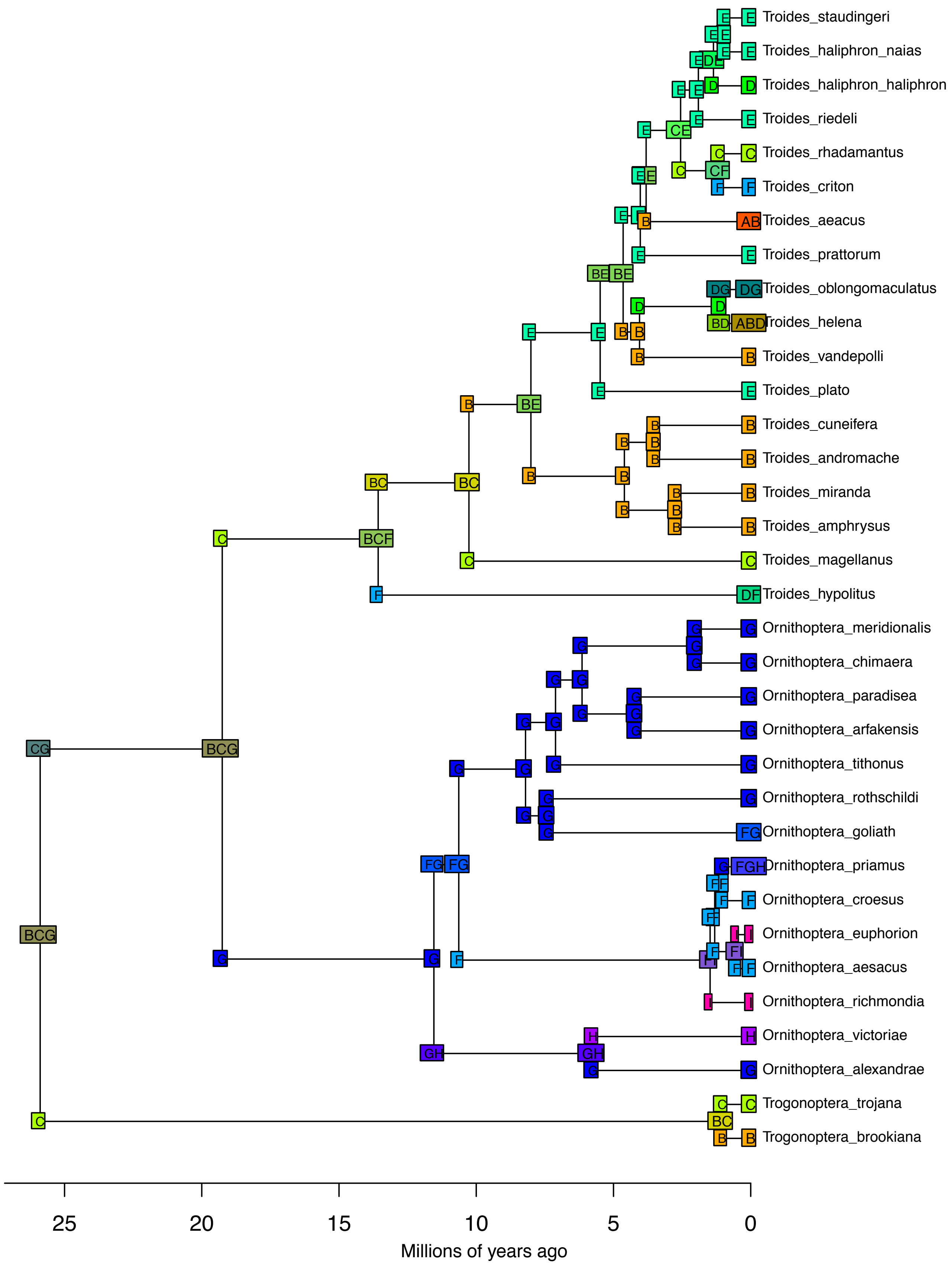

BioGeoBEARS DIVALIKE on Birdwings M0_unconstrained
ancstates: global optim, 3 areas max. d=0.0143; e=0.0136; j=0; LnL=−101.55

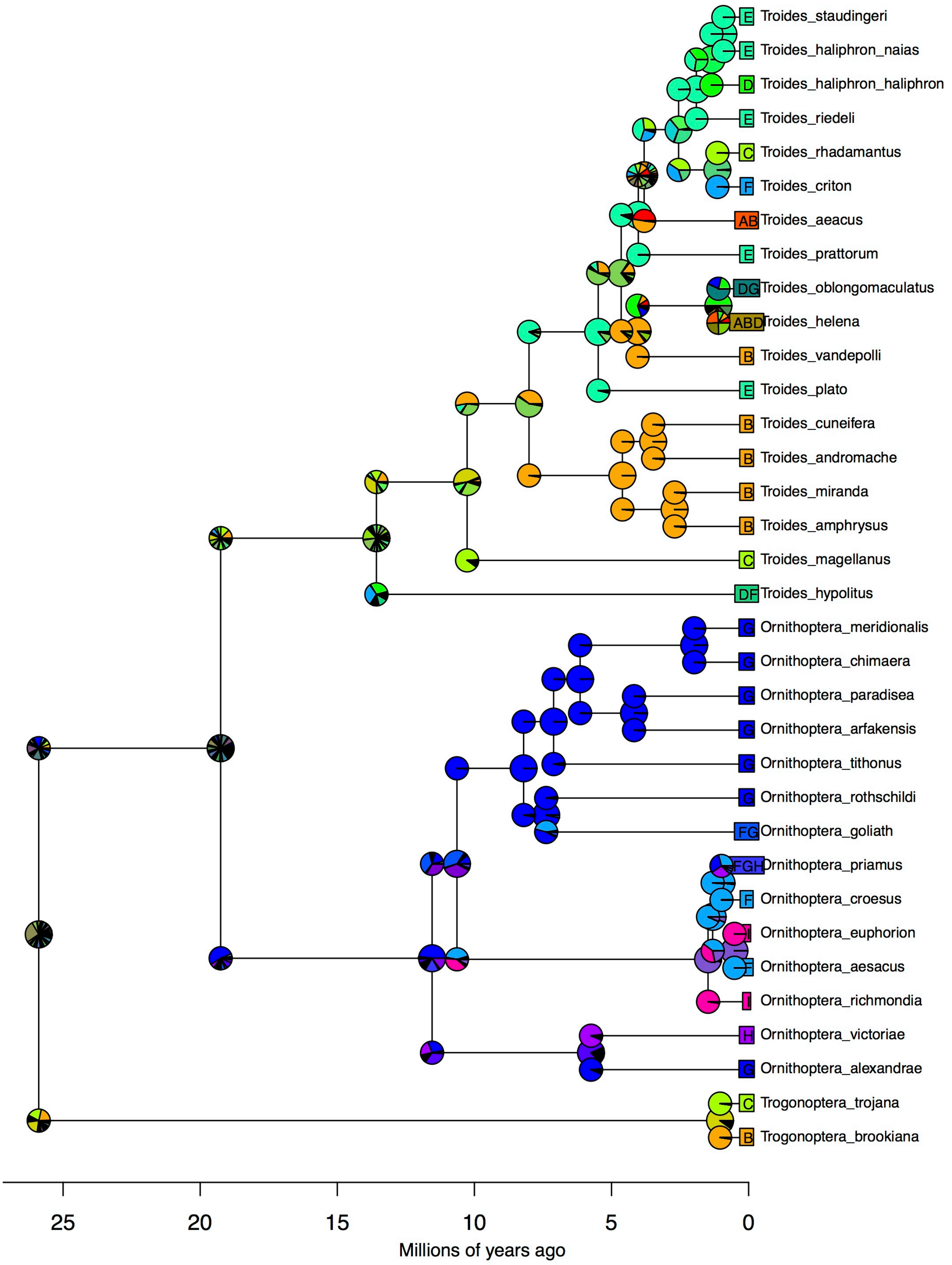

BioGeoBEARS DIVALIKE+J on Birdwings M0_unconstrained
ancstates: global optim, 3 areas max. d=0.0062; e=0; j=0.0413; LnL=−92.09

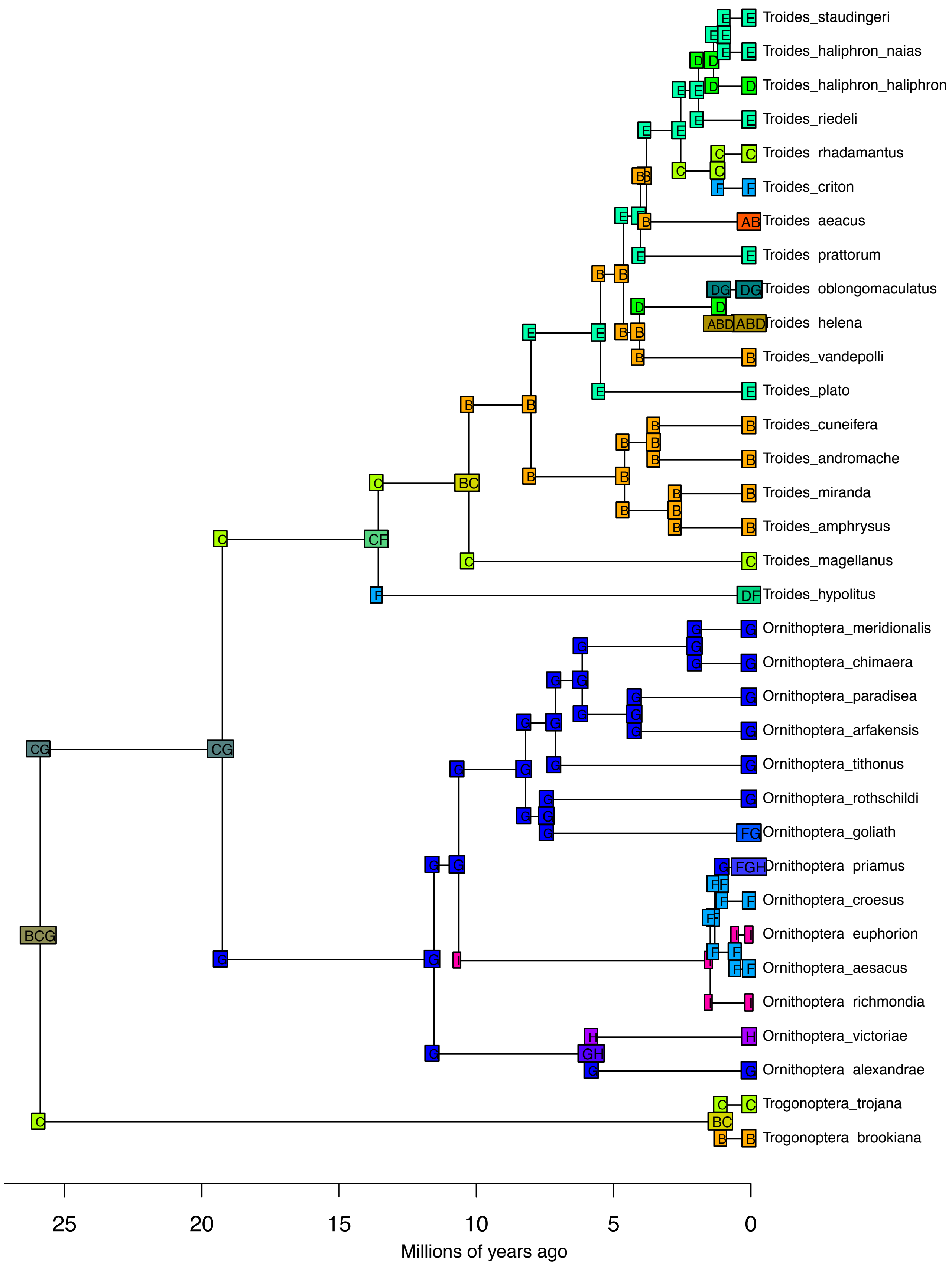

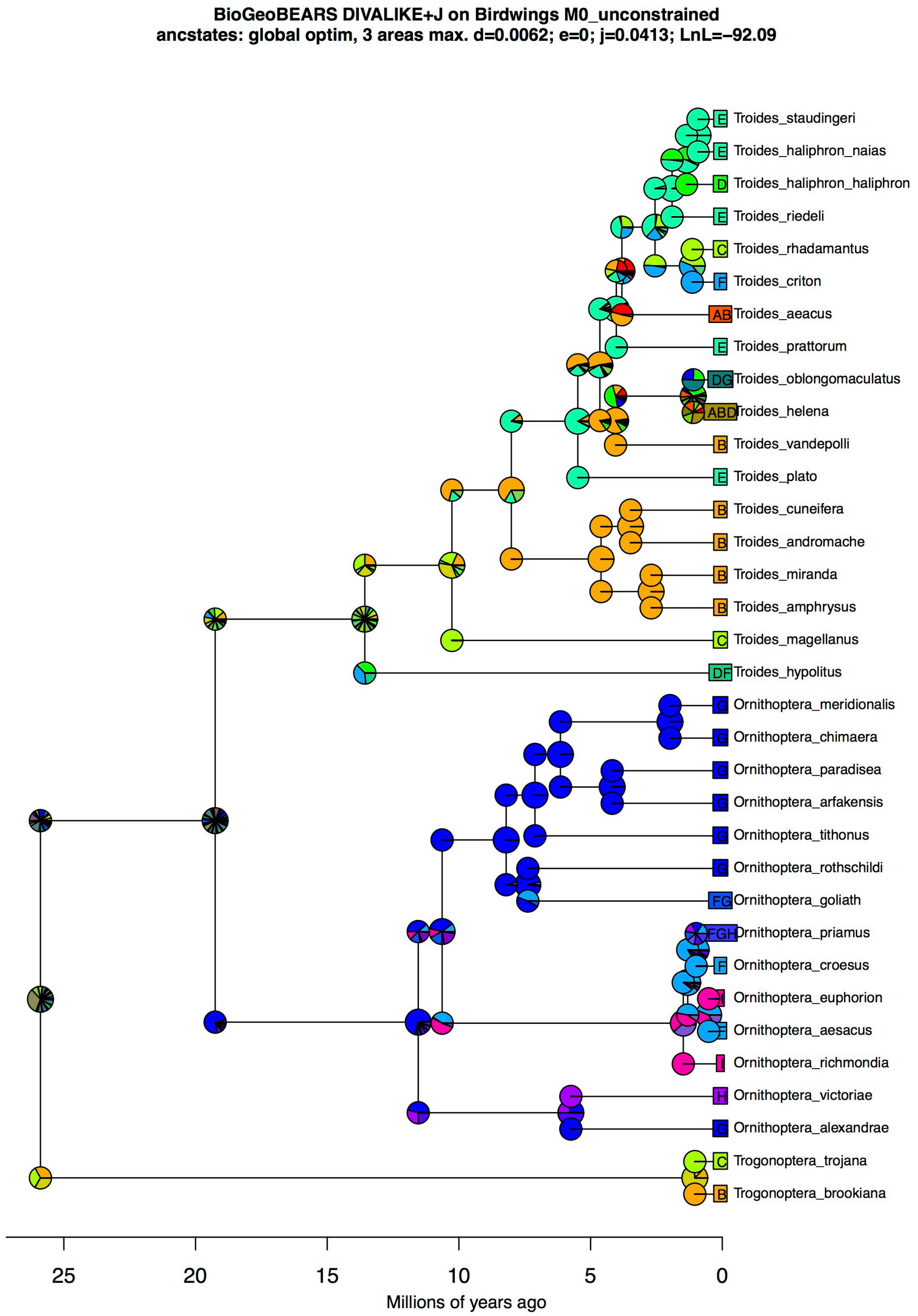
BioGeoBEARS DIVALIKE+J on Birdwings M0_unconstrained
ancstates: global optim, 3 areas max. d=0.0062; e=0; j=0.0413; LnL=-92.09
E Troides_staudingeri
E Troides_haliphron_naias
D Troides_haliphron_haliphron
E Troides_riedeli
C Troides_rhadamantus
F Troides_criton
AB Troides_aeacus
E Troides_prattorum
DG Troides_oblongomaculatus
ABD Troides_helena
B Troides_vandepolli
E Troides_plato
B Troides_cuneifera
B Troides_andromache
B Troides_miranda
B Troides_amphrysus
C Troides_magellanus
DF Troides_hypolitus
G Ornithoptera_meridionalis
G Ornithoptera_chimaera
G Ornithoptera_paradisea
G Ornithoptera_arfakensis
G Ornithoptera_tithonus
G Ornithoptera_rothschildi
FG Ornithoptera_goliath
FGH Ornithoptera_priamus
F Ornithoptera_croesus
I Ornithoptera_euphorion
F Ornithoptera_aesacus
I Ornithoptera_richmondia
H Ornithoptera_victoriae
G Ornithoptera_alexandrae
C Trogonoptera_trojana
B Trogonoptera_brookiana
25
20
15
10
5
0
Millions of years ago

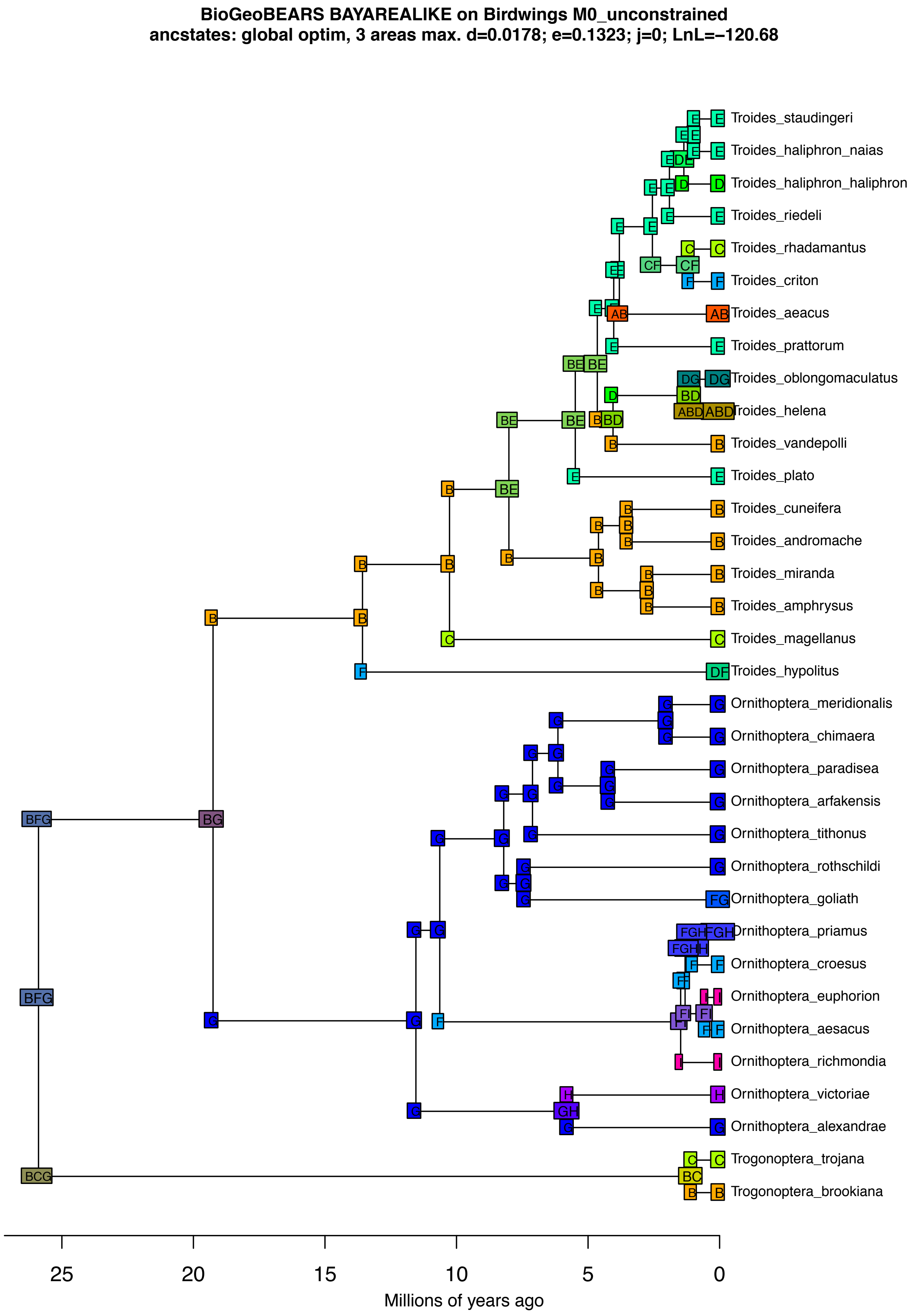
BioGeoBEARS BAYAREALIKE on Birdwings M0_unconstrained
ancstates: global optim, 3 areas max. d=0.0178; e=0.1323; j=0; LnL=-120.68
Troides_staudingeri
Troides_haliphron_naias
Troides_haliphron_haliphron
Troides_riedeli
Troides_rhadamantus
Troides_criton
Troides_aeacus
Troides_prattorum
Troides_oblongomaculatus
Troides_helena
Troides_vandepolli
Troides_plato
Troides_cuneifera
Troides_andromache
Troides_miranda
Troides_amphrysus
Troides_magellanus
Troides_hypolitus
Ornithoptera_meridionalis
Ornithoptera_chimaera
Ornithoptera_paradisea
Ornithoptera_arfakensis
Ornithoptera_tithonus
Ornithoptera_rothschildi
Ornithoptera_goliath
Ornithoptera_priamus
Ornithoptera_croesus
Ornithoptera_euphorion
Ornithoptera_aesacus
Ornithoptera_richmondia
Ornithoptera_victoriae
Ornithoptera_alexandrae
Trogonoptera_trojana
Trogonoptera_brookiana
25
20
15
10
5
0
Millions of years ago

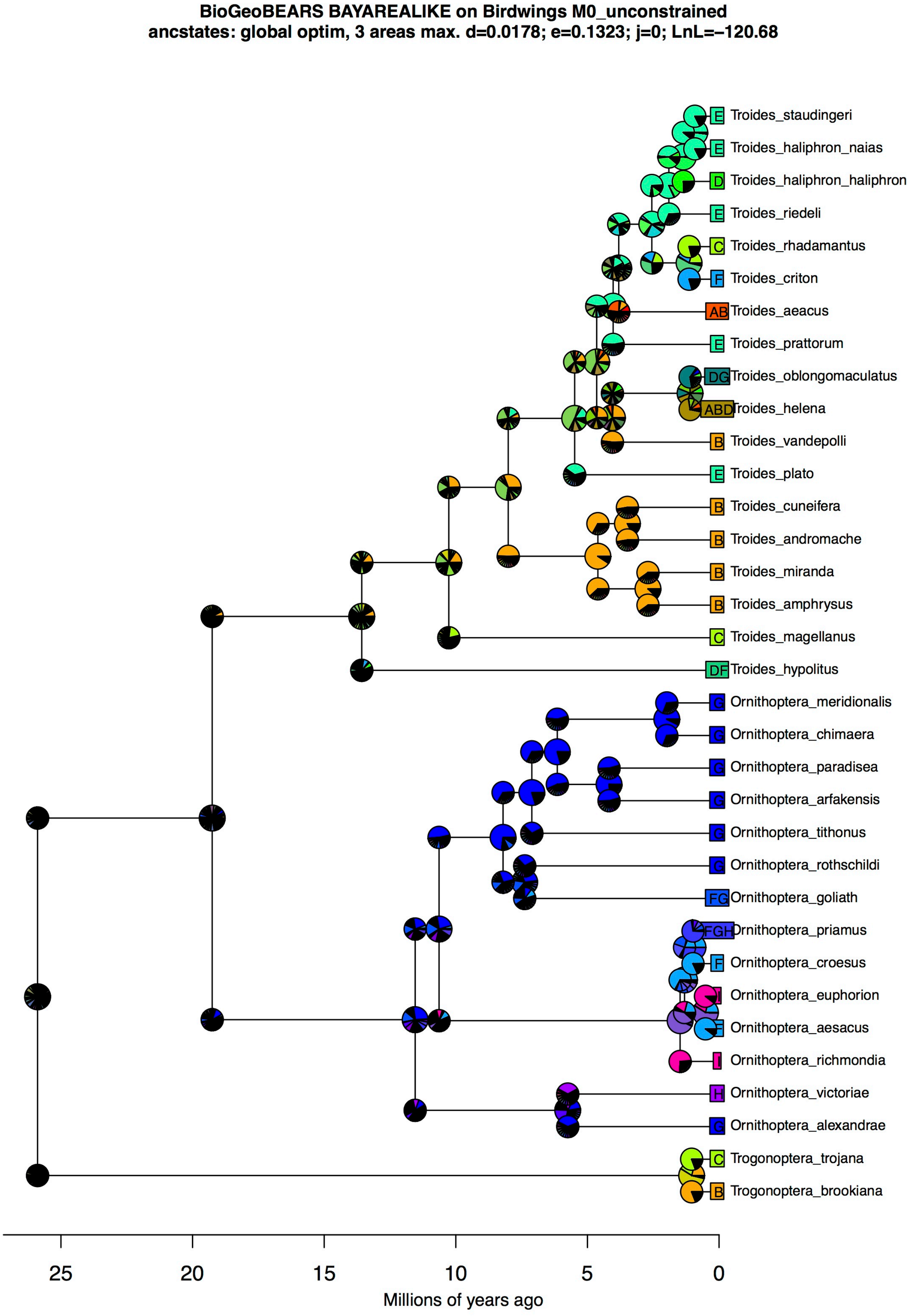

BioGeoBEARS BAYAREALIKE on Birdwings M0_unconstrained
ancstates: global optim, 3 areas max. d=0.0178; e=0.1323; j=0; LnL=-120.68
E Troides_staudingeri
E Troides_haliphron_naias
D Troides_haliphron_haliphron
E Troides_riedeli
C Troides_rhadamantus
F Troides_criton
AB Troides_aeacus
E Troides_prattorum
DG Troides_oblongomaculatus
ABD Troides_helena
B Troides_vandepolli
E Troides_plato
B Troides_cuneifera
B Troides_andromache
B Troides_miranda
B Troides_amphrysus
C Troides_magellanus
DF Troides_hypolitus
G Ornithoptera_meridionalis
G Ornithoptera_chimaera
G Ornithoptera_paradisea
G Ornithoptera_arfakensis
G Ornithoptera_tithonus
G Ornithoptera_rothschildi
FG Ornithoptera_goliath
FGH Ornithoptera_priamus
F Ornithoptera_croesus
I Ornithoptera_euphorion
F Ornithoptera_aesacus
I Ornithoptera_richmondia
H Ornithoptera_victoriae
G Ornithoptera_alexandrae
C Trogonoptera_trojana
B Trogonoptera_brookiana
25
20
15
10
5
0
Millions of years ago

BioGeoBEARS BAYAREALIKE+J on Birdwings M0_unconstrained
ancstates: global optim, 3 areas max. d=0.0043; e=0.0041; j=0.064; LnL=−97.15

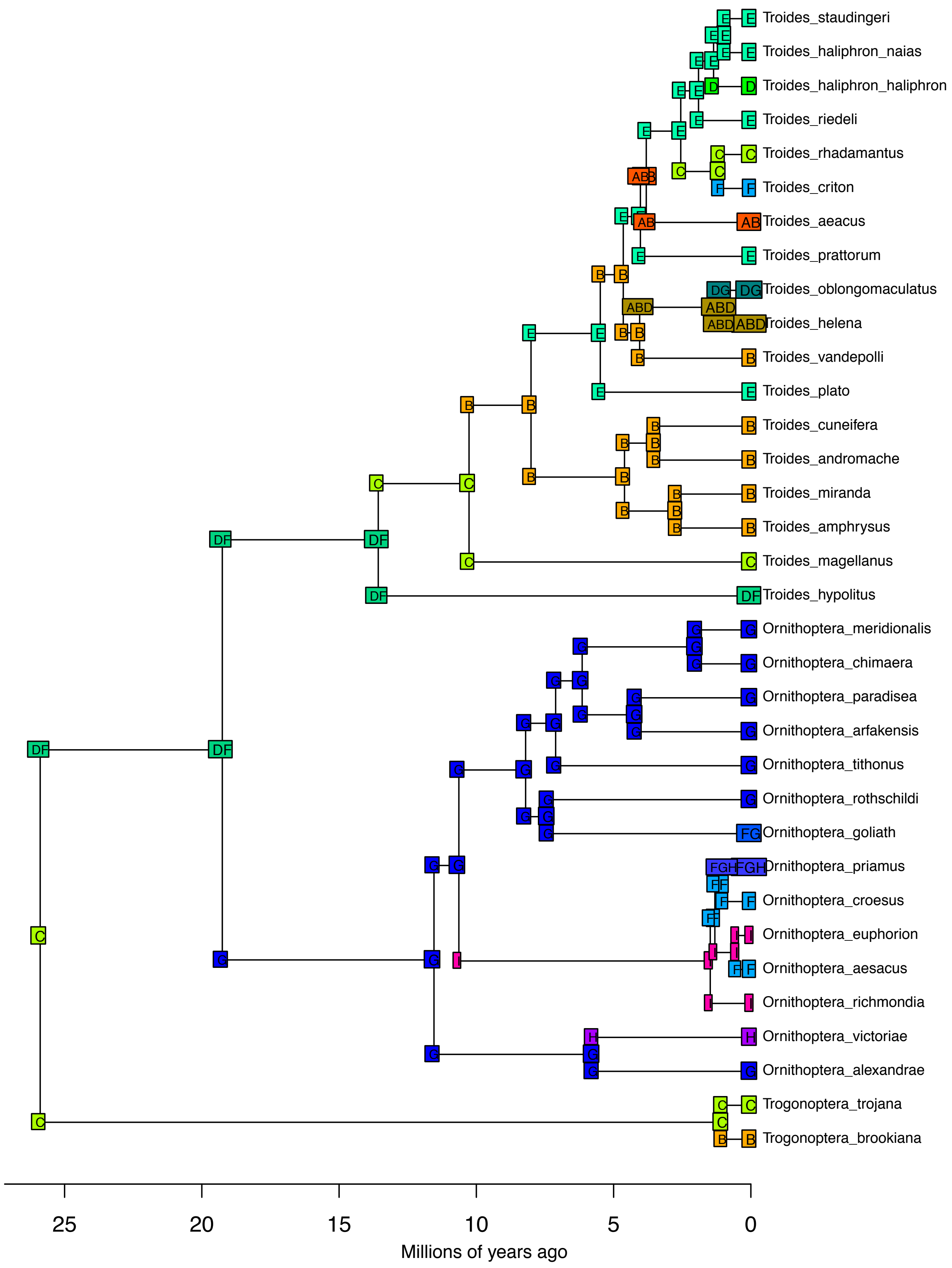

BioGeoBEARS BAYAREALIKE+J on Birdwings M0_unconstrained
ancstates: global optim, 3 areas max. d=0.0043; e=0.0041; j=0.064; LnL=−97.15

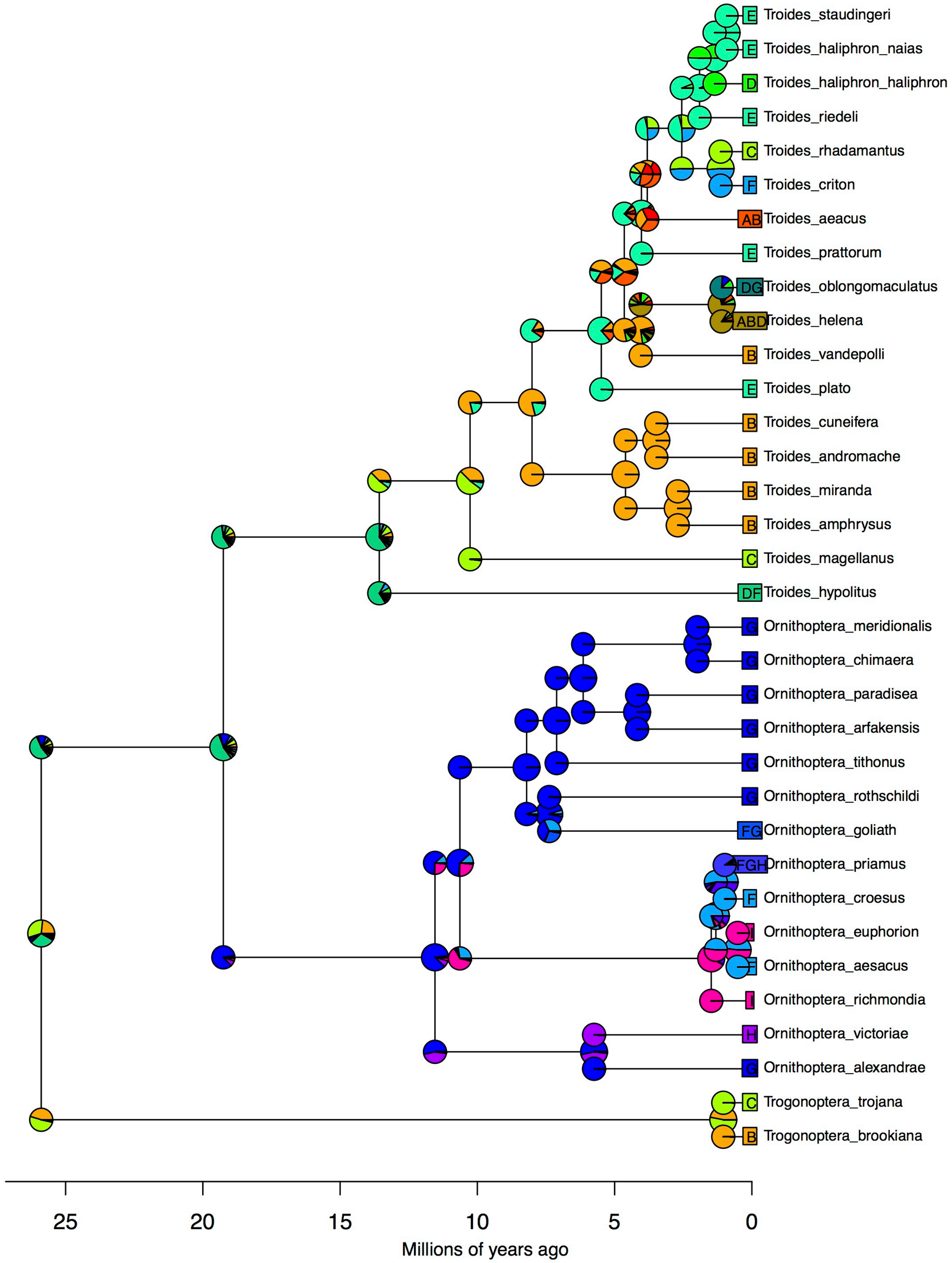

Figure S4. Lineages-through-time plots of birdwing butterflies. (a) Whole clade including the three genera. (b) Only the genus *Troides*. (c) Only the genus *Ornithoptera*. (d) Phylogeny of birdwing butterflies showing the three genera, and the timeline of their evolutionary history. M. Mioc., middle Miocene; L. Mioc., late Miocene; Pli., Pliocene. The Quaternary is the last period.

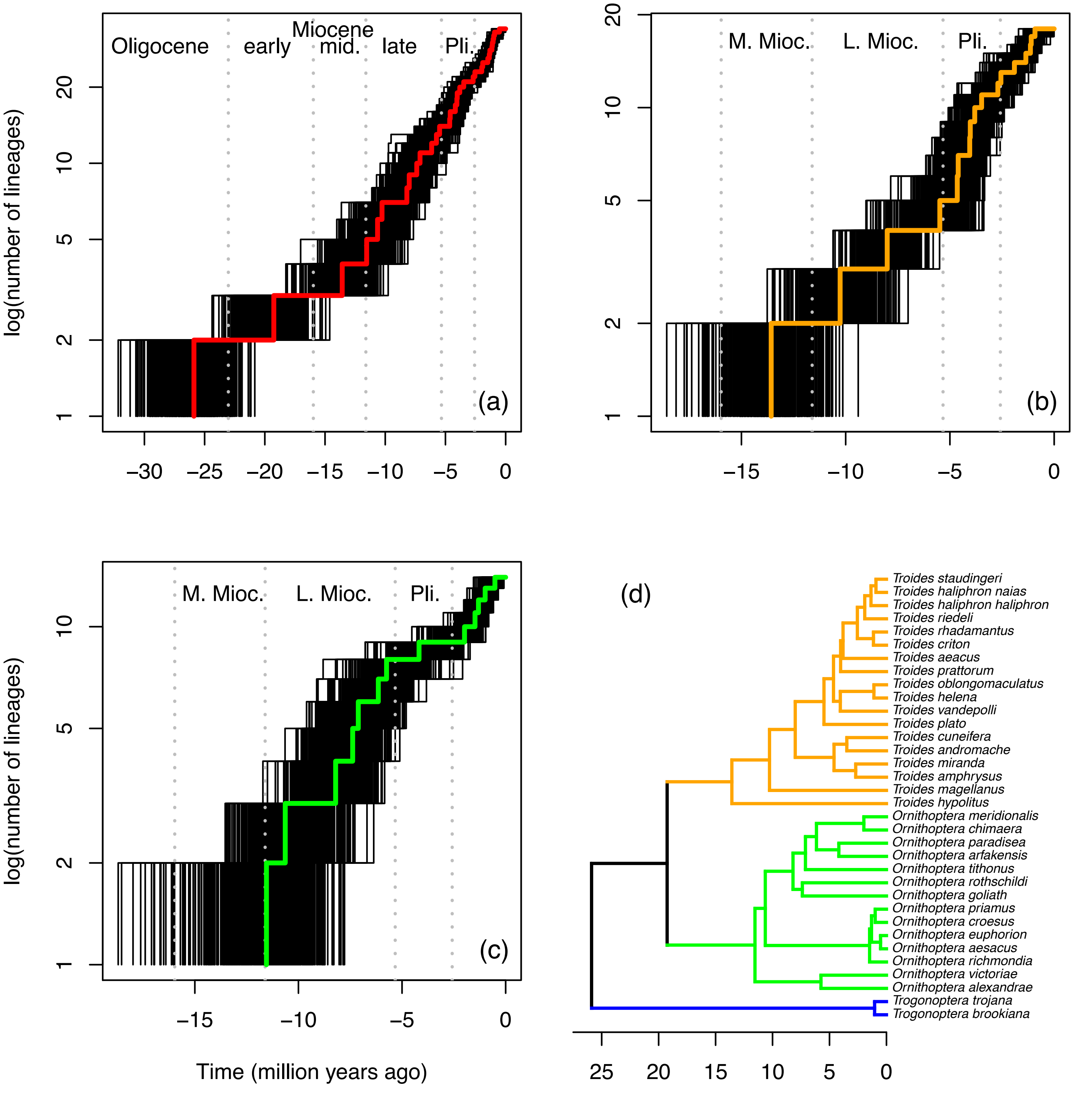

Figure S5. Bayesian MCMC of the best fitting GeoSSE model. (a) Estimate of speciation rate for both species occurring within and outside Wallacea (the rates are equal). (b) Estimate of extinction rate for both species occurring within and outside Wallacea (the rates are equal). (c) Estimate of transition rates (i.e. dispersal rates) out of Wallacea, and into Wallacea. (d) The rate out of Wallacea is higher than the other direction.

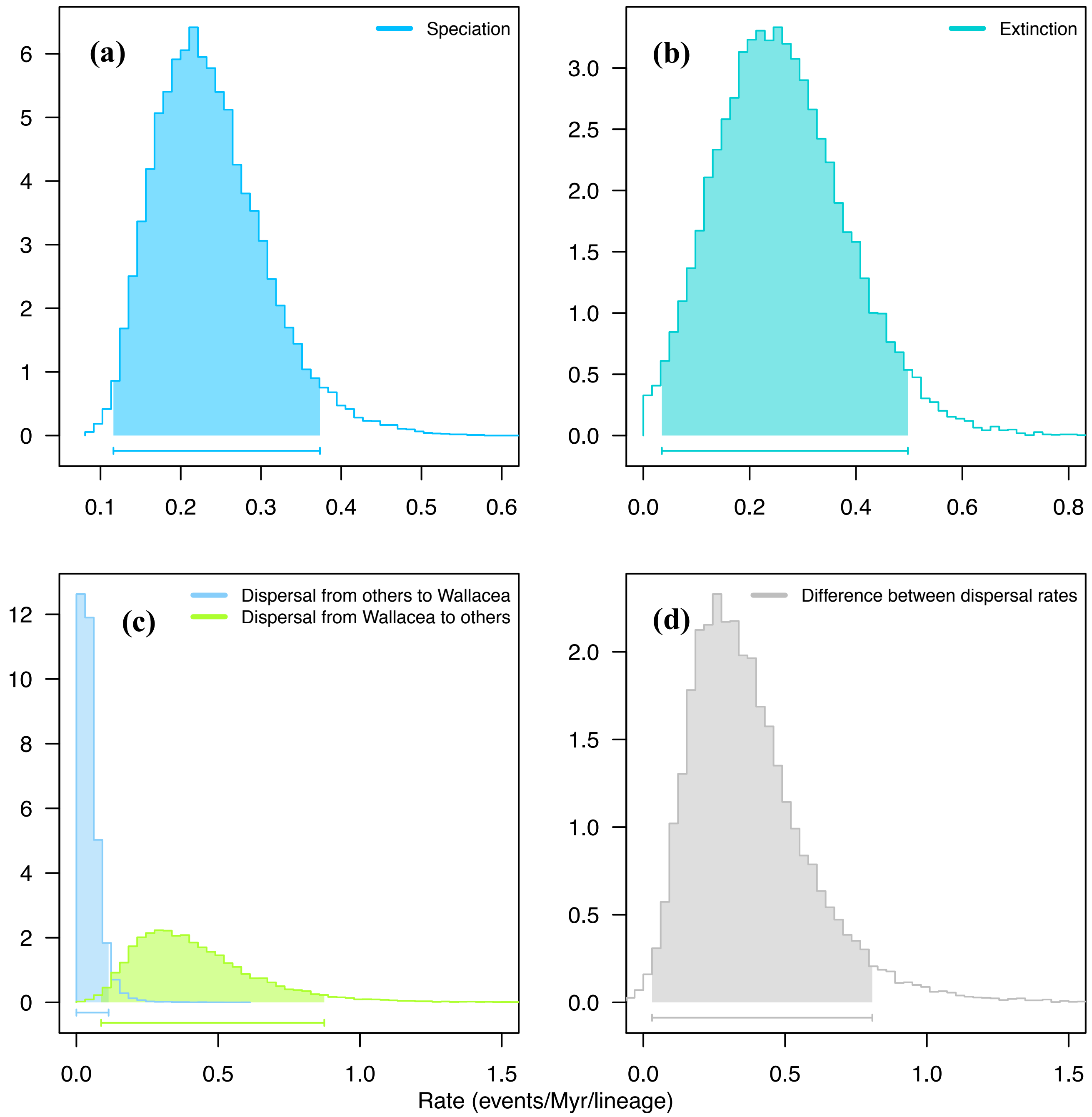

Supplement: Supplementary Information [file srep11860-s1.pdf]
